# Kdm6b-mediated epigenetic coordination of temporal precision during motor neuron differentiation

**DOI:** 10.1038/s44319-026-00808-2

**Published:** 2026-05-26

**Authors:** Haihan Ren, Qicheng Liu, Bingqian Li, Xuetong Yu, Dan Liang, Shuangling Li, Bing Pan, Qian Gao, Wenhong Deng, Jun-Ma Yu, Wenxian Wang

**Affiliations:** 1 School of Life Sciences, Anhui Medical University, Hefei, 230032 Anhui China; 2 Department of Obstetrics and Gynecology, NHC Key Laboratory of Study on Abnormal Gametes and Reproductive Tract, The First Affiliated Hospital of Anhui Medical University, Hefei, 230022 Anhui China; 3 Medical College, Hefei Vocational and Technical College, Hefei, 238000 Anhui China; 4 Department of General Surgery, The Second Affiliated Hospital of Anhui Medical University, Hefei, 230601 Anhui China; 5 Department of Anesthesiology, The Third Affiliated Hospital of Anhui Medical University (The First People’s Hospital of Hefei), Hefei, Anhui 230061 China

**Keywords:** Chromatin, Transcription & Genomics, Neuroscience

## Abstract

Histone modifiers are crucial for instructing multiple-stage cellular differentiation, yet the mechanisms underlying their temporal precision remain enigmatic. Here, we demonstrate that the H3K27 demethylase Kdm6b acts as an epigenetic regulator, coordinating stepwise motor neuron (MN) differentiation through sequential partnerships with stage-specific transcription factors (TFs). Genome-wide profiling reveals a progressive gain in Kdm6b occupancy, especially at distal regulatory elements, as differentiation proceeds. Kdm6b dynamically remodels chromatin landscapes by coordinating H3K27me3 removal with H3K27ac and H3K4me1 acquisition, thereby enabling timed gene activation from MN specification to maturation. Stage-specific inhibition of Kdm6b compromises the ordered expression of developmental genes. Mechanistically, Kdm6b interacts with temporal TFs over time to ensure precise transcriptional control and MN differentiation. Our work elucidates how a single epigenetic regulator achieves temporal fidelity of stepwise MN development, providing insight into epigenetic regulation of developmental timing.

## Synopsis

Kdm6b sequentially partners with stage-specific transcription factors to dynamically remodel chromatin landscapes, enabling temporally precise transcriptional programs and MN differentiation.

- Kdm6b binding progressively increases at distal regulatory elements as MN differentiation proceeds.
- Kdm6b dynamically remodels the chromatin landscape by coordinating H3K27me3 reduction with H3K27ac and H3K4me1 acquisition.
- Kdm6b sequentially partners with stage-specific TFs to precisely regulate gene transcription and drive stepwise MN differentiation.

## Abstract (web summary)

Kdm6b sequentially partners with stage-specific transcription factors to dynamically remodel chromatin landscapes, enabling temporally precise transcriptional programs and MN differentiation.

## Graphical abstract

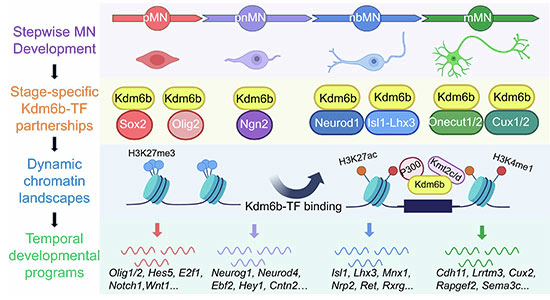

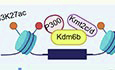

## Introduction

Cellular differentiation and diversity originating from multipotent or embryonic stem cells (ESCs) are meticulously orchestrated by multilayered regulatory networks that dynamically integrate genetic, epigenetic, and environmental cues. Central to these processes is transcriptional regulation, where sequence-specific transcription factors (TFs), including lineage-determining TFs, bind enhancers and promoters to modulate target gene expression through cooperation with cofactors, mediators and the basal transcriptional machinery (Richter et al, 2022; Roeder, 2019). Among these coregulators, histone modifiers recruited by TFs refine transcriptional programs by establishing diverse post-translational modifications (e.g., acetylation, methylation, and ubiquitination) on histone tails. These modifications establish specific chromatin signatures and corresponding gene activity states that underpin cell fate commitment (Morgan and Shilatifard, 2020; Podobinska et al, 2017; Yagi et al, 2025). Substantial progress has highlighted the critical role of TF-histone modifier interactions in cell lineage specification and differentiation (Fueyo et al, 2018; Hnisz et al, 2013; Lee et al, 2009; Zuryn et al, 2014). However, most studies have focused on initial and terminal cell states, with less attention paid to intermediate states or transient stages. Consequently, the dynamic mechanisms by which developmentally transient TFs recruit histone modifiers during stage transitions, and how their interplay drives stepwise cell differentiation, remain poorly understood.

Motor neuron (MN) development provides an excellent model for studying stepwise differentiation, progressing through a hierarchy of cellular states: MN progenitor (pMN), newborn MN (nbMN), and mature MN (mMN). Multipotent neural stem cells within the embryonic neuroepithelium, characterized by Sox2 expression, undergo ventral patterning via sonic hedgehog (Shh) signaling from the notochord and floor plate, leading to the pMN lineage commitment (Ribes and Briscoe, 2009). Subsequent specification yields nbMNs, which further differentiate into distinct mMN subtypes along the dorsoventral and rostrocaudal axes to innervate specific muscles (Arber, 2012; Philippidou and Dasen, 2013). These subtypes encompass the medial motor column (MMC; innervating axial muscles), hypaxial motor column (HMC; controlling body wall muscles), lateral motor column (LMC; innervating limb muscles), preganglionic motor column (PGC; controlling sympathetic ganglia) and others.

MN differentiation is highly controlled by a series of events, including transcriptional and post-transcriptional regulation (Amin et al, 2015; Carvelli et al, 2022) and developmental signals (Arber, 2012; Ribes and Briscoe, 2009). At the transcriptional level, a myriad of key developmental TFs have been extensively identified, which exhibit stage- and cell type-specific expression patterns essential for MN fate determination and differentiation. Olig2, a basic helix-loop-helix (bHLH) TF, is specifically expressed in pMNs and defines pMN identity (Lee et al, 2005; Novitch et al, 2001). Proneural TF Ngn2 (Neurog2) is transiently expressed in late pMNs, initiating MN specification and suppressing alternative cell fates (Guillemot, 2007; Lee et al, 2005). Strikingly, Ngn2 is important for the induction of later developmental TFs such as Mnx1, Isl1, Ebfs and Onecuts (Velasco et al, 2017). Another proneural TFs, Neurod1/2/4 were typically expressed in post-mitotic MNs and contribute to neuronal differentiation and maturation (Gao et al, 2009; Guillemot, 2007). Mnx1 (Hb9, a homeodomain protein) acts as a master regulator and definitive marker of post-mitotic MNs, driving MN-specific genes and suppressing interneuron programs (Arber et al, 1999). Isl1 and Lhx3 are LIM-homeodomain TFs forming a complex (Isl1-Lhx3) crucial for generic MN differentiation and MMC subtype specification (Mazzoni et al, 2013; Thaler et al, 2002). This complex cooperates with Mnx1 to establish the core MN transcriptional program, including genes for neurotransmitter synthesis and axon pathfinding. In addition, forkhead box TF Foxp1 has been identified as necessary for LMC and PGC specification (Dasen et al, 2008; Rousso et al, 2008). Interestingly, in HMC neurons lacking Lhx3 or Foxp1, Onecut TFs interact with Isl1 to maintain the core MN gene transcriptional program (Rhee et al, 2016). Thus, these developmental TFs demarcate spatiotemporal MN subgroups, orchestrating precise transcriptional regulation and cell specification.

Histone modifiers establish chromatin contexts crucial for transcriptional regulation and neural development (Ciceri et al, 2024; Matlik et al, 2023; Ramesh et al, 2023; Tang et al, 2020; Wijayatunge et al, 2018; Xu et al, 2025). While histone acetylation (e.g., H3K27ac, catalyzed by P300/CBP) is broadly associated with activation, histone methylation exhibits complex site- and state-dependent effects. Repressive H3K27 trimethylation (H3K27me3) catalyzed by PRC2 is reversibly removed by Kdm6a/b (UTX/Jmjd3) for gene activation (Arcipowski et al, 2016; Wang et al, 2020). Conversely, H3K4 mono-/di-/trimethylation (H3K4me1/2/3, catalyzed by Kmt2a/b/c/d and Setd1a/b) is linked to activation, with H3K4me1 prominently enriched at enhancers and H3K4me3 at promoters, respectively (Barski et al, 2007; Heintzman et al, 2007). Notably, Combinations of histone modifications create specific chromatin architectures and regulatory varieties. For instance, bivalent promoters marked by H3K4me3 and H3K27me3 in ESCs silence developmental genes and concurrently poise them for activation upon lineage induction (Bernstein et al, 2006), while the combination of H3K27ac and H3K4me1 defines active enhancers for gene activation (Creyghton et al, 2010). Despite the well-established roles of developmental TFs in MN development, the involvement of histone modifiers has been less extensively investigated. CBP/P300 interacts with Ngn2 and retinoic acid receptors to influence MN specification and axonal projection (Lee et al, 2009). The PRC1 component Bmi1 regulates MN subtype differentiation dose-dependently via Hox genes (Golden and Dasen, 2012). Our recent work identified Kdm6b as a driver of MN differentiation and diversification through its interaction with the Isl1-Lhx3 complex (Wang et al, 2022). However, the dynamic histone modifier-TF interplays and their integrated actions to orchestrate the gene regulatory network underlying stepwise MN development remain elusive.

In this study, we leveraged an ESC-to-MN directed differentiation system recapitulating the stepwise process of MN development. Genome-wide mapping revealed Kdm6b occupancy is dynamic, progressively enriched at distal enhancers and accompanied by an increase in regulatory elements as MN differentiation progresses. Kdm6b binding dynamically remodeled the chromatin landscape, driving rapid loss of the repressive mark H3K27me3 with concomitant gains in the activating marks H3K27ac and H3K4me1 at occupied sites. However, Kdm6b dissociation triggered the chromatin configuration towards the repressive state, with H3K27me3 restoration and H3K27ac and H3K4me1 reduction. Transcriptome analyses implicated Kdm6b target genes in widespread processes crucial throughout MN development, including cell cycle regulation, pattern specification, regionalization, Wnt and Notch signaling, neurogenesis, axonogenesis, neuron projection, cell junction, synaptogenesis and dendritogenesis. Pharmacological inhibition of Kdm6b enzymatic activity significantly disrupted stage-specific transcriptional profiles. Furthermore, Kdm6b was found to associate sequentially with stage-specific transcription factors, including Sox2, Olig2, Ngn2, Neurod1, Isl1-Lhx3, Onecut1/2, and Cux1/2, across MN differentiation. Together, these dynamic Kdm6b-TF partnerships and the adaptable chromatin landscapes they coordinate establish an epigenetic framework essential for the precise control of stepwise MN differentiation. Our study provides a comprehensive understanding of Kdm6b during stepwise MN differentiation and underscores the pivotal role of a histone modifier in achieving temporal precision in cell development.

## Results

### In vitro directed motor neuron differentiation recapitulates developmental progression

**Figure 1:**
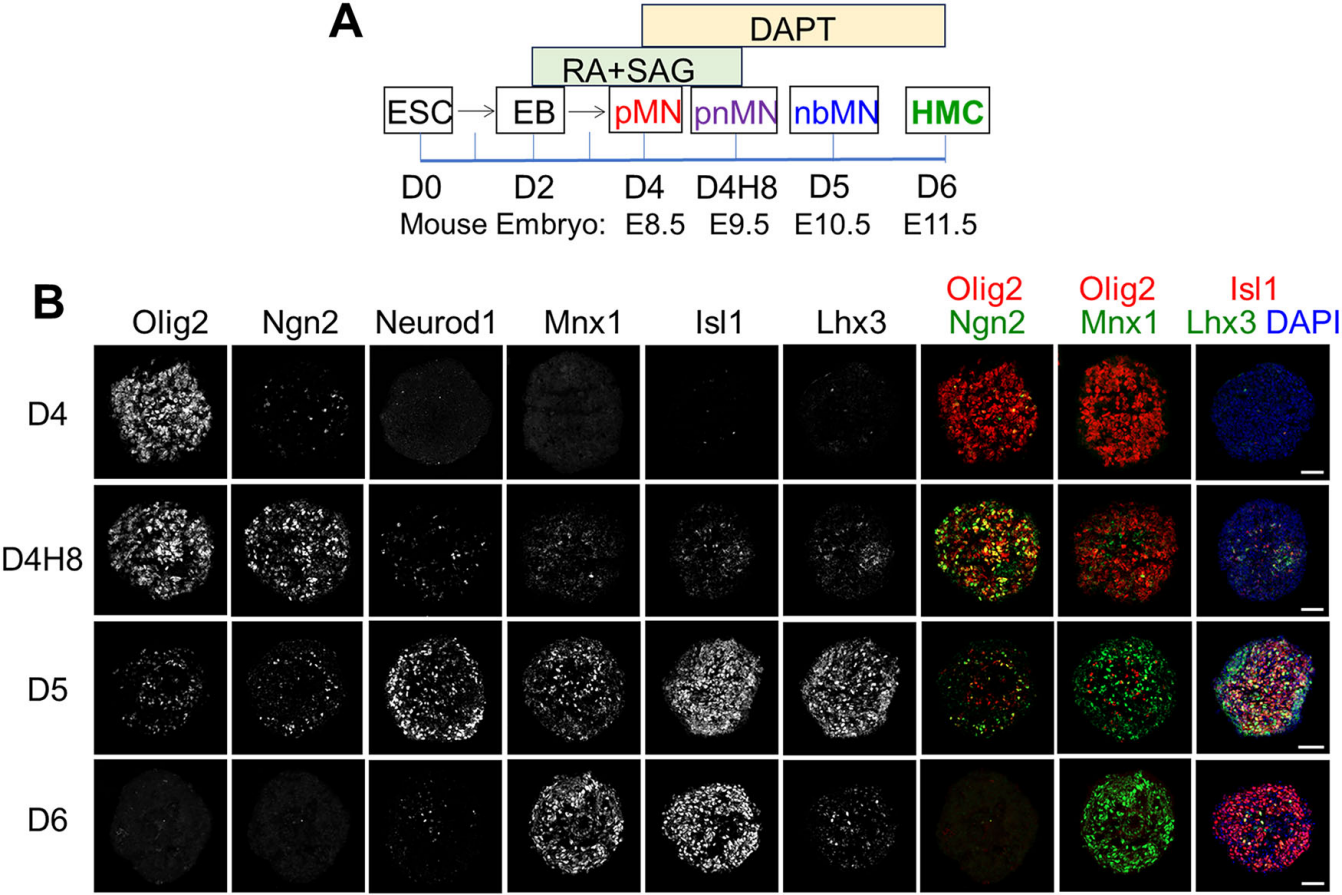
In vitro directed MN differentiation recapitulates in vivo developmental progression. (**A**) Schematic of the in vitro ESC→MN (HMC) differentiation process, with timepoints approximating mouse embryonic stages. ESC embryonic stem cell, MN motor neuron, EB embryonic body, pMN motor neuron progenitor, pnMN proneural motor neuron, nbMN newborn motor neuron, HMC hypaxial motor column. D day, E embryonic day. (**B**) Immunostaining of Olig2, Ngn2, Neurod1, Mnx1, Isl1, and Lhx3 at MN differentiation stages D4, D4H8, D5, and D6. Scale bars: 50 μm.

**Figure EV1:**
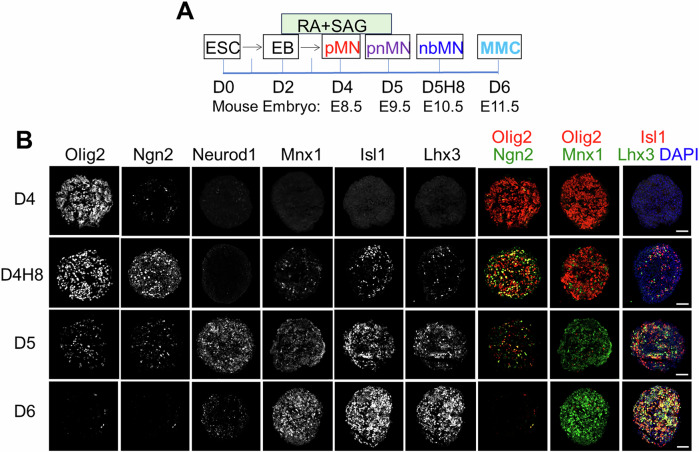
In vitro directed MN differentiation recapitulates in vivo developmental progression, related to Fig. 1. (**A**) The diagram represents the in vitro ESC→MN (MMC) differentiation process, with each timepoint approximately corresponding to the mouse embryonic stage. ESC embryonic stem cell, MN motor neuron, EB embryonic body, pMN motor neuron progenitor, pnMN proneural motor neuron, nbMN newborn motor neuron, MMC medial motor column. D day, E embryonic day. (**B**) Immunostaining of Olig2, Ngn2, Neurod1, Mnx1, Isl1, and Lhx3 at MN differentiation stages D4, D5, D5H8, and D6H8. Scale bars: 50 μm.

MN differentiation is a tightly regulated process enabling the spatiotemporal generation of developmentally and functionally distinct MN subtypes, including pMNs, nbMNs and diverse mMNs. Kdm6b was previously implicated in interacting with the Isl1-Lhx3 complex to regulate key developmental genes (such as Isl1, Lhx3, Mnx1 and Foxp1), thereby influencing MN differentiation and subtype diversification (Wang et al, 2022). However, differentially expressed genes (DEGs) in Kdm6b-null mice exhibited remarkable heterogeneity across different MN subtypes. Crucially, the majority of these DEGs were not known targets of Isl1-Lhx3, suggesting the involvement of alternative regulatory mechanisms. To overcome the challenge of obtaining sufficient numbers of highly pure, specific MN subtypes from mice for mechanistic studies, we utilized an established directed MN differentiation strategy from mouse ESCs (Tan et al, 2016; Wichterle and Peljto, 2008). This in vitro system recapitulates key stages of in vivo MN development, characterized by sequential expression of developmental TFs: Olig2, Ngn2, Neurod1, Mnx1, Isl1 and Lhx3 (Figs. 1 and EV1). Both MMC and HMC differentiation trajectories progressed through pMN, pnMN (proneural motor neuron), nbMN and mMN stages (Figs. 1A and EV1A). Treatment with Notch signaling inhibitor DAPT accelerated differentiation and led to downregulation of Lhx3 at the terminal mMN stage (Fig. 1B), in alignment with previous findings (Rhee et al, 2016; Tan et al, 2016). For the HMC differentiation process (Fig. 1B), Olig2 was prominently expressed in pMNs at day 4 (D4). After 8 h (D4H8), a significant proportion of cells expressed proneural TFs Ngn2 and Neurod1, indicating transition towards cell cycle exit and neurogenesis initiation. Particularly, Ngn2 exhibited the highest expression level at this stage throughout development. We designate MNs at this stage as proneural MNs (pnMNs), highlighting this transient stage characterized by high Ngn2 expression. Consistent with this early specification phase, key MN determinants Mnx1, Isl1 and Lhx3 were expressed at very low levels. Notably, three distinct cell populations were observed at D4H8: Olig2+Ngn2-, Olig2+Ngn2+, and Olig2-Ngn2+, indicating incomplete synchronization and a mixture of pMNs and pnMNs. At Day 5 (D5), expression of Olig2 and Ngn2 rapidly declined, while Mnx1, Isl1 and Lhx3 expression increased, marking entry into the post-mitotic stage and the establishment of nbMN identity. In contrast to other TFs, Neurod1 expression culminated specifically at this nbMN stage, serving as a robust marker for nbMN. One day later (D6), Olig2 and Ngn2 expression was nearly absent and Neurod1 was drastically downregulated. Concurrently, Mnx1 and Isl1 reached high expression levels, signifying developmental progression towards MN maturation. Lhx3 expression was downregulated but not completely absent at this stage. Collectively, the in vitro directed differentiation process effectively models the temporal progression of in vivo MN development, generating cells with sequentially committed identities.

### Kdm6b exhibits dynamic genome-wide binding patterns during MN development

**Figure 2:**
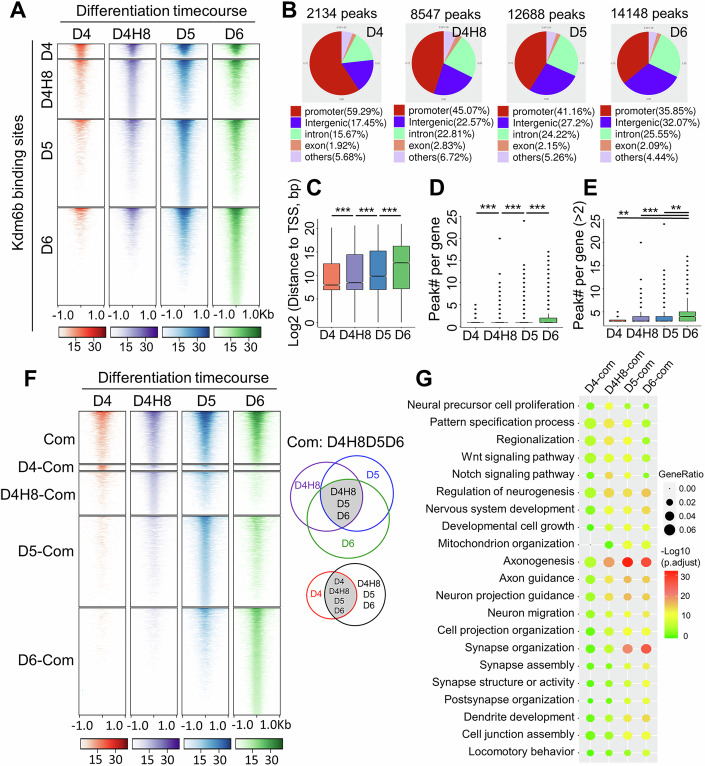
Genome-wide enrichment of Kdm6b reveals a dynamic binding pattern during MN development. (**A**) Kdm6b binding sites at the indicated stages (Y-axis) show differential enrichment across the MN differentiation timecourse (X-axis). The color bar shows relative enrichment intensity. (**B**) Genomic distribution of Kdm6b binding sites at the indicated stages, revealing a progressive increase toward intergenic and intron regions as differentiation proceeds. (**C**) Box plots showing the distance of Kdm6b binding sites to the nearest TTSs at the indicated stages, each from one Cut&Run assay (*n* = 1). *P* value was determined by the Wilcox rank-sum test with significance as ***p < 0.001 (from left to right: 4.3e-11, 7.4e-15, and 1.0e-53). The specific parameters for each box plot (minimum, 25th percentile, median, 75th percentile, maximum) are: D4 (0, 6.9, 8.0, 12.5, 20.2), D4H8 (0, 7.0, 8.5, 14.4, 20.7), D5 (0, 7.0, 9.9, 15.2, 20.9), and D6 (0, 7.2, 12.7, 16.2, 21.0). (**D**) Box plots quantifying the number of Kdm6b binding peaks per proximal gene at the indicated stages, each from one Cut&Run assay (*n* = 1). *P* value was determined by the Wilcox rank-sum test with significance as ***p < 0.001 (from left to right: 4.2e-29, 2.2e-27, 1.6e-5). The specific parameters for each box plot (minimum, 25th percentile, median, 75th percentile, maximum) are: D4 (1, 1, 1, 1, 5), D4H8 (1, 1, 1, 1, 20), D5 (1, 1, 1, 1, 24), and D6 (1, 1, 1, 2, 17). (**E**) Box plots quantifying the number of >2 Kdm6b binding peaks per proximal gene at the indicated stages, each from one Cut&Run assay (*n* = 1). *P* value was determined by the Wilcox rank-sum test with significance as **p < 0.01; ***p < 0.001 (D6 vs D4: 0.009; D6 vs D4H8: 5.4e-7; D6 vs D5: 0.002). The specific parameters for each box plot (minimum, 25th percentile, median, 75th percentile, maximum) are: D4 (3, 3, 3, 3.25, 5), D4H8 (3, 3, 3, 4, 20), D5 (3, 3, 3, 4, 24), and D6 (3, 3, 4, 5, 17). (**F**) Enrichment profiles of Kdm6b’s common peaks (Com: shaded region of Venn Diagram shared by D4H8, D5, and D6) and stage-specific peaks (D4-Com, D4H8-Com, D5-Com, and D6-Com) (Y-axis) across MN differentiation timecourse (X-axis). The color bar shows relative enrichment intensity. (**G**) GO analyses of stage-specific target genes showing top-ranked enrichment pathways with bubble plot. Biological Process terms are shown. Adjusted *p* value was determined by the Wilcox rank-sum test. Source data are available online for this figure.

**Figure EV2:**
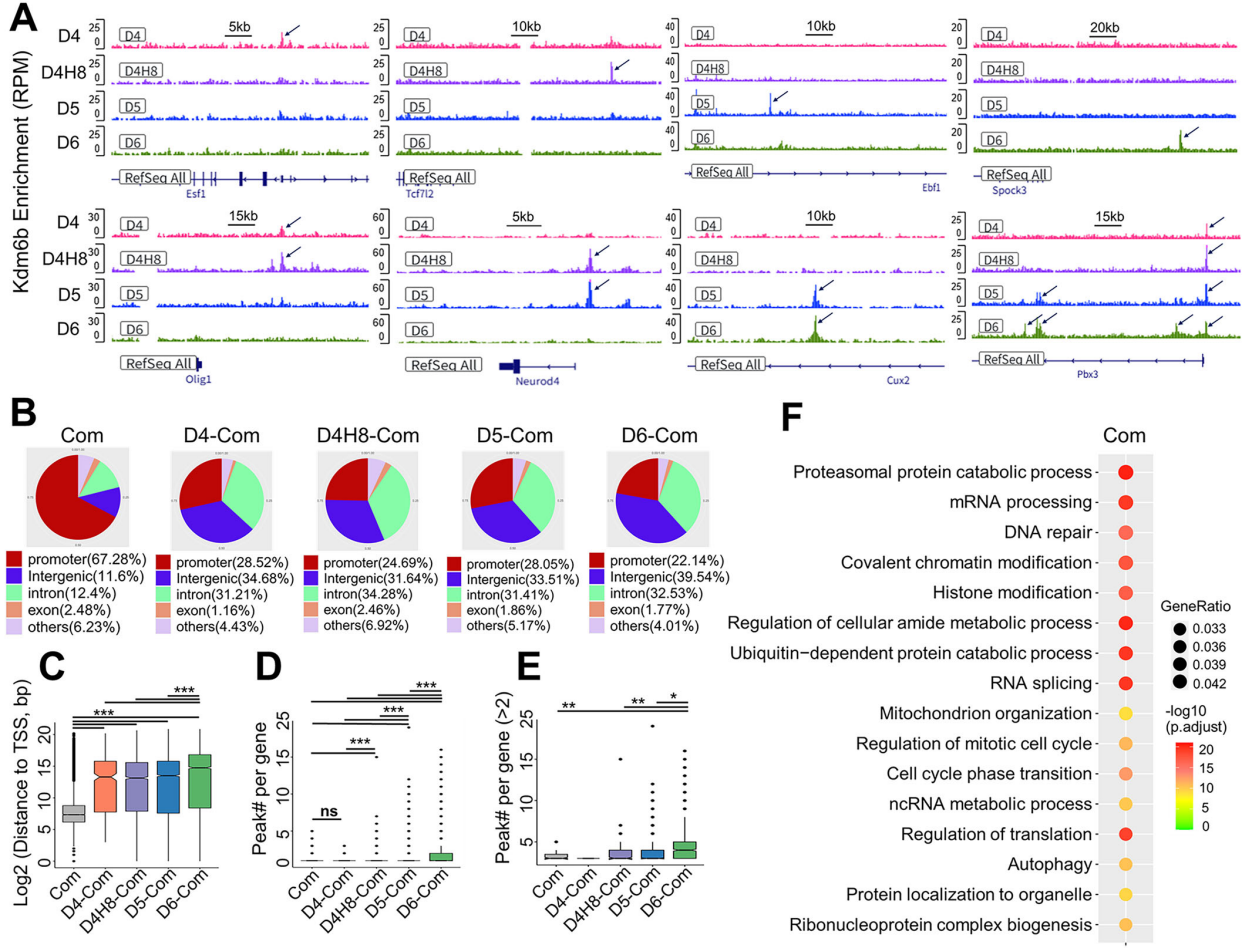
Dynamic binding patterns of Kdm6b correlate with preferential spatial occupancy and distinct functional significance, related to Fig. 2. (**A**) Genome browser tracks displaying dynamic Kdm6b enrichment at the selected gene loci across MN developmental stages. RPM indicates enrichment intensity. (**B**) The genomic distribution of Kdm6b common (Com) and stage-specific binding peaks (D4-Com, D4H8-Com, D5-Com and D6-Com) exhibiting spatial preference. (**C**) Box plots showing the distance of Kdm6b common and stage-specific binding sites to the nearest TSSs. *P* value was determined by the Wilcox rank-sum test, and statistical significance was indicated as follows. ***p < 0.001 (D6-Com vs Com: 0; D6-Com vs D4-Com: 3.2e-8; D6-Com vs D4H8-Com: 3.9e-54; D6-Com vs D5-Com: 4.3e-73). The specific parameters for each box plot (minimum, 25th percentile, median, 75th percentile, maximum) are: Com (0, 6.2, 7.3, 8.8, 20.2), D4-Com (3, 7.8, 13.3, 15.8, 20.2), D4H8-Com (0, 7.9, 13.1, 15.6, 20.7), D5-Com (0, 7.6, 13.5, 15.8, 20.9), and D6-Com (0, 8.4, 14.8, 16.8, 20.9). The data were from one Cut&Run assay at each stage (*n* = 1). (**D**) Box plots quantifying the number of Kdm6b binding peaks per proximal gene. *P* value was determined by the Wilcox rank-sum test. ns nonsignificant; ***P < 0.001 (D4H8-Com vs D4-Com: 1.9e-7; D5-Com vs D4H8-Com: 8.6e-21; D6-Com vs D5-Com: 4.2e-7). The specific parameters for each box plot (minimum, 25th percentile, median, 75th percentile, maximum) are: Com (0, 1, 1, 1, 5), D4-Com (0, 1, 1, 1, 3), D4H8-Com (0, 1, 1, 1, 15), D5-Com (0, 1, 1, 1, 19), and D6-Com (0, 1, 1, 2, 16). The data were from one Cut&Run assay at each stage (*n* = 1). (**E**) Box plots quantifying the number of >2 Kdm6b binding peaks per proximal gene. *P* value was determined by the Wilcox rank-sum test. *P < 0.05; **P < 0.01 (D6-Com vs Com: 0.002; D6-Com vs D4H8-Com: 0.003; D6-Com vs D5-Com: 0.04). The specific parameters for each box plot (minimum, 25th percentile, median, 75th percentile, maximum) are: Com (3, 3, 3, 3, 5), D4-Com (3, 3, 3, 3, 3), D4H8-Com (3, 3, 3, 4, 15), D5-Com (3, 3, 3, 4, 19), and D6-Com (3, 3, 4, 5, 16). The data were from one Cut&Run assay at each stage (*n* = 1). (**F**) GO analyses of common target genes showing top-ranked enrichment pathways with bubble plot. Biological Process terms are shown.

Given that Kdm6b-interacting TFs (Isl1-Lhx3 complex) was previously characterized in MMC subtypes, this study focused on the HMC developmental trajectory. To investigate Kdm6b genomic configuration across MN development, we performed Cut&Run coupled with high-throughput sequencing (Cut&Run-seq) to map its genome-wide binding landscape. Kdm6b exhibited stage-specific enrichment dynamics, characterized by both unique sites at individual stages and shared sites among multiple stages (Figs. 2A and EV2A). Analysis of the binding distribution revealed a progressive increase in total occupancy, with the majority localized to promoters, introns, and intergenic areas (Fig. 2B). Binding sites at intronic and intergenic regions rose from 707 (33.12% of total) at D4 to 3879 (45.38%) at D4H8, 6524 (51.42%) at D5, and 8152 (57.62%) at D6 (Fig. 2B). In contrast, promoter-proximal binding sites remained numerically stable from D4H8 through D6 (ranging from 5067 to 5077), despite a proportional decrease (Fig. 2B). These results indicate that the progressive gain of Kdm6b binding sites at distal regulatory elements primarily drives the dynamic redistribution of Kdm6b across differentiation. Accordingly, the median distance from each Kdm6b binding site to the nearest transcription start site (TSS) increased throughout differentiation (Fig. 2C). Concomitantly, the number of Kdm6b binding peaks per target gene gradually increased, with mMNs at D6 showing pronounced enrichment for genes harboring >2 binding sites (Fig. 2D,E).

Since different binding patterns likely confer differential functional outcomes, we categorized kdm6b binding sites into two subgroups, common peaks (Com) and stage-specific peaks (Fig. 2F). Common peaks were defined as binding sites shared across D4H8, D5, and D6 as D4 peaks were scarce and 87.3% of them (1863 out of 2134) overlapped with D4H8 peaks. Stage-specific peaks were obtained by subtracting common peaks from total peaks at each stage, designated as D4-Com, D4H8-Com, D5-Com, and D6-Com, respectively. Stage-specific peaks showed minimal overlap between distal stages (e.g., D4-Com versus D6-Com) but partial overlap between consecutive stages (e.g., D4H8-Com versus D5-Com) (Fig. 2F), consistent with observed cellular asynchrony (Fig. 1B). Intriguingly, common peaks were predominantly promoter-proximal, while stage-specific peaks were enriched in distal intronic and intergenic regions, indicating preferential binding to cis-regulatory elements distributed within distinct genomic regions (Fig. EV2B). Additionally, D6-Com peaks exhibited the greatest binding distance to the nearest TSS and the highest number of binding sites per TSS, similar to the enrichment pattern of total binding peaks (Fig. EV2C–E). Taken together, the enrichment architecture reveals that Kdm6b dynamically binds the genome throughout MN differentiation, strategically occupying distal enhancers and multiple regulatory elements during neuronal maturation.

Next, we sought to annotate the functional relevance associated with this dynamic Kdm6b binding pattern. Putative targets of Kdm6b were assigned as common or stage-specific target genes corresponding to common or stage-specific binding peaks, respectively. Gene ontology (GO) analyses revealed that common target genes were mainly enriched in fundamental cellular processes, including protein catabolism, RNA processing and splicing, DNA repair, and chromatin/histone modification (Fig. EV2F). In contrast, stage-specific target genes were strikingly associated with neuronal development and differentiation (Fig. 2G). D4-Com and D4H8-Com target genes were related to cell proliferation, pattern specification, regionalization, Wnt and Notch signaling, and neurogenesis, manifesting features characteristic of cell proliferation, identity commitment and initial neurogenesis during early neuronal development within pMNs and pnMNs. D5-Com target genes were tightly linked to neurogenesis, nervous system development, cell growth, mitochondrial organization, axonogenesis and axon guidance, reflecting potential regulatory roles of Kdm6b in MN neuronal differentiation and growth within nbMNs. Subsequently, D6-Com target genes were characteristic of neuronal maturation, including neuron projection and migration, synaptogenesis, dendritogenesis, cell junction and locomotory behavior. These results indicate that Kdm6b’s dynamic genomic recruitment is elaborately positioned to regulate both core cellular functions and stage-specific programs governing MN development and maturation.

### Kdm6b dynamically coordinates genomic landscapes of histone modifications

**Figure EV3:**
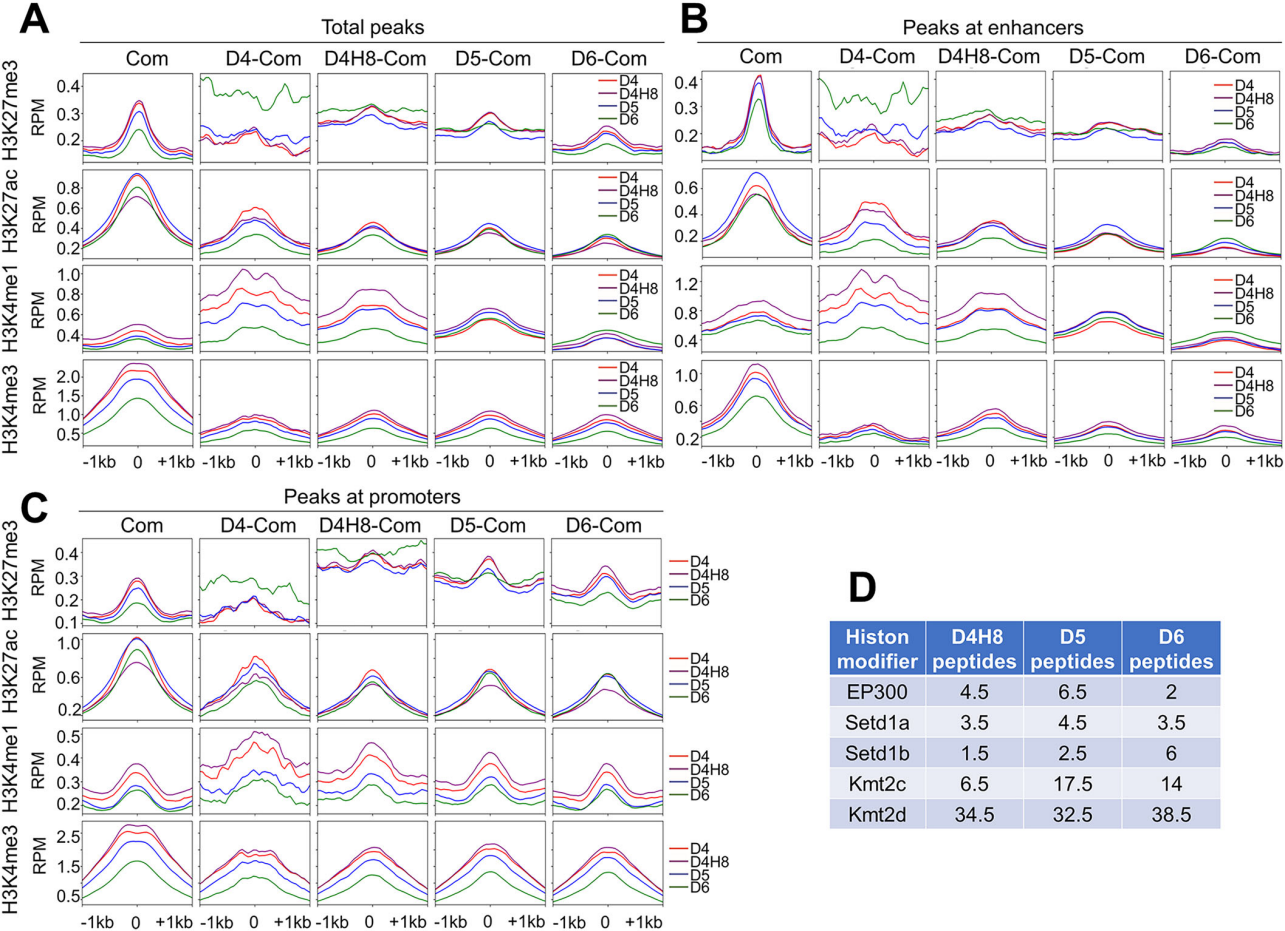
Kdm6b enrichment influences local chromatin modifications, related to Fig. 3. (**A**) Line plot profiles showing histone modification dynamics within Kdm6b common (Com) and stage-specific binding peaks (D4-Com, D4H8-Com, D5-Com, and D6-Com) across differentiation stages (D4, D4H8, D5, and D6). At stage-specific binding peaks, Kdm6b recruiting was closely associated with low H3K27me3 but high H3K27ac and H3K4me1 levels, while dismantling was largely related to high H3K27me3 but low H3K27ac and H3K4me1 levels. RPM indicates enrichment intensity. (**B**, **C**) Histon modification dynamics differ between the enhancer areas (**B**) and promoter regions (**C**) of Kdm6b stage-specific binding peaks. H3K4me1 dynamics was restricted to enhancers, while H3K27ac dynamics present at both enhancers and promoters. RPM indicates enrichment intensity. (**D**) Average peptide numbers identified for histone modifiers from Kdm6b IP-MS at the indicated stages from two biologically independent experiments.

**Figure 3:**
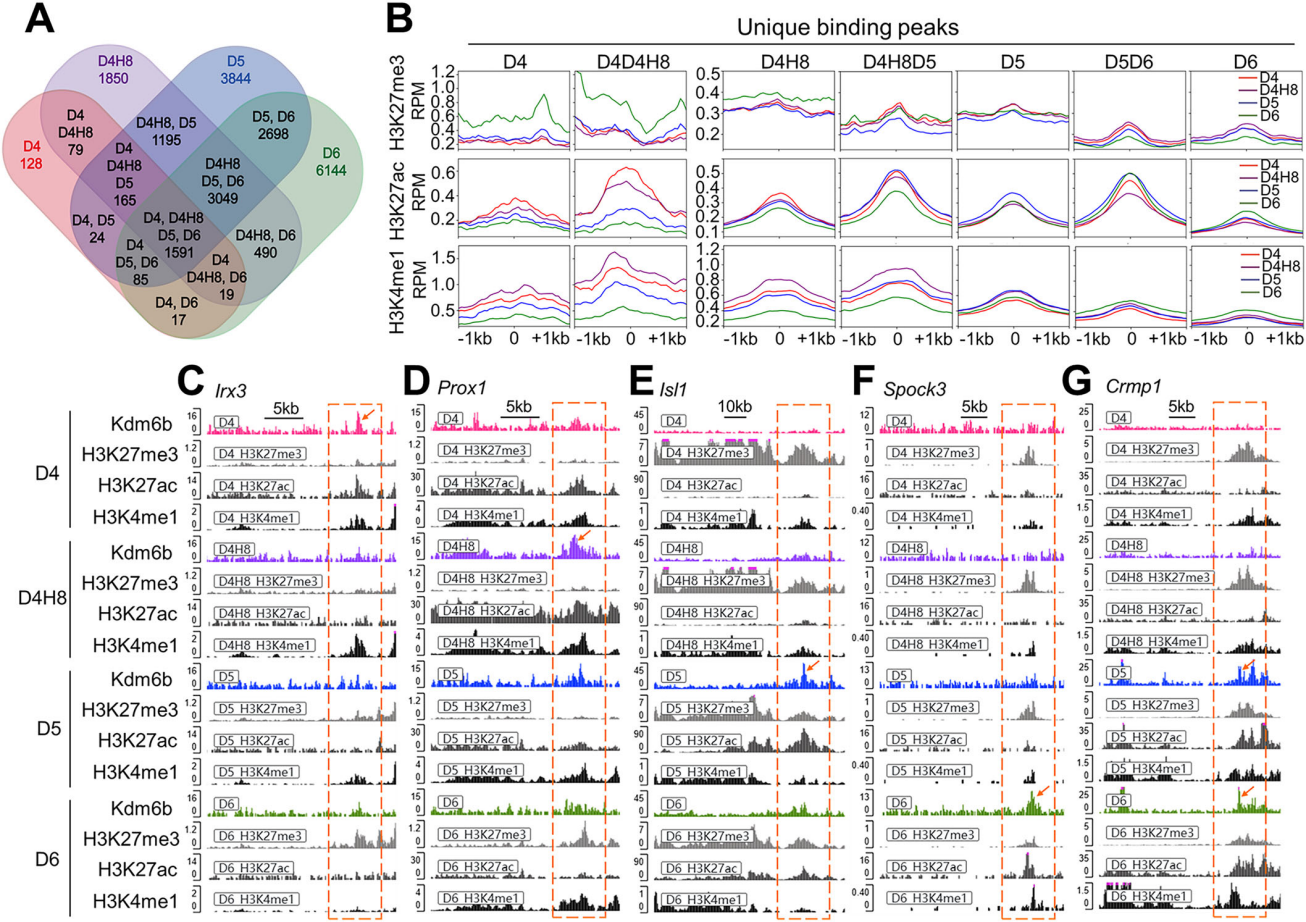
Kdm6b dynamically coordinates histone modification landscapes. (**A**) Venn Diagram showing temporally defined Kdm6b binding peaks: unique to each stage (D4, D4H8, D5, and D6), unique to two stages (D4D4H8, D4D5, D4D6, D4H8D5, D4H8D6, and D5D6), shared by three stages (D4D4H8D5, D4D4H8D5, D4D5D6, and D4H8D5D6) and shared by four stages (D4D4H8D5D6). Numbers denote respective binding sites. (**B**) Line plot profiles indicating histone modification dynamics within Kdm6b unique binding peaks (unique to each stage and two consecutive stages) across differentiation stages. Kdm6b binding associates with low H3K27me3 but high H3K27ac and H3K4me1 levels. RPM (Reads Per Million mapped reads) indicates enrichment intensity. (**C**–**G**) Genome browser tracks displaying Kdm6b occupancy and histone modification enrichment at representative genomic loci corresponding to genes *Irx3* (**C**), *Olig1* (**D**), *Isl1* (**E**), *Crmp1* (**F**), and *Spock3* (**G**) across MN developmental stages. RPM (reads per million mapped reads) indicates enrichment intensity. Source data are available online for this figure.

Given that Kdm6b acts as a histone demethylase, specifically removing the repressive mark H3K27me3, we investigated whether its enrichment influenced local chromatin modifications. Cut&Tag-seq analysis revealed that H3K27me3 levels were relatively low on stage-specific peaks at their respective stages of Kdm6b occupancy, but subsequently increased at distal stages (Fig. EV3A). Conversely, the active marks H3K27ac and H3K4me1 largely exhibited an inverse correlation with H3K27me3 at these sites, while H3K4me3 levels remained largely unchanged (Fig. EV3A). Moreover, enhancers and promoters differed in the nature of this inverse correlation, with H3K4me1 restricted to enhancers and H3K27ac present at both enhancers and promoters (Fig. EV3B,C). These results indicate that Kdm6b occupancy dynamically remodels the local histone modification landscape. To dissect these dynamics throughout MN differentiation, we focused on temporally defined Kdm6b binding peaks: unique to single stage (D4, D4H8, D5 and D6) or unique to two consecutive stages (D4D4H8, D4H8D5, and D5D6) (Fig. 3A). These peaks predominantly constituted the stage-specific binding events and most likely underlie the observed histone modification dynamics. As indicated, within unique binding peaks at early stages (D4_, D4D4H8_, and D4H8_unique peaks), Kdm6b occupancy correlated with very low H3K27me3 signal at both D4 and D4H8 stages (Fig. 3B). Following Kdm6b dissociation, H3K27me3 remained low at D5 but remarkably increased by D6 (Fig. 3B). In contrast, H3K27ac and H3K4me1 signals peaked at both D4 and D4H8 stages, then progressively decreased at D5, reaching the lowest levels at D6 (Fig. 3B). The early binding sites at genes *Irx3* and *Prox1* consistently confirmed this pattern (Fig. 3C,D). As differentiation proceeded, for unique binding peaks at intermediate stages (D4H8D5_ and D5_unique peaks), H3K27me3 reached its lowest level and inversely H3K27ac signals at D5, as shown by the binding sites at *Isl1* (Fig. 3B,E). H3K4me1 also remained elevated at both D4H8 and D5 stages (Fig. 3B). As the motor neuron was going toward maturity, Kdm6b unique binding peaks at late stages (D5D6_ and D6_unique peaks) displayed a higher H3K27me3 level at early stages, which decreased to its lowest level at D6 (Fig. 3B,F,G). Conversely, H3K27ac and H3K4me1 levels were low initially but increased at D6, as demonstrated by the binding sites at *Crmp1* and *Spock3* (Fig. 3B,F,G). Taken together, these results indicate a strong association between stage-specific genomic occupancy of Kdm6b and localized reduction of H3K27me3, concomitant with enrichment of H3K27ac and H3K4me1. In contrast, Kdm6b dissociation universally correlates with increased H3K27me3 and decreased H3K27ac and H3K4me1 levels.

**Figure EV4:**
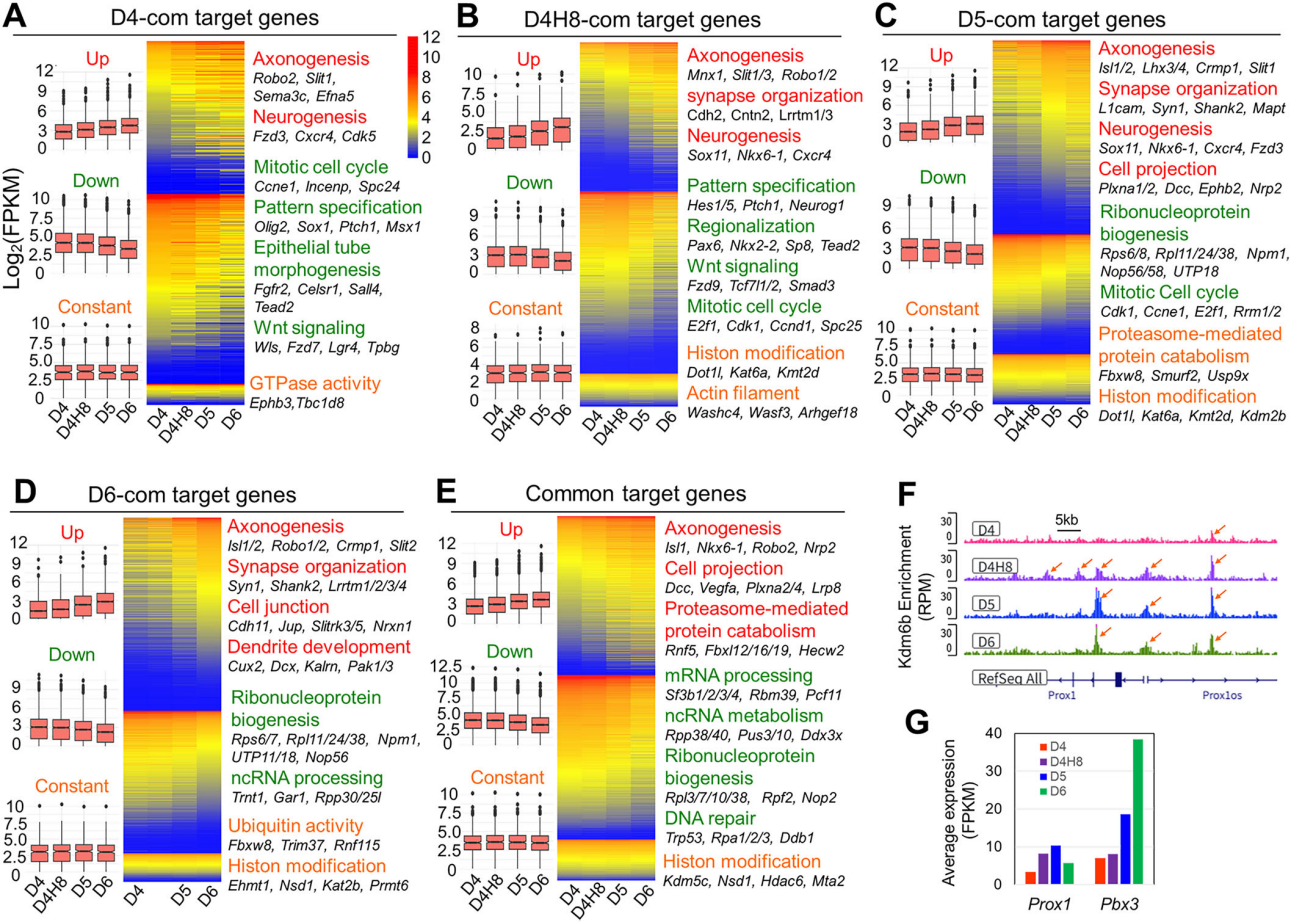
Kdm6b regulates stage-specific subsets of MN developmental genes, related to Fig. 4. (**A**–**E**) Temporal transcription profiles of Kdm6b stage-specific target genes, including D4-Com targets (**A**), D4H8-Com targets (**B**), D5-Com targets (**C**), D6-Com targets (**D**), and common target genes (**E**) during MN differentiation. Box plots and heatmaps depict gene expression profiles categorized as upregulated (Up, fold change ≥1.1), downregulated (Down, ≤0.9) and constant expression (Constant, 0.9 <fold change <1.1), as determined by pairwise comparison in the method section. Each subset underwent GO enrichment analysis, and top nonredundant terms and representative contributing genes are shown. FPKM (fragments per kilobase of transcript per million mapped fragments) was employed to indicate relative gene expression from different samples. (**F**) Genome browser tracks showing Kdm6b utilized both common and stage-specific binding modalities at the Prox1 locus. (**G**) Average mRNA levels of *Prox1* and *Pbx3* from RNA-seq datasets with three biologically independent experiments at each differentiation stage. FPKM was used to indicate relative gene expression from different samples.

These findings underscore the critical synergy between multiple histone modifications in modulating chromatin states in response to Kdm6b recruitment and dissociation. This synergistic action was further supported by IP-MS results demonstrating that Kdm6b co-immunoprecipitated with the H3K27 acetyltransferase EP300 and H3K4 methyltransferases Setd1a/1b and Kmt2c/2 d (Fig. EV4C). This suggests that Kdm6b functions not only as an H3K27 demethylase but also as a cofactor coordinating broader histone modification networks. Collectively, Kdm6b is a critical coordinator of chromatin landscapes, dynamically modulating the adaptable genomic distribution of histone modifications throughout MN differentiation.

### Kdm6b regulates developmental genes critical for stepwise MN differentiation

As Kdm6b dynamically binds to stage-specific genomic loci and remodels chromatin architecture critical for gene expression, we sought to identify Kdm6b-regulated genes. RNA-seq analysis across MN developmental stages (D4, D4H8, D5 and D6) revealed distinct transcriptional profiles, particularly between distal stages, indicative of temporally controlled gene expression (Appendix Fig. S1). As analyzed above, Kdm6b target genes comprised both stage-specific and common subsets, with the former exhibiting sequential correlations to MN developmental progression (Figs. 2G and EV2F). We therefore first characterized the overview of stage-specific target gene expression profiles. Based on their temporal expression patterns, Kdm6b target genes were categorized into three subsets: upregulation, downregulation or constant expression. Early stage-specific targets at D4 and D4H8 (D4-Com and D4H8-Com targets) were chiefly downregulated as differentiation progressed, including key developmental genes (e.g., *Olig2*, *Hes1*/*5*, *Ptch1*, *Cdk1*, *Neurog1*, and *Smad3*) (Fig. EV4A,B). They were highly expressed at early stages and functionally linked to early MN development at pMN and pnMN. In contrast to early targets, later stage-specific target genes of Kdm6b at D5 and D6 (D5-Com and D6-Com targets) were largely upregulated from low basal levels at earlier stages (Fig. EV4C,D). This subset encompassed a variety of essential MN genes (e.g., *Isl1/2*, *Lhx3/4*, *Mnx1*, *Crmp1*, *Robo2*, *Lrrtm3*, *Dcc*, *Nrp2*, *Cdh11*, *Slitrk3*, and *Cux2*), broadly involved in diverse neuronal differentiation and maturation processes. This finding demonstrated that the majority of Kdm6b target genes were stage-specifically regulated, coupling their expressions to distinct phases of MN development, differentiation and maturation.

Compared to stage-specific target genes, common Kdm6b target genes displayed higher average expression levels (Log_2_FPKM: 3.58 for common target genes vs. 2.80, 2.78, 2.71, and 2.53 for D4-Com, D4H8-Com, D5-Com and D6-Com targets, respectively) (Fig. EV4E), echoing constitutive binding of Kdm6b throughout differentiation. Notably, a fraction of key MN genes (e.g., *Pbx3* in Fig. EV2A and *Prox1* in Fig. EV4F) were subject to dual regulation through both common and stage-specific binding sites. Interestingly, their expression levels were well correlated with respective binding patterns (Fig. EV4G), unveiling the utilization of multiple binding modalities for exquisite expression control.

**Figure 4:**
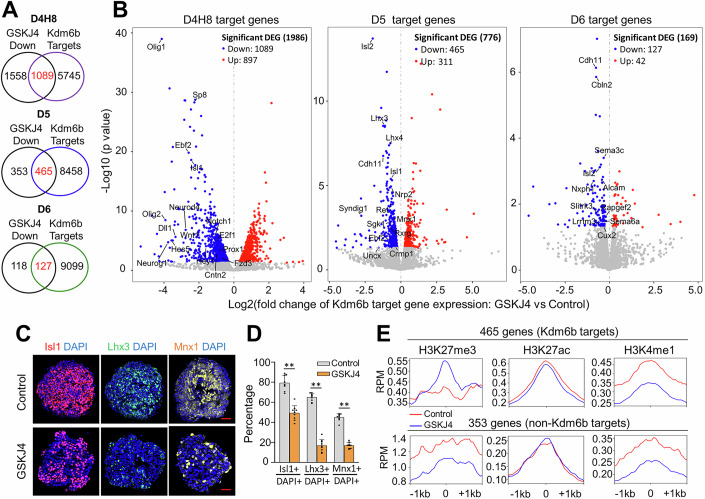
Inhibiting Kdm6b impairs the temporally ordered expression of developmental MN genes. (**A**) Comparison of Kdm6b target genes with downregulated genes by GSKJ4 treatment at the indicated stages. The number of common genes is marked in red. (**B**) Volcano plots displaying significant DEGs (*p* value <0.05) upon GSKJ4 treatment relative to control at the indicated stages. Blue dots: downregulated DEGs (log_2_FC <0, *p* < 0.05), with representative genes shown; red dots: upregulated DEGs (log_2_FC >0, *p* < 0.05). *P* value was determined by Wilcox rank-sum test. (**C**, **D**) Immunostaining of Isl1, Lhx3, and Mnx1 within D5 nbMNs treated with GSKJ5 for one day (**C**) and quantification of immunostaining results (**D**). The data shows the percentage of positive cells relative to all DAPI cells, plotted as mean ± SD. At least eight neurospheres were unbiasedly selected for quantification from one representative experiment. **p < 0.01 (one-way ANOVA test; from left to right: 1.8e-7, 1.0e-12, and 4.1e-12). Scale bars: 50 μm. (**E**) Enrichment of H3K27me3, H3K27ac, and H3K4me1 within D5 nbMNs treated with GSKJ4 treatment (blue line) relative to control (red line) at 465 Kdm6b target genes and 353 non-Kdm6b target genes, as shown in (**A**). RPM indicates enrichment intensity. Source data are available online for this figure.

To further functionally interrogate the role of Kdm6 in regulating its target gene expression, GSKJ4 was employed to specifically inhibit Kdm6b enzyme activity at specific timepoints. RNA-seq analysis of genes downregulated upon GSKJ4 treatment at each stage revealed a substantial enrichment of Kdm6b target genes, confirming the on-target effect of this Kdm6b inhibitor (Fig. 4A). When focusing on Kdm6b target genes, we identified 1986, 776, and 169 DEGs significantly transcriptionally altered by GSKJ4 treatment at D4H8, D5 and D6 respectively (Fig. 4B). Importantly, a majority of DEGs were downregulated (54.8% at D4H8, 59.9% at D5 and 75.1% at D6) (Fig. 4B), including key MN developmental genes such as *Olig2* and *Neurog1* at D4H8, *Isl1* and *Mnx1* at D5, *Cdh11* and *Cux2* at D6. Strikingly, these downregulated genes were directly implicated in stepwise MN development and differentiation (Appendix Fig. S2). Additionally, GSKJ4 treatment significantly reduced the number of cells transitioning to the next developmental stage D5 nbMN from D4 pMN (Fig. 4C,D). To identify how histone modifications are remodeled by GSKJ4 to regulate gene downregulation, Cut&Tag-seq was employed to investigate chromatin landscapes within D5 nbMNs. The result showed that GSKJ4 treatment resulted in increased levels of H3K27me3 at 465 Kdm6b target genes, whereas no such increase was observed at 353 non-Kdm6b target loci (Fig. 4E). These findings further support the role of GSKJ4 as a specific inhibitor of Kdm6b demethylase activity and suggest an indirect effect on non-target genes. Notably, GSKJ4 dramatically reduced H3K4me1 levels, although it had a minimal effect on H3K27ac (Fig. 4E), contrasting with the decreased H3K27ac observed upon natural Kdm6b dissociation (Fig. 3B). This further suggests that the role of Kdm6b in coordinating histone modifications is partially inhibited by GSKJ4.

Collectively, these findings demonstrate that Kdm6b directs the sequential activation of temporally regulated genes, thus enabling stepwise MN differentiation.

### Kdm6b sequentially associates with stage-specific transcription factors

**Figure 5:**
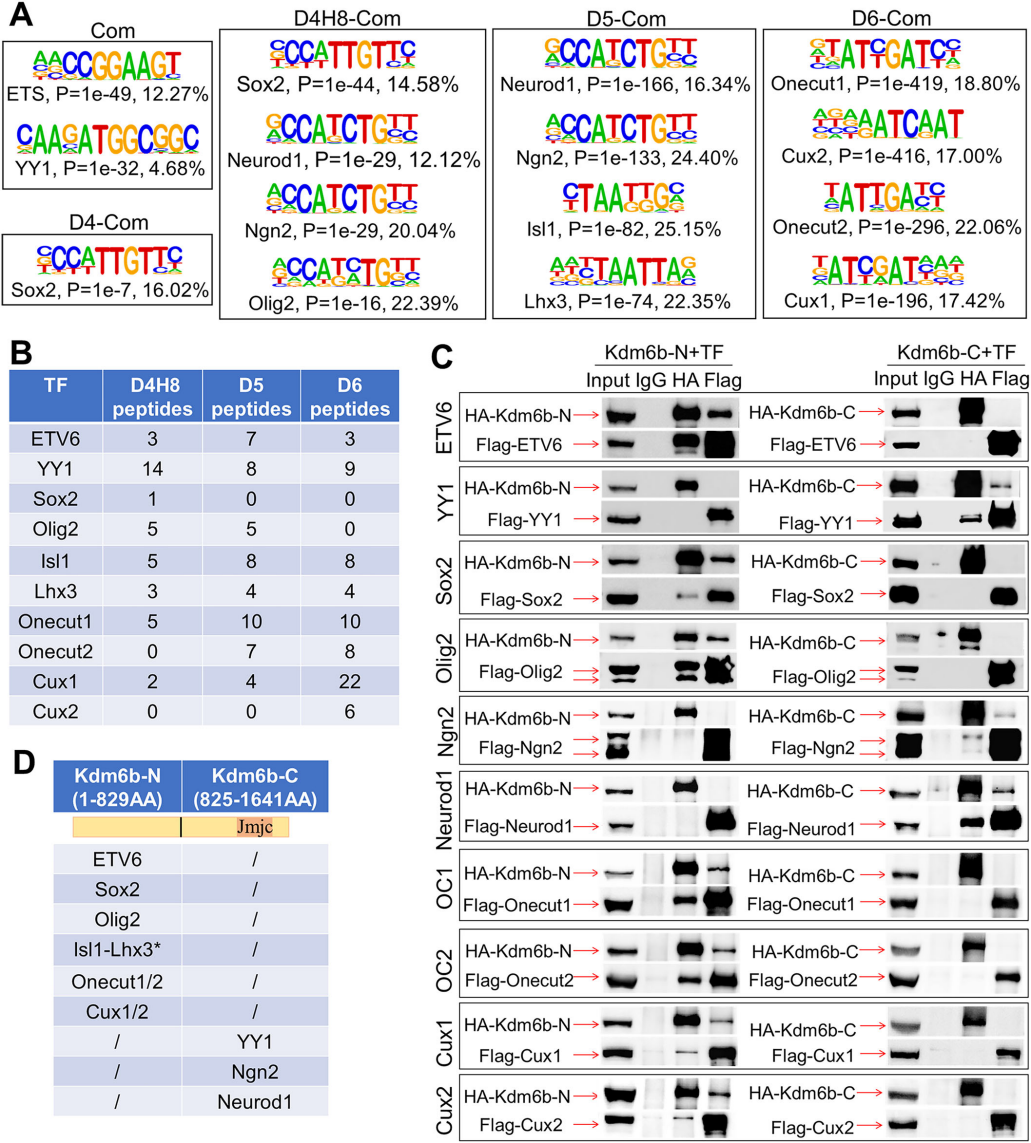
Kdm6b stage-specifically partners with developmental TFs throughout MN development. (**A**) Motif enrichment analyses of common (Com) and stage-specific Kdm6b binding peaks (D4-Com, D4H8-Com, D5-Com, and D6-Com). *P* value and % of target sequences with each motif are shown, respectively. (**B**) Kdm6b-interacting transcription factors identified by IP-MS at the indicated stages. The peptide number, corresponding to each TF, was identified from a single experiment (*n* = 1). (**C**) Co-IP assays with IgG, HA and Flag antibodies within HEK293 cells transfected with HA-Kdm6b-N or C terminus and Flag-TFs, followed by western blotting. (**D**) Distinct domains of Kdm6b associating with different sets of TFs, based on (**C**) and previous findings (asterisk). Source data are available online for this figure.

**Figure EV5:**
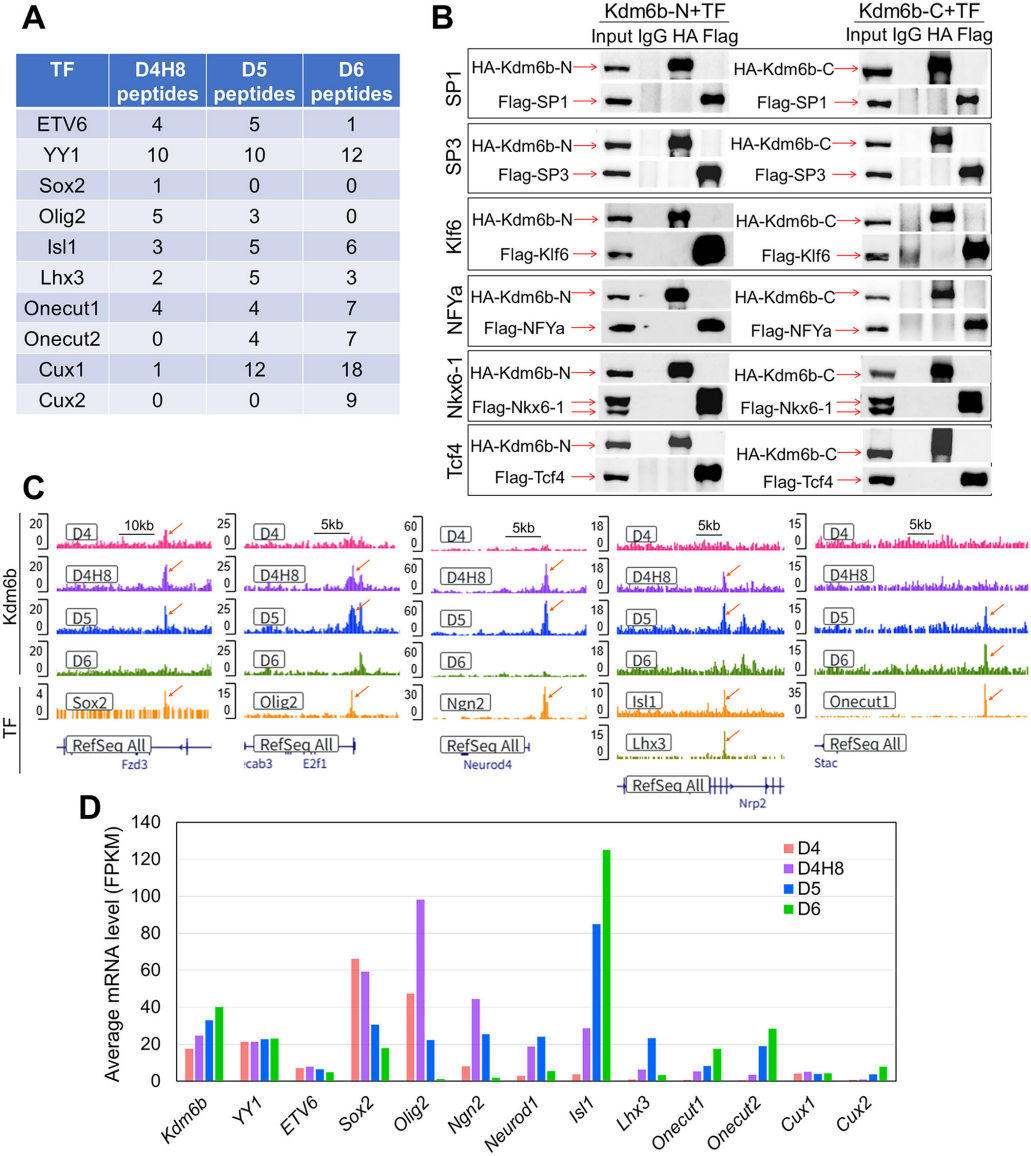
Kdm6b associates with temporal TFs throughout MN development, related to Fig. 5. (**A**) Kdm6b-interacting transcription factors identified by IP-MS at the indicated stages. The peptide number, corresponding to each TF, was identified from the second experiment (*n* = 1). (**B**) Co-IP assays with IgG, HA and Flag antibodies within HEK293 cells transfected with HA-Kdm6b-N or C terminus and Flag-TFs, followed by western blotting. The results indicated no interaction between HA-Kdm6b-N or C terminus and tested Flag-TFs. (**C**) Genome browser tracks exhibiting co-enrichment of Kdm6b and developmental TFs at representative genomic loci. (**D**) Average mRNA levels of *Kdm6b* and developmental TFs from RNA-seq datasets at each differentiation stage (*n* = 3 biological replicates). FPKM is used to indicate relative gene expression from different samples.

As an epigenetic cofactor, Kdm6b interacts with TFs to regulate gene expression. To identify potential TFs recruiting Kdm6b to its genomic loci, we performed TF motif enrichment analyses. Analysis of common peaks revealed enrichment for motifs corresponding to TFs such as Krüppel-like factors (Klfs), specificity proteins (Sps), NFY, E26 transformation-specific (ETS) factors and YY1 (Fig. 5A; Dataset EV1). To experimentally identify Kdm6b-associated TFs, we performed Kdm6b immunoprecipitation coupled with mass spectrometry (IP-MS) in stage-specific MNs. Notably, while top predicted Klfs and Sps were not consistently detected, ETV6 (an ETS factor) and YY1 were found to associate with Kdm6b across differentiation stages (Fig. 5B). Furthermore, co-immunoprecipitation (co-IP) with overexpression experiments confirmed that ETV6 and YY1, but not Klf6, Sp1, Sp3, or NFYa, interacted with Kdm6b (Figs. 5C and EV5A). These findings unravel that ETV6 and YY1 are constitutively associated with Kdm6b throughout MN differentiation, potentially regulating fundamental cellular processes as suggested by GO analysis (Fig. EV2F) and previous reports (Latorre et al, 2019; Zurkirchen et al, 2019). In contrast to common peaks, stage-specific peaks presented distinct TF motif enrichment at each developmental stage. At D4 and D4H8, Sox family TF motifs were highly ranked (Fig. 5A and Dataset EV1). Sox2 was also identified in the IP-MS dataset at D4H8 (Fig. 5B), supporting its role as a key Kdm6b-associating TF within pMNs. Interestingly, motif analysis and IP-MS at D4H8 also identified Olig2 as an associated TF (Fig. 5A,B; Dataset EV1), implying their coordination in pMN identity specification. Within D5 nbMNs, both motif enrichment and IP-MS results indicated that Isl1 and Lhx3 associated with Kdm6b (Fig. 5A,B; Dataset EV1), corroborating our previous finding (Wang et al, 2022). Within D6 HMC mMNs, motif analysis identified Onecut1/2 and Cux1/2 as the top-ranked TFs, and IP-MS confirmed their robust association with Kdm6b (Fig. 5A,B; Dataset EV1). However, within MMC cells, Onecut1/2 and Cux1/2 motifs were much lower-ranked (Dataset EV1, Onecut1 ranked #38 and others lower), suggesting Kdm6b preferentially associated with distinct TF sets in different mMN subtypes. At D6, both motif analysis and IP-MS also identified Isl1 and Lhx3 as Kdm6b-associating TFs (Fig. 5A,B; Dataset EV1), consistent with sustained high Isl1 expression and residual low Lhx3 level in the in vitro MN differentiation system (Fig. 1B). To further validate these interactions, we performed co-IP following overexpression of Sox2, Olig2, Onecut1/2, and Cux1/2 with Kdm6b. These experiments confirmed Kdm6b association with each of these TFs (Fig. 5C). Of particular note, co-IP also revealed that Ngn2 and Neurod1 associated with Kdm6b while Nkx6-1 and Tcf4 did not (Figs. 5C and EV5A). Ngn2 and Neurod1 motifs were found highly enriched within both D4H8 and D5 peaks (Fig. 5A; Dataset EV1), although these two TFs were not detected in IP-MS. Additionally, co-IP results indicated that Kdm6b utilized different domains to interact with distinct developmental TFs (Fig. 5D), hinting at its structural and functional diversity in regulating MN development. Furthermore, constitutively (YY1 and ETV6) and stage-specifically (Sox2, Olig2, Ngn2, Neurod1, Isl1, Lhx3, Onecut1/2, and Cux1/2) associating TFs essentially coordinated their expression profiles with temporal partnerships with Kdm6b (Fig. EV5B).

**Figure 6:**
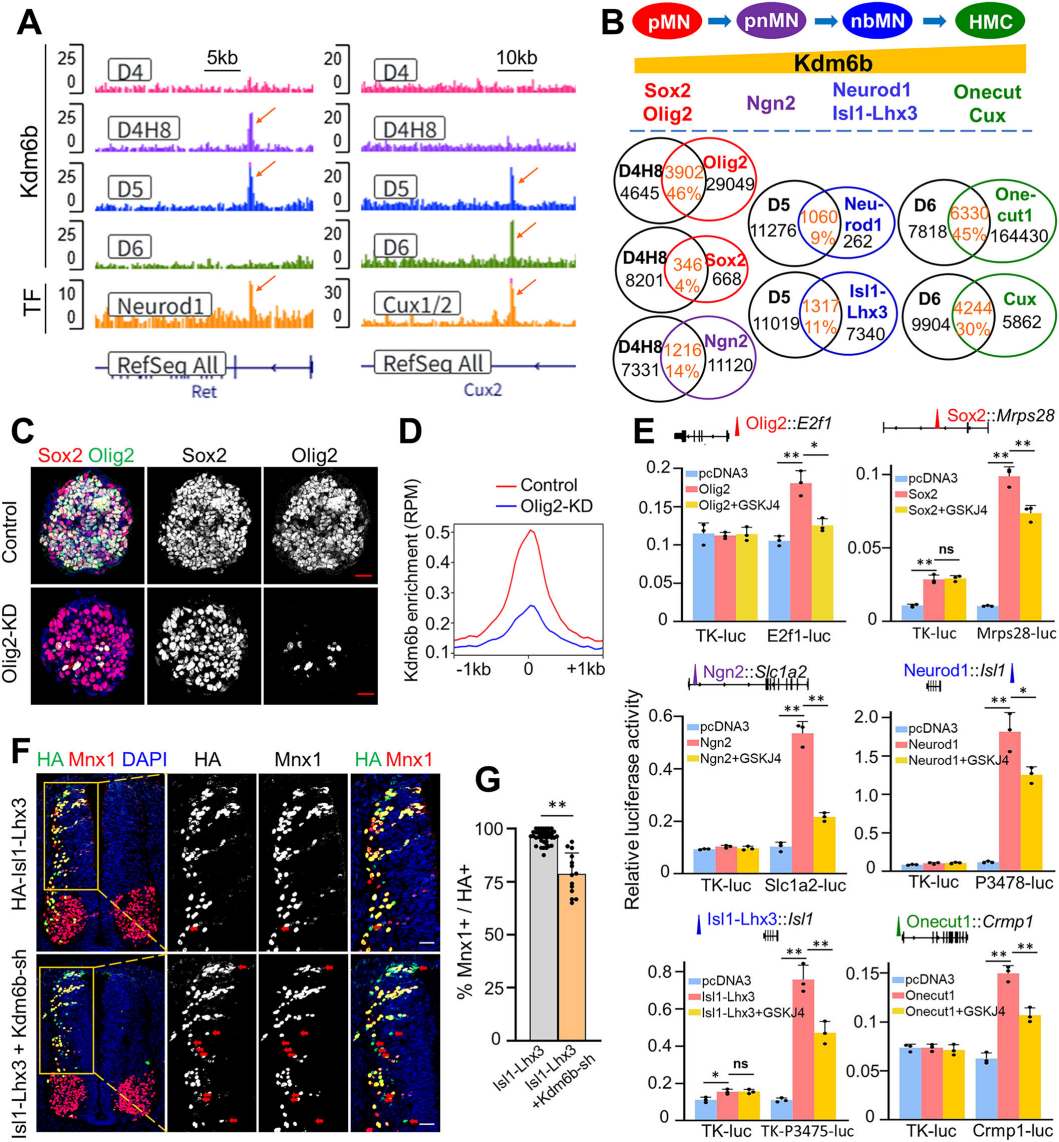
Kdm6b is essential for transcriptionally regulating its co-target genes with temporally associated TFs. (**A**) Genome browser tracks showing co-enrichment of Kdm6b and Neurod1 or Cux1 at representative genomic loci. (**B**) Schematic illustrating Kdm6b association with stage-specific TFs across MN differentiation and their extensive co-occupancy at shared sites (central overlapping regions; Kdm6b in black font, TF in colored font). Numbers indicate binding sites, and percentages represent the proportion of overlapping sites relative to total Kdm6b binding sites at the indicated stages. (**C**) Olig2 and Sox2 immunostaining within control and Olig2 knockdown (KD) cells at D4H8. Scale bars: 50 μm. (**D**) Kdm6b enrichment within control and Olig2 knockdown (KD) cells at D4H8. RPM indicates enrichment intensity. (**E**) Luciferase reporter assays showing TF-induced activation of their respective sites co-bound by Kdm6b. GSKJ4 partially attenuated this activation. Olig2::*E2f1* indicates the Olig2 binding sites (colored peak) within the *E2f1* locus, and the same principle applies to others. Representative data were presented as mean ± SD. *P* value was determined by one-way ANOVA test (*n* = 3 technical replicates). ns nonsignificant; *p < 0.05; **p < 0.01 (significant *p* values from left to right for Olig2: 0.008, 0.01; for Sox2: 0.005, 0.002, 0.007; for Ngn2: 0.001, 0.003; for Neurod1: 0.007, 0.04; for Isl1-Lhx3: 0.02, 0.004, 0.008; for Onecut1: 0.0002, 0.002). (**F**, **G**) Immunostaining of HA and Mnx1 in chicken spinal cords electroporated with HA-Isl1-Lhx3 or HA-Isl1-Lhx3 + Kdm6b-shRNA (**F**) and quantification of immunostaining results (**G**). At least three embryos and three slices per embryo were used for quantification. Data were presented as mean ± SD. **p < 0.01 (6e-14, one-way ANOVA test). Source data are available online for this figure.

To further illuminate genome-wide co-occupancy profiles of Kdm6b and developmental TFs, we mapped their overlapping binding across genomic loci. Cut&Run-seq of Neurod1 in D5 nbMNs revealed that 1060 out of 1322 Neurod1 binding peaks (80.2%) overlapped with Kdm6b peaks, strongly supporting their co-recruitment (Fig. 6A,B). Likewise, Cut&Run-seq of Cux1/2 at D6 identified 4244 peaks co-bound by Cux1/2 and Kdm6b (Fig. 6A,B). Concomitantly, we analyzed published ChIP-seq datasets: Sox2 in neural progenitors (Bergsland et al, 2011), Olig2 in pMNs (Mazzoni et al, 2011), Ngn2 in pnMNs and Isl1-Lhx3 in nbMNs (Velasco et al, 2017), and Onecut1 in mMNs (Rhee et al, 2016). The overlapping analyses identified 346, 3902, 1216, 1317, and 6330 peaks co-enriched by Sox2, Olig2, Ngn2, Isl1-Lhx3 and Onecut1 with Kdm6b, respectively (Figs. 6B and EV3C). The genes associated with co-enriched and non-co-enriched sites between Kdm6b and TFs are involved in distinct biological processes, suggesting specific roles of Kdm6b-TF partnerships in regulating MN development (Dataset EV2). Moreover, knockdown of Olig2 remarkably attenuated Kdm6b recruitment on the co-targets by Kdm6b and Olig2 (Fig. 6C,D), demonstrating the Kdm6b binding is dependent on the presence of its associating TFs. Together, these results suggest that Kdm6b binding specificity is dictated by its interaction with stage-specific TFs, which in turn facilitates the precise temporal regulation of MN differentiation (Fig. 6B).

### Kdm6b is essential for TF-mediated temporal transcriptional regulation

Since temporal TFs direct Kdm6b recruitment on the genome, we wonder if target genes by TFs are regulated by Kdm6b. Indeed, we found that many of DEGs downregulated by GSKJ4 treatment were co-targets of Kdm6b and its temporally associating TFs (Fig. 4B). At D4H8, among 1089 downregulated DEGs, 602 genes (e.g., *Olig1/2*, *E2f1*, *Hes5*, and *Prox1*) were co-targets of Kdm6b and Olig2, 57 genes (e.g., *Olig2*, *Wnt1*, *Fzd3*, and *Notch1*) were co-targets of Kdm6b and Sox2, and 205 genes (e.g., *Neurog1*, *Neurod4*, *Ebf2*, *Isl1*, and *Mnx1*) were co-targets of Kdm6b and Ngn2 (Dataset EV3). These downregulated co-targets were centrally involved in early MN development, including Wnt/Notch signaling, cell proliferation and cell cycle transition, epithelial tube morphogenesis, pattern specification, regionalization, neurogenesis and axonogenesis (Appendix Fig. S3A–C). Similarly, among 465 downregulated DEGs at D5, 61 genes (e.g., *Isl1*, *Lhx3*, *Mnx1*, *Ebf2*, *Sgk1*, and *Ret*) were co-targets of Kdm6b and Neurod1, and 81 genes (e.g., *Isl1/2*, *Lhx4*, *Mnx1*, *Ebf2*, *Nrp2*, *Uncx*, *Crmp1*, *Cdh11*, and *Rxrg*) were co-targets of Kdm6b and Isl1-Lhx3 complex (Dataset EV4). These co-targets were mainly associated with pattern specification, regionalization, cell fate commitment, spinal cord development, neurogenesis, axonogenesis, axon guidance, neuron projection and developmental growth (Appendix Fig. S3D, E). Furthermore, among 127 downregulated DEGs at D6, 86 genes (e.g., *Isl2*, *Cdh11*, *Cux2*, *Cbln2*, *Lrrtm3*, *Slitrk3*, *Rapgef2*, and *Sema6a*) were identified as co-targets of Kdm6b and Onecut1, and 42 genes (e.g., *Cdh11*, *Cux2*, *Rapgef2*, *Cbln2*, *Alcam*, *Nxph1*, and *Sema3c*) as co-targets of Kdm6b and Cux1/2 (Dataset EV5). These downregulated co-targets within mMNs were highly enriched in MN maturation processes, including cell junction, synapse organization and assembly, axonogenesis and axon guidance and dendrite development (Appendix Fig. S3F,G). Thereby, these findings indicate that Kdm6b is essential for transcriptionally regulating the key developmental genes co-bound by both Kdm6b and temporal TFs.

To assess the functional significance of Kdm6b in gene activation at the binding site level, we cloned genomic fragments co-recruited by Kdm6b and key developmental TFs into luciferase reporter constructs. The results revealed that each TF (Olig2, Sox2, Ngn2, Neurod1, Isl1-Lhx3, and Onecut1) significantly activated its cognate reporter construct (Fig. 6E). Crucially, this activation was significantly attenuated upon GSKJ4 inhibition of endogenous Kdm6b activity, indicating that Kdm6b function was required for full transcriptional activation at these co-bound sites (Fig. 6E). In parallel, in vivo electroporation of Isl1-Lhx3 complex into developing chicken spinal cords resulted in activation of its target gene *Mnx1*, while this induction was weakened by Kdm6b knockdown (Fig. 6F,G). Together, these findings support a model in which Kdm6b acts with temporal TFs to precisely regulate their co-targeted MN genes and drive differentiation.

## Discussion

**Figure 7:**
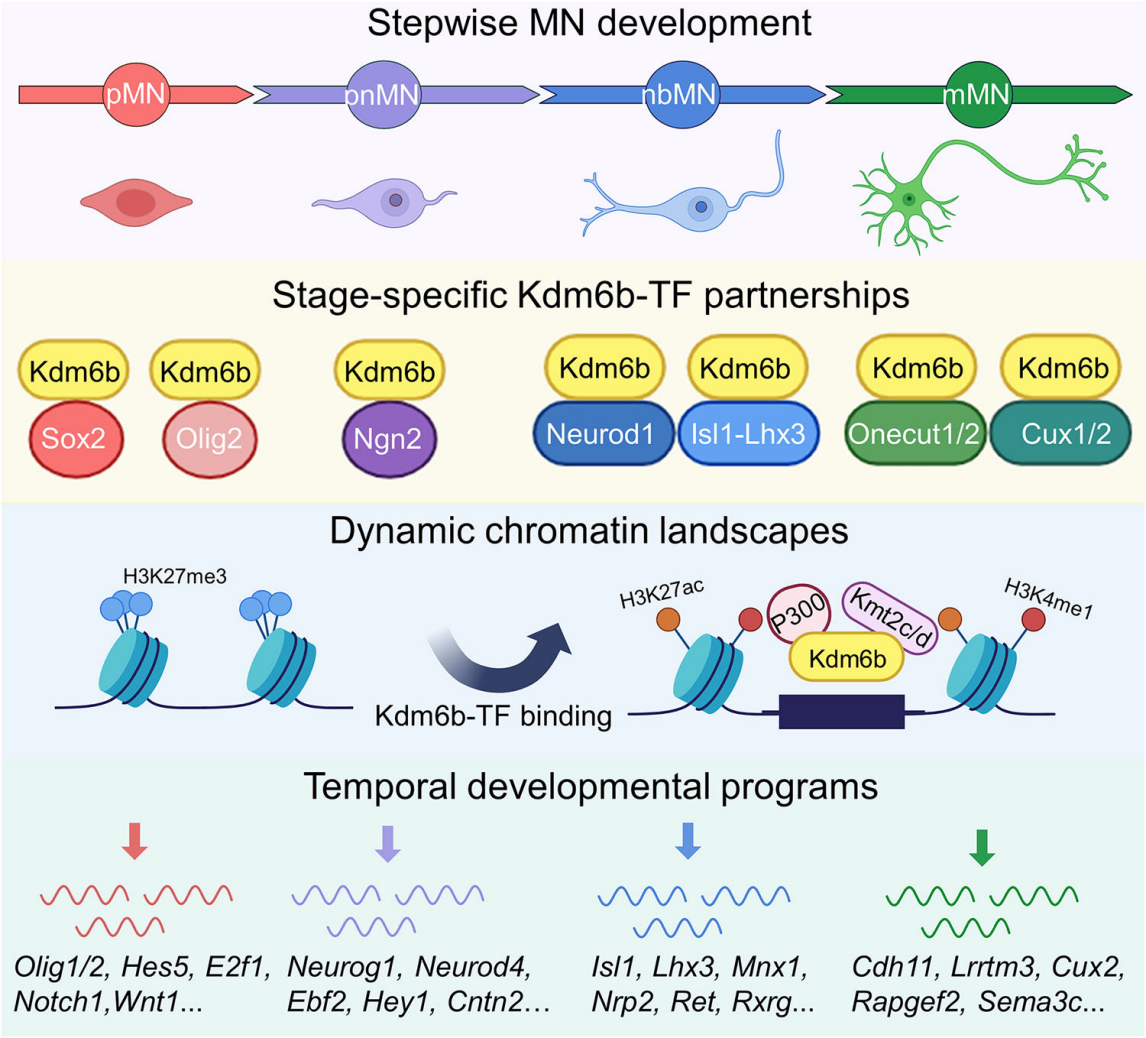
Schematic representation of Kdm6b as an epigenetic regulator to coordinate stepwise MN development. By sequentially partnering with stage-specific TFs and dynamically remodeling chromatin landscapes, Kdm6b precisely regulates temporal MN gene expression. The model was created with BioRender.com.

Histone modifications establish a sophisticated layer of epigenetic regulatory network by modulating chromatin structure and function, and play indispensable roles in cell development and differentiation (Morgan and Shilatifard, 2020; Podobinska et al, 2017; Yagi et al, 2025). These processes entail spatiotemporal precision to safeguard cell fate commitment and against developmental abnormalities. This study elucidates the dynamic epigenetic coordination by which the histone demethylase Kdm6b governs stepwise MN differentiation. By integrating genome-wide binding profiling, histone modification mapping, functional perturbation and TF cooperativity analysis, we demonstrate that Kdm6b acts as a master epigenetic conductor, coordinating with stage-specific developmental TFs and chromatin modifications to ensure precise gene activation and MN development progression (Fig. 7). Our findings resolve a longstanding question about how an epigenetic regulator achieves temporal precision in directing cellular differentiation and offer three transformative insights into MN development.

First, Kdm6b-TF partnerships define stage-specific regulatory logic. Kdm6b does not operate in isolation but sequentially forms stage-specific complexes with key developmental TFs: Sox2/Olig2 in proliferating pMNs, Ngn2 in lineage-committing pnMNs, Neurod1 and Isl1-Lhx3 in post-mitotic nbMNs, Onecut1/2 and Cux1/2 in subtype-specifying mMNs. This epigenetic partnering explains how Kdm6b achieves locus selectivity as TFs direct its recruitment to distinct genomic sites, enabling stage-appropriate gene activation. The co-target genes were sequentially associated with stepwise MN development, progressively aligning with the driving roles of stage-specific TFs. Crucially, disruption of these partnerships (e.g., Kdm6b inhibition or knockdown) ablated target gene expression, confirming their functional necessity. This TF-cofactor collaboration represents a fundamental mechanism for canalizing temporally precise epigenetic regulation toward unidirectional cell lineage commitment and stepwise differentiation.

Within this framework, several aspects merit additional attention. We designated proneural TF Ngn2-expressing MNs as pnMNs to distinguish this transient stage, accentuating its significance in MN development (Lee et al, 2005; Velasco et al, 2017). As Ngn2 is immediately downregulated in post-mitotic MNs, the expression of another proneural TF, Neurod1, peaks in nbMNs but decreases in mMNs, making it an ideal marker for nbMNs. The sequential roles of proneural TFs Ngn2 and Neurod1 reveal a transcriptional cascade propelling neurogenesis from pMN to pnMN and nbMN. This transiency likely contributes to the weak and instantaneous nature of their interactions with Kdm6b, potentially explaining their absence in IP-MS due to its limitation (Dunham et al, 2012). However, their interactions were confirmed upon robust overexpression and by extensive genomic co-occupancy. Additionally, we identified the association between Kdm6b and Cux1/2, with their co-target genes extensively implicated in MN differentiation and maturation, further broadening knowledge beyond previous reports on neuronal function of Cux1/2 (Cubelos et al, 2010; Weiss and Nieto, 2019). Notably, Kdm6b also constitutively associated with YY1 and ETV6 at all stages, indicating multilayered regulatory partnerships, while their co-targets and functional significance require future investigation. Furthermore, this histone modifier-TF partnerships within spatially distinct mMN subtypes, such as LMC and PGC, necessitate further exploration.

Second, Kdm6b remodels histone modification dynamics with a tripartite Code. The combinatorial landscape of histone modifications orchestrates spatially defined chromatin topologies and diversifies transcriptional outcomes. This study reveals Kdm6b recruitment coordinates a histone modification cascade: localized H3K27me3 reduction is accompanied by rapid H3K27ac deposition and H3K4me1 enrichment (H3K27me3↓/H3K27ac↑/H3K4me1 ↑ ). This chromatin configuration establishes an activation-permissive state for timed gene transcription. The stage-specific removal of H3K27me3 triggered by Kdm6b occupancy also provides an alternative mechanism underlying sequential unfolding of epigenetic barriers set for neuronal maturation (Ciceri et al, 2024). Conversely, Kdm6b dissociation coincides with a decrease of H3K27ac/H3K4me1 and restoration of H3K27me3, albeit some delay (H3K27me3↑/H3K27ac↓/H3K4me1↓), thus establishing a repressive state for timely gene silencing. The slow kinetics of H3K27me3 reestablishment likely reflect transcriptional memory and cell plasticity during the intermediate cell states (Xiong and Zhu, 2025). IP-MS further confirmed the physical association of Kdm6b with H3K27 acetyltransferase EP300 and H3K4 methyltransferases (Setd1a/1b and Kmt2c/2 d), indicating a synergistic machinery of multiple histone modifiers. Kdm6b predominantly associates with Kmt2c (Mll3) and Kmt2d (Mll4) (Fig. EV3D), which are responsible for implementing H3K4me1 other than H3K4me3 (Hu et al, 2013), further illuminating that H3K4me1-enriched enhancers are major regulatory regions utilized by Kdm6b. This is further supported by the inverse correlation between H3K4me1 and H3K27me3 at only enhancer areas (Fig. EV3B). Additionally, GSKJ4 treatment did not substantially affect H3K27ac, unlike Kdm6b dissociation. These findings suggest that Kdm6b likely functions as a scaffold to interact with other coregulators to dynamically modulate H3K27ac/H3K4me1 via an enzyme-independent mechanism as previously suggested (Miller et al, 2010; Zhao et al, 2013). Investigating the non-enzymatic mechanism requires further detailed study. Collectively, this kinetic tripartite code ensures rapid chromatin state transitions aligned with differentiation progression. This study remains limited in addressing how chromatin environment conversely influences TF binding, as previously suggested (Bartke et al, 2010; Wang et al, 2024), thus prompting future research.

Third, the binding architecture of Kdm6b guides its functional diversification. Kdm6b’s binding topology on the genome evolves dynamically: early stages favor promoter-proximal sites regulating proliferation and pattern specification genes (e.g., *Olig2*, *Hes5*, *E2f1*, *Notch1*, *Wnt1*, and *Fzd3*), while maturation stages gain more sites toward distal enhancers controlling neuronal functionality genes (e.g., *Isl1/2*, *Crmp1*, *Slitrk3*, *Sema3c*, *Cdh11*, and *Cux2*). Inhibiting Kdm6b impaired stage-specific transcriptional profiles, further validating Kdm6b’s stage-resolved functions. Strikingly, the spatial redistribution parallels with increasing regulatory complexity, as mMNs deploy more binding sites per gene, enabling multifaceted regulation of maturation programs. Intriguingly, neuronal genes employ a similar regulatory strategy (longer regulatory distance and greater number of regulatory elements relative to non-neuronal genes) from the transition of invertebrates to vertebrates (Closser et al, 2022), suggesting Kdm6b may play a role in the evolution of neuronal diversity and complexity. Additionally, some genes critical for lineage progression (e.g., *Prox1* and *Pbx3*) employ dual regulatory strategies, combining constitutive and stage-specific binding patterns, further facilitating regulatory diversification.

In summary, our findings support a model where dynamic Kdm6b-TF partnerships and associated chromatin landscapes execute a temporally layered epigenetic program (Fig. 7). This temporal, precise control elucidates how a single epigenetic regulator orchestrates multistep differentiation. The dynamic epigenetic coordination has broad implications for neurodevelopment and diseases. Dysregulation of Kdm6b activity or Kdm6b-TF partnerships may underlie MN diseases and neurodevelopmental disorders, where Kdm6b mutations occur not only within the enzymatically active JmjC domain but also in other domains (Rots et al, 2023). Therapeutically, modulating Kdm6b-TF interactions could target stage-specific MN differentiation in regenerative contexts, as Kdm6b was involved in neuronal regeneration (Yang et al, 2025). Future work would explore how this paradigm extends to other cell lineages and whether similar epigenetic coordination operates during disease-associated reprogramming.

## Methods

**Table:** Reagents and tools table

Reagent/resource	Reference or source	Identifier or catalog number
**Experimental models**		
Mouse C57BL/6 ES cell	Oricell	MUBES-01001
HEK293 cell	ATCC	CRL-1573
P19 cell	Procell	CL-0179
Chick embryo	This paper	N/A
**Recombinant DNA**		
pcDNA3-HA-Kdm6b-N	This paper	N/A
pcDNA3-HA-Kdm6b-C	This paper	N/A
pcDNA3-Flag-Sox2	This paper	N/A
pcDNA3-Flag-Olig2	This paper	N/A
pcDNA3-Flag-Ngn2	This paper	N/A
pcDNA3-Flag-Neurod1	This paper	N/A
pcDNA3-Flag-Onecut1	This paper	N/A
pcDNA3-Flag-Onecut2	This paper	N/A
pcDNA3-Flag-Cux1	This paper	N/A
pcDNA3-Flag-Cux2	This paper	N/A
pcDNA3-Flag-YY1	This paper	N/A
pcDNA3-Flag-ETV6	This paper	N/A
pcDNA3-Flag-SP1	This paper	N/A
pcDNA3-Flag-SP3	This paper	N/A
pcDNA3-Flag-Klf6	This paper	N/A
pcDNA3-Flag-NFYa	This paper	N/A
pcDNA3-Flag-Nkx6-1	This paper	N/A
pcDNA3-Flag-Tcf4	This paper	N/A
TK-E2f1-luc	This paper	N/A
TK-Mrps28-luc	This paper	N/A
TK-Slc1a2-luc	This paper	N/A
TK-P3475-luc	This paper	N/A
TK-P3478-luc	This paper	N/A
TK-Crmp1-luc	This paper	N/A
pG-SUPER-Kmd6b-shRNA	(Estaras et al, 2012)	N/A
**Antibodies**		
Rabbit anti-Olig2	Proteintech	Cat#13999-1-Ap
Mouse anti-Ngn2	R&D	Cat# MAB3314
Rabbit anti-Neurod1	Cell Signal Technology	Cat# 62953
Mouse anti-Mnx1	DHSB	Cat# 81.5C10
Rabbit anti-Isl1	Abcam	Cat# ab20670
Mouse anti-Lhx3	DHSB	Cat# 67.4E12
Rabbit anti-Neurod1	Proteintech	Cat# 12081-1-AP
Rabbit anti-Cux1/2	Abcam	Cat# ab309139
Rabbit anti-Kdm6b	This paper	N/A
Rabbit anti-H3K27me3	Abcam	Cat# ab6002
Rabbit anti-H3K27ac	Abcam	Cat# ab4729
Rabbit anti-H3K4me1	Cell Signal Technology	Cat# 5326
Rabbit anti-H3K4me3	Abcam	Cat# ab8580
**Oligonucleotides and other sequence-based reagents**		
Olig2-sgRNA	This paper	AGCTGAGCTCCGAGCTACGA
**Chemicals, enzymes and other reagents**		
Retinoic acid (RA)	Sigma-Aldrich	R2625
Smoothened Agonist (SAG)	Selleck Chemicals	S7779
DAPT	Selleck Chemicals	S2215
GSKJ4	Selleck Chemicals	S7070
Hyperactive pG-MNase Cut&Run Assay Kit for Illumina	Vazyme	HD102
Hyperactive Universal Cut&Tag Assay Kit for Illumina Pro	Vazyme	TD904
Nuclear Complex Co-IP Kit	Active motif	#54001
Dual-Glo® Luciferase Assay System	Promega	E2920
**Software**		
Fastp (v0.23.1)	(Chen et al, 2018)	https://github.com/OpenGene/fastp
Bowtie2 (v2.2.9)	(Langmead and Salzberg, 2012)	https://bowtie-bio.sourceforge.net/bowtie2/index.shtml
Picard(v2.11.0)	https://broadinstitute.github.io/picard/	https://broadinstitute.github.io/picard/
SAMtools (v.1.9)	(Li et al, 2009)	https://www.htslib.org/doc/1.9/samtools.html
deepTools (v2.25.0)	(Ramirez et al, 2016)	https://deeptools.readthedocs.io/en/latest/source/deeptools.html
Bedtools (v2.25.0)	(Quinlan, 2014)	https://bedtools.readthedocs.io/en/latest/
MACS2 (v2.2.9.1)	(Zhang et al, 2008)	https://github.com/macs3-project/MACS/releases/tag/v2.2.9.1
HOMER (v4.11.1)	(Heinz et al, 2010)	http://homer.ucsd.edu/homer/
ClusterProfiler (v4.7.1.3)	(Wu et al, 2021)	https://www.bioconductor.org/packages//2.13/bioc/html/clusterProfiler.html
Hisat2 (v2.1.0)	(Kim et al, 2019)	https://daehwankimlab.github.io/hisat2/
HTSeq-count (v0.11.0)	(Anders et al, 2015)	https://htseq.readthedocs.io/en/release_0.11.1/count.html
DESeq2 (1.42.0)	(Love et al, 2014)	https://bioconductor.org/packages/devel/bioc/html/DESeq2.html
ggplot2 (v3.4.4)	(Wickham, 2016)	https://cran.r-project.org/web/packages/ggplot2/index.html
pheatmap (1.0.12)	https://cran.r-project.org/web/packages/pheatmap/index.html	https://cran.r-project.org/web/packages/pheatmap/index.html
R (v4.1.0)	https://www.R-project.org	https://www.R-project.org

### mESC culture and motor neuron differentiation

C57BL/6 mouse embryonic stem cells (mESCs, Oricell) were cultured on a monolayer of Mitomycin C-treated mouse embryonic fibroblasts (MingCeler) in Knockout DMEM medium (Gibco) supplemented with 10% fetal bovine serum (Excell), 5% Knockout serum replacement (Gibco), 100 U/ml penicillin-streptomycin solution (Gibco), 2 mM L-glutamax (Gibco), 1X MEM non-essential amino acid solution (Gibco), 0.1 mM β-mercaptoethanol (Sigma), and 1000 U/ml leukemia inhibitory factor (Sigma). Motor neuron (MN) differentiation was performed as previously described with minor modifications (Mazzoni et al, 2013; Wichterle et al, 2002). Briefly, mESCs were dissociated with 0.25% trypsin (Gibco) into a single cell suspension at 2.5 × 10^4^ cells/ml in ADFNK medium (Advanced DMEM/F12:Neurobasal (1:1) medium (Gibco), 10% knockout serum replacement (vol/vol), 100 U/ml penicillin-streptomycin solution, 2 mM L-glutamax, 1X MEM non-essential amino acid solution and 0.1 mM β-mercaptoethanol). Then 20 μl of cell mixture (500 cells) was seeded onto the cover of a petri dish to initiate formation of embryoid bodies (EBs) (D0: day 0). Two days later (D2: day 2), EBs were harvested and plated into differentiation medium (ADFNK medium supplemented with 1 µM all-trans retinoic acid (RA, Sigma) and 0.5 µM Sonic Hedgehog Agonist (SAG, Selleck) for caudalization and ventralization. Culture Medium was then changed at D3 (day 3). For directed medial motor column (MMC) differentiation, the differentiation medium was maintained for two more days and then changed at D5 (day 5) with ADFNK medium without RA and SAG for later differentiation. For directed hypaxial motor column (HMC) differentiation, 5 µM DAPT (Selleck) was added to the differentiation medium at D4 (day 4) and ADFNK medium at D5 (day 5).

### Immunohistochemistry and imaging

Immunohistochemistry was performed as described before (Wang et al, 2022). Briefly, differentiating neurospheres or chicken spinal cords were collected at indicated stages, fixed for 1.5 h at 4 °C in 4% paraformaldehyde in phosphate-buffered saline (PBS), then immersed in 30% sucrose for 2 h, and then embedded in OCT (Tissue-Tek) and sectioned for immunostaining. 12 µm cryosectioned slices were incubated using the primary antibodies overnight at 4 °C within blocking buffer (2% BSA, 0.15% Triton X-100 in PBS buffer). Antibodies used for immunostaining in this study are as follows: rabbit anti-Olig2 (proteintech 13999-1-Ap, 1:600), mouse anti-Ngn2 (R&D MAB3314, 1:450), rabbit anti-Neurod1 (Cell signaling technology #62953, 1:500), mouse anti-Mnx1 (DHSB 81.5C10, 1:100), rabbit anti-Isl1 (Abcam ab20670, 1:300), mouse anti-Lhx3 (DHSB 67.4E12, 1:100). Then fluorophore-conjugated species-specific secondary antibodies were used as recommended (Proteintech or Jackson ImmunoResearch). Images were acquired using a confocal laser scanning microscope (Zeiss LSM 800) with a 10X objective.

### Cut&Run, Cut&Tag and sequencing (Cut&Run-seq and Cut&Tag-seq)

Cut&Run (cleavage under targets and release using nuclease) assay and library construction for sequencing were performed using Hyperactive pG-MNase Cut&Run Assay Kit for Illumina (Vazyme HD102). Specifically, differentiating MN neurospheres at indicated stages (D4, D4H8, D5, and D6) were harvested, homogenized and nuclei were extracted within NE buffer. Nuclei were lightly crosslinked with 0.1% formaldehyde for 2 min at room temperature and quenched by 125 mM glycine for 5 min. Nuclei were washed with Washing Buffer and mixed with ConA Beads Pro for 10 min. The nuclei-ConA bead complexes were incubated with 3 µg of antibodies overnight at 4 °C. Then, pG-Mnase enzyme incubation, DNA fragmentation, release and extraction were performed according to the manufacturer's manual. DNA fragments were then subjected to library construction with DNA damage repair and end preparation, adapter ligation and library amplification. VAHTS multiplex Oligos Set 5 (Vazyme N322) was used for adapter ligation. Purified DNA was sequenced with PE150 pair ends using the Illumina Novaseq X Plus platform. Kdm6b antibody for Cut&Run was made with three antigens corresponding to amino acids 1-210, 488-715, and 800-1096 of mouse Kdm6b and affinity-purified. And other antibodies for Cut&Run were as follows: anti-Neurod1 (Proteintech 12081-1-AP) and anti-Cux1/2 (Abcam Ab309139).

Cut&Tag (cleavage under target & tagmentation) assay and library construction for sequencing were performed using Hyperactive Universal Cut&Tag Assay Kit for Illumina Pro (Vazyme TD904). Nuclei preparation was identical to the Cut&Run procedure. Histone modifier antibodies were used for primary incubation, followed by secondary antibody incubation. The pA/G-Transposon Pro incubation, DNA tagmentation and extraction were performed according to the Cut&Tag manual. The library construction was performed using Trueprep Index Kit V2 for Illumina (Vazyme TD202). Purified DNA products from PCR amplification were sequenced with PE150 pair ends using the Illumina Novaseq X Plus platform. Antibodies for Cut&Tag were as follows: anti-H3K27me3 (Abcam Ab6002), anti-H3K27ac (Abcam Ab4729), anti-H3K4me1 (Cell Signaling Technology #5326) and anti-H3K4me3 (Abcam Ab8580).

### Bioinformatic analyses of Cut&Run-seq and Cut&Tag-seq

Paired-end reads (150 bp) for Cut&Run-seq and Cut&Tag-seq datasets with a single biological replicate were collected. Fastp (v0.23.1) (Chen et al, 2018) was conducted for trimming raw fastq files to remove adapters and low-quality bases with the following parameter settings: -q 20 --length_required 18 --n_base_limit 5. The trimmed fastq files were then mapped to the mm10 reference genome (Ensembl release 102) utilizing Bowtie2 (v2.2.9) (Langmead and Salzberg, 2012). Picard was used to remove duplicates, and then uniquely mapped reads were obtained using SAMtools (v.1.9) (Li et al, 2009), with the parameter “-q 10 -F1024”. For visualization, deepTools (v2.25.0) (Ramirez et al, 2016) was utilized with the “bamCoverage” function to generate normalized RPM bigWig files. In parallel, RPM (Reads Per Million mapped reads) normalization was performed using bedtools (v2.25.0) (Quinlan, 2014) “genomecov” with the -scale parameter, where the scale factor was calculated as 1,000,000 divided by the total number of uniquely mapped reads in each sample. This normalization minimizes batch effects and allows relative binding intensity to be compared across samples. Binding enrichment was subsequently inspected using the IGV browser. For peak calling, MACS2 (v2.2.9.1) (Zhang et al, 2008) was utilized with “-q 0.05 -f BAMPE” parameter setting. For histone modifications, the --broad option was additionally applied. deepTools was further applied for heatmap or line plot profile visualization with the functions “computeMatrix” and “plotHeatmap” or “plotProfile”. To characterize overlapping peaks, the “mergePeaks” function from HOMER (v4.11.1) (Heinz et al, 2010) was further applied with the parameter -d given specified (≥1 bp overlap).

To identify stage-specific peaks of Kdm6b, the subtract function from deepTools was utilized with the following command: bedtools subtract -A -a <fileA>-b <fileB>. Specifically, common peaks (Com) of Kdm6b were designated as the binding sites shared across stages D4H8, D5 and D6. Stage-specific peaks were obtained by removing common peaks from total peaks per stage, designated as D4-Com, D4H8-Com, D5-Com, and D6-Com, respectively. The common and stage-specific peaks were then annotated to common and stage-specific target genes of Kdm6b respectively according to the nearest promoter principle using the “annotatePeaks.pl” function in HOMER. Motif enrichment analysis was performed with the “findMotifsGenome.pl” function in HOMER, resulting in both known and de novo motif enrichment outputs, of which the known motifs were selected for this study. Gene ontology (GO) pathway enrichment analysis and visualization were conducted using the enrichGO and dotplot functions from the clusterProfiler (v4.7.1.3) (Wu et al, 2021) in the R package (v4.1.0) and data were presented with Biological Process (BP) terms.

### Immunoprecipitation-mass spectrometry (IP-MS) and co-immunoprecipitation (co-IP)

Nuclear Complex Co-IP Kit (Active motif #54001) was employed to immune-precipitate endogenous Kdm6b and its binding proteins in differentiating MNs at indicated stages (D4H8, D5, and D6). Specifically, MN neurospheres were homogenized in a hypotonic buffer and subjected to nucleus isolation and digestion according to the manufacturer's manual. IP was conducted with 3 µg Kdm6b antibody and ProteinA/G agarose beads in 1X low IP buffer with 1 mM dithiothreitol (DTT) and proteinase inhibitor cocktail. Then beads were washed twice, 5 min for each, with wash buffer including 1X low IP buffer with 1 mM DTT, proteinase inhibitor cocktail, 100 mM NaCl and 0.2% detergent. Finally, beads were washed with PBS three times and protein was extracted with triethylammonium bicarbonate (TEAB) buffer. For digestion, the protein solution was reduced with 5 mM DTT for 30 min at 56 °C and alkylated with 11 mM iodoacetamide for 15 min at room temperature in darkness. The protein sample was then diluted by adding 200 mM TEAB to urea concentration less than 2 M. Finally, trypsin was added at 1:50 trypsin-to-protein mass ratio for the first digestion overnight and 1:100 trypsin-to-protein mass ratio for a second 4-h digestion. Finally, the peptides were desalted by a Strata X SPE column. The tryptic peptides were separated on a NanoElute UHPLC system (Bruker Daltonics) and were subjected to a capillary source followed by the timsTOF Pro mass spectrometry (Bruker Daltonics). The DIA data were processed using the DIA-NN search engine (v.1.8). Tandem mass spectra were searched against the Mus_musculus_10090_SP_20231220.fasta (17191 entries) concatenated with a reverse decoy database. FDR was adjusted to <1%.

For transcription factor (TF) overexpression and co-IP assay, mouse Sox2, Olig2, Ngn2, Neurod1, Onecut1/2, Cux1/2, Sp1/3, Klf6, Nkx6-1, Tcf4, Nfya, and human YY1 and ETV6 was cloned into pcDNA3-flag, while mouse Kdm6b-N terminus (1-829AA) and C terminus (825-1641AA) were cloned into pcDNA3-HA vector. HEK293 cells were transfected with Flag-TFs and HA-Kdm6b-N or C and then harvested after 48 h. IP was performed with 2 µg HA, Flag, or IgG control antibodies and ProteinA/G agarose beads within 25 mM Tris-HCl, pH 7.5, 150 mM NaCl, 1 mM EDTA, 1% NP-40, 5% glycerol, and proteinase inhibitor cocktail. Beads were then washed and the identity of immunoprecipitated proteins were evaluated by western blotting.

### RNA sequencing (RNA-seq) analysis

The differentiating MNs at indicated stages (D4, D4H8, D5 and D6) were harvested in TRIzol reagent (Thermo Fisher), and total RNA was extracted based on the instructions from the manual. GSKJ4 (Selleck) was treated one day earlier to inhibit endogenous Kdm6b enzyme activity (i.e., D3H8→D4H8, D4→D5, and D5→D6). mRNA was purified for library construction using poly-T oligo-attached magnetic beads. RNA-Seq data were trimmed by Fastp (v0.23.1) to remove adapters, low-quality bases (Q < 20), and reads shorter than 75 bp. Trimmed data were aligned to the mm10 reference genome (Ensembl release 102) using Hisat2 (v2.1.0) (Kim et al, 2019). The aligned reads were sorted and indexed using SAMtools (v.1.9). HTSeq-count (v0.11.0) (Anders et al, 2015) was used to generate count matrices for each sample. FPKM (fragments per kilobase of transcript per million mapped fragments, FPKM = [Gene fragment count/(Gene length × total mapped fragments)] × 10^9^) values were calculated from raw counts and annotated gene lengths for visualization and descriptive purposes, indicating relative gene expression within and between samples at different differentiation timepoints. We used the DESeq2 (1.42.0) (Love et al, 2014) in the R package to analyze gene expression with count matrices as input. Kdm6b target genes were categorized into three subsets based on their expression dynamics. Kdm6b target genes were categorized into three subsets: upregulated (Up), downregulated (Down), or constant expression (Constant) based on their temporal expression dynamics. Regarding common target genes, we compared gene expression at D6 versus D4 and assigned fold change (FC) ≥1.1 as upregulated (Up), FC ≤0.9 as downregulated (Down), and FC >0.9 but <1.1 as constant expression (Constant). As for D4-Com, D4H8-Com, D5-Com, and D6-Com target genes, their expressions were compared with D6 versus D4, D6 versus D4H8, D5 versus D4, and D6 versus D4, respectively and the same fold change principle was employed to categorize three subsets. Differentially expressed genes (DEGs) were identified based on the following criteria: log_2_FC <0 and *p* < 0.05 (Benjamini–Hochberg method) for upregulation; log_2_FC >0 and *p* < 0.05 for downregulation, and others for nonsignificant change. All data visualizations, including heatmaps, box plots, and volcano plots, were generated in R using ggplot2 (v3.4.4) (Wickham, 2016) and pheatmap (1.0.12) packages.

### CRISPR/Cas9-mediated knockdown

The Olig2-sgRNA (AGCTGAGCTCCGAGCTACGA) was cloned into lentiCRISPRv2, and lentiviral particles were produced in HEK293T cells. mESCs were transduced with the lentiviruses and selected with 1 μg/mL puromycin for 5 days. Surviving colonies were differentiated into pMNs and assessed for Olig2 expression. A colony positive for Olig2 knockdown was used for CUT&RUN-seq.

### Luciferase assay

The selected overlapping binding sites of TFs and Kdm6b was cloned into TK-luciferase vector. P19 cells were cultured in P19 complete medium (Procell, MEMα medium supplemented with 7.5% NCS + 2.5% FBS) and seeded in 96-well plates overnight prior to transfection with Lipofectamine 2000 (Invitrogen). TK constructs, TF-overexpressing plasmids (pcDNA3-Flag-TFs) and a pRL-TK plasmid for normalization was co-transfected. After 8 h, 1 μM GSKJ4 or vehicle (DMSO) was added to the media. Cells were lysed 48 h post-transfection, and cell extracts were assayed for luciferase activity with Dual-Glo® Luciferase Assay System (Promega E2920). The firefly luciferase values were normalized with Renilla luminescence activity. Histograms show mean normalized luciferase units, and error bars represent standard deviation from representative experiments. All assays were independently repeated at least three times.

### Chick in ovo electroporation

In ovo electroporation was performed as described (Wang et al, 2020). Briefly, Hy-Line brown fertilized eggs (Henan, China) were incubated in a humidified chamber at 38 °C, and DNA constructs pCS2-HA-Isl1-Lhx3 with or without pG-SUPER-Kdm6b-shRNA (Estaras et al, 2012) were injected into the lumen of chick embryonic spinal cords at HH stage 13. Electroporation was performed using a square wave electroporator (BTX830), and then embryos were harvested at 2 days after electroporation for analysis.

### Statistical analyses

Statistical tests and *p* values were calculated using the Wilcoxson rank-sum test or one-way ANOVA test as indicated. *p* values less than 0.05 were considered statistically significant. Significance levels are indicated as follows: *p* < 0.05 (*), *p* < 0.01 (**), and *p* < 0.001 (***).

## Supplementary information

Appendix

Peer Review File

Dataset EV1

Dataset EV2

Dataset EV3

Dataset EV4

Dataset EV5

Source data Fig. 2

Source data Fig. 3

Source data Fig. 4

Source data Fig. 5

Source data Fig. 6

Expanded View Figures

## Data Availability

All sequencing data generated have been deposited in Gene Expression Omnibus (GEO) database under accession number GSE306766 (https://www.ncbi.nlm.nih.gov/geo/query/acc.cgi?acc=GSE306766). The source data of this paper are collected in the following database record: biostudies:S-SCDT-10_1038-S44319-026-00808-2.
