# Supplementary material for: Kdm6b-mediated epigenetic coordination of temporal precision during motor neuron differentiation
Source: EMBO Rep. 2026 May 26;27(13):3665–88. doi: 10.1038/s44319-026-00808-2 (PMC13354772; doi:10.1038/s44319-026-00808-2)
Supplement: Supplementary file 1 — Appendix [file 44319_2026_808_MOESM1_ESM.pdf]

Ren et al. Appendix Figures

Table of contents Page #

Appendix Figure S1. 2

Appendix Figure S2. 3

Appendix Figure S3. 4

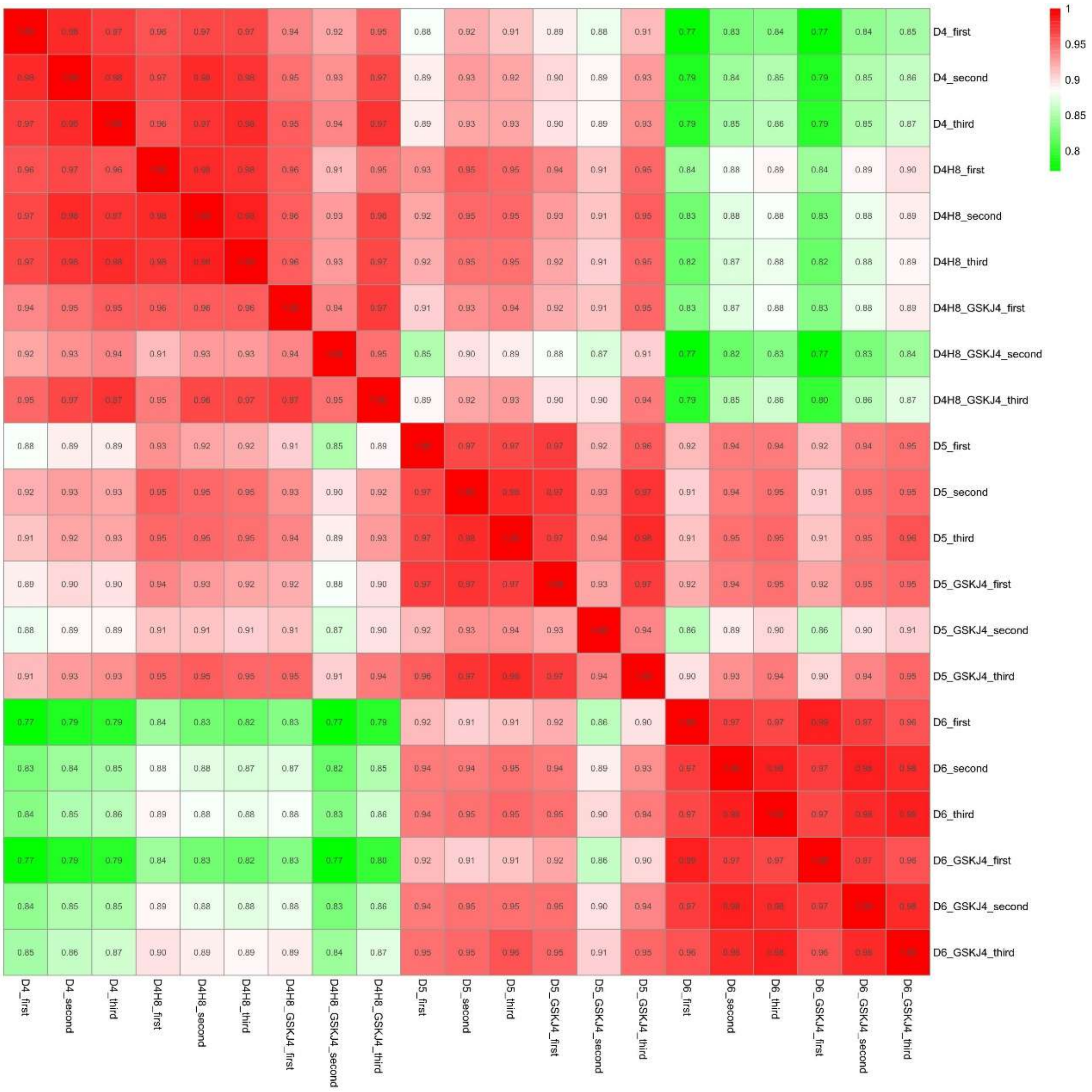

Appendix Figure S1. The pairwise Pearson correlation of RNA-seq datasets. RNA-seq was performed at four differentiation stages (D4, D4H8, D5, and D6) with three biological replicates per each stage (labeled as _first, _second, and _third). For inhibitor treatment, GSK-J4 was added one day prior to collection, also with three replicates. The correlation matrix indicates pairwise Pearson correlation of RNA-seq data between any two samples, with the coefficient indicated as the number.

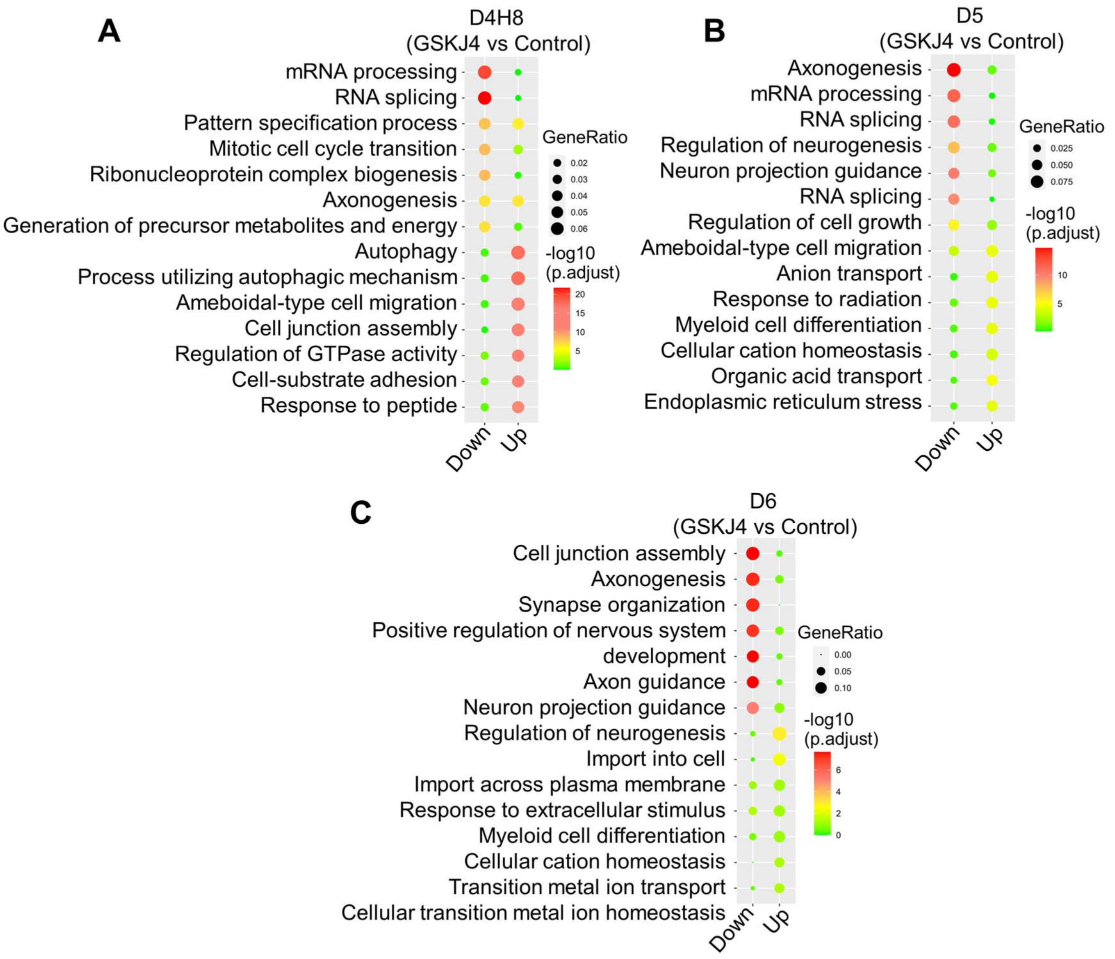

Appendix Figure S2. GO analyses of DEGs of Kdm6b targets with GSKJ4 treatment. (**A**-**C**) Relative to Fig 5B, top 7 GO terms (Biological Process) of downregulated and upregulated genes of Kdm6b targets with GSKJ4 treatment relative to control at the indicated stages D4H8 (A), D5 (B) and D6 (C). Adjusted p value was determined by Wilcox rank-sum test.

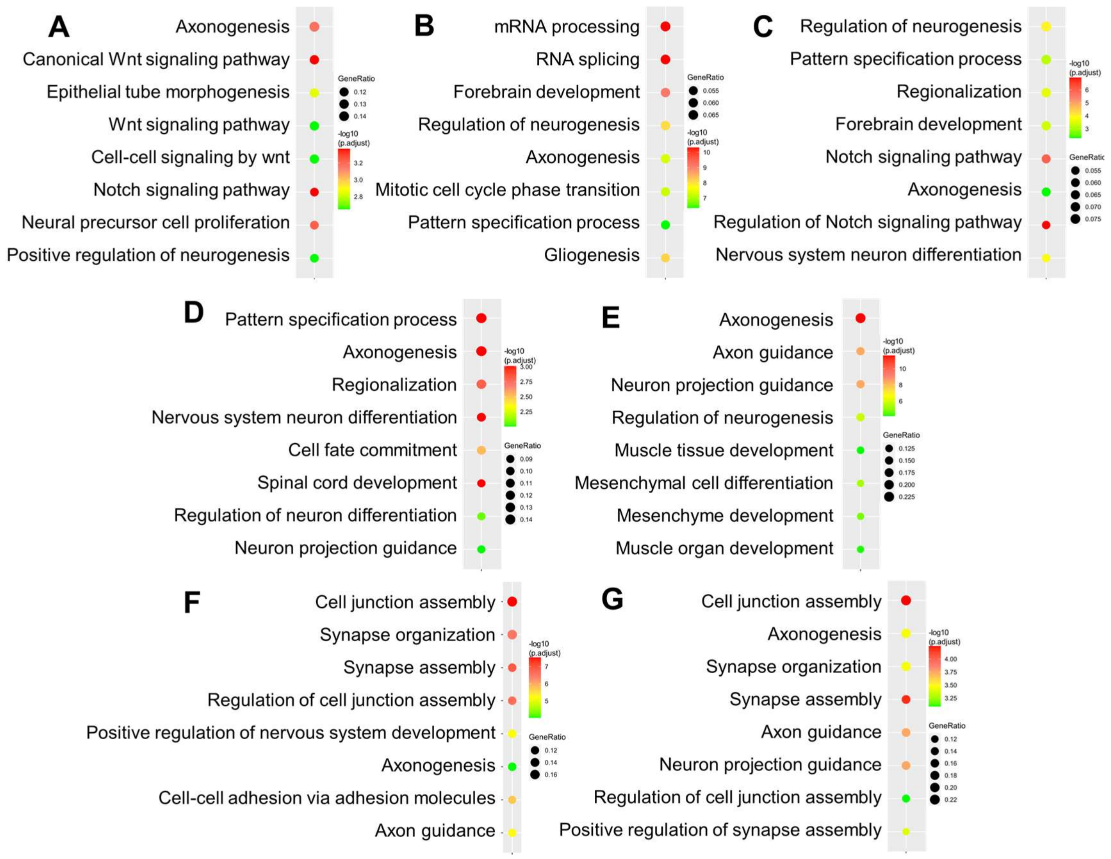

Appendix Figure S3. GO analyses of DEGs of co-targets of Kdm6b and TFs with GSKJ4 treatment. (**A**-**G**) Top 8 GO terms of common targets of Kdm6b and Sox2 at D4H8 (A), Kdm6b and Olig2 at D4H8 (B), Kdm6b and Ngn2 at D4H8 (C), Kdm6b and Neurod1 at D5 (D), Kdm6b and Isl1-Lhx3 at D5 (E), Kdm6b and Onecut1 at D6 (F), Kdm6b and Cux1/2 at D6 (G), among downregulated genes by treatment with GSKJ4 relative to control, as showed in Fig 5B. Adjusted p value was determined by Wilcox rank-sum test.
