# Supplementary material for: Kdm6b-mediated epigenetic coordination of temporal precision during motor neuron differentiation
Source: EMBO Rep. 2026 May 26;27(13):3665–88. doi: 10.1038/s44319-026-00808-2 (PMC13354772; doi:10.1038/s44319-026-00808-2)
Supplement: Supplementary file 13 — Expanded View Figures [file 44319_2026_808_MOESM13_ESM.pdf]

Expanded View Figures

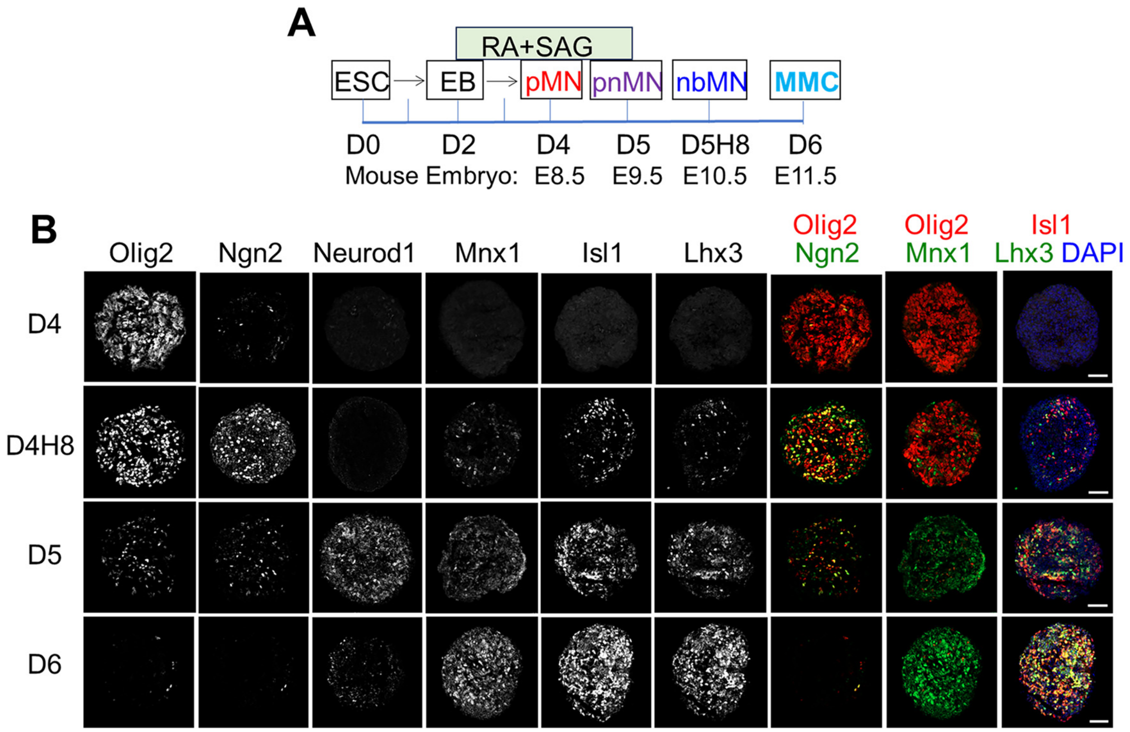

Figure EV1. In vitro directed MN differentiation recapitulates in vivo developmental progression, related to Fig. 1.

(**A**) The diagram represents the in vitro ESC→MN (MMC) differentiation process, with each timepoint approximately corresponding to the mouse embryonic stage. ESC embryonic stem cell, MN motor neuron, EB embryonic body, pMN motor neuron progenitor, pnMN proneural motor neuron, nbMN newborn motor neuron, MMC medial motor column. D day, E embryonic day. (**B**) Immunostaining of Olig2, Ngn2, Neurod1, Mnx1, Isl1, and Lhx3 at MN differentiation stages D4, D5, D5H8, and D6H8. Scale bars: 50 µm.

 © The Author(s)

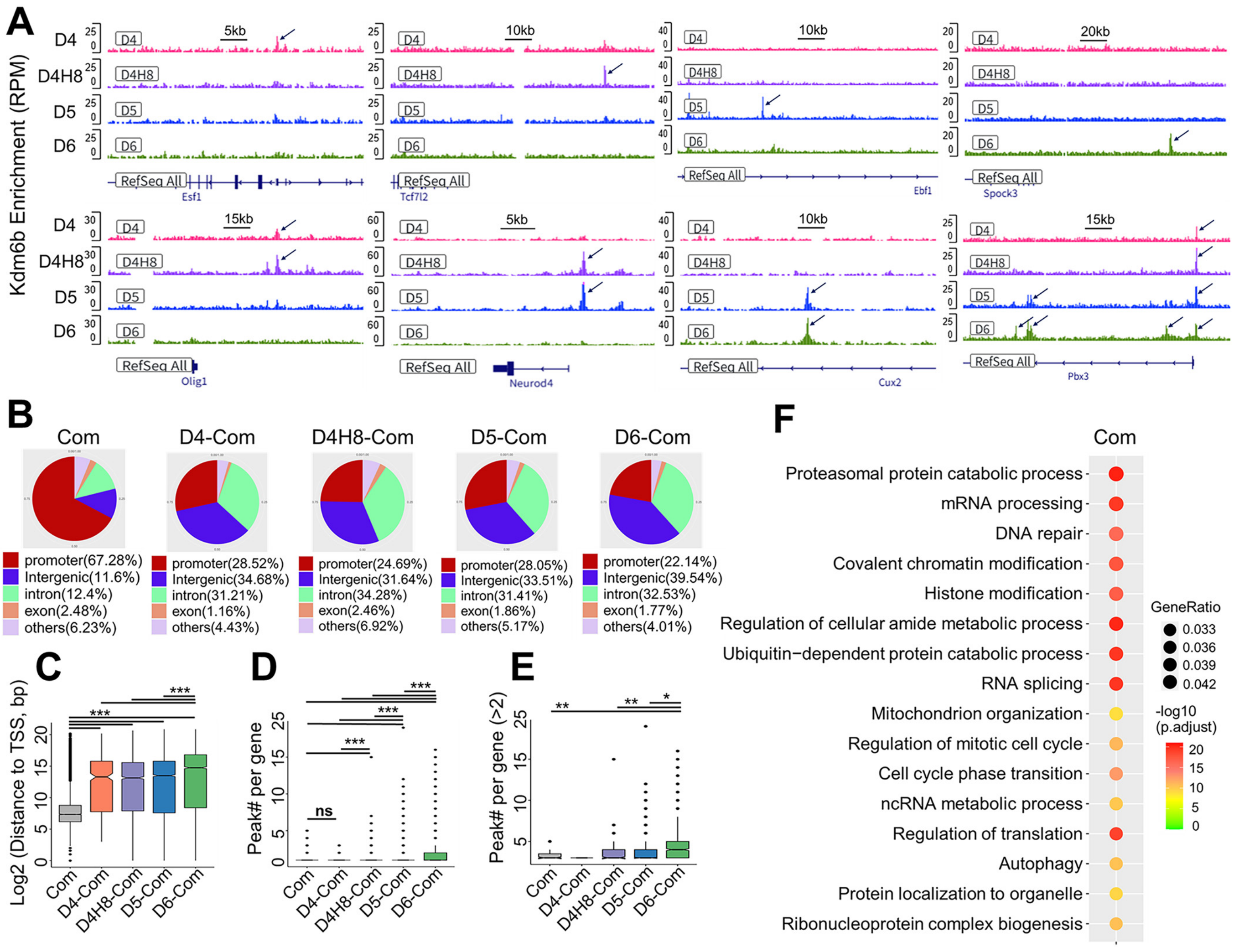

Figure EV2. Dynamic binding patterns of Kdm6b correlate with preferential spatial occupancy and distinct functional significance, related to Fig. 2.

(**A**) Genome browser tracks displaying dynamic Kdm6b enrichment at the selected gene loci across MN developmental stages. RPM indicates enrichment intensity. (**B**) The genomic distribution of Kdm6b common (Com) and stage-specific binding peaks (D4-Com, D4H8-Com, D5-Com and D6-Com) exhibiting spatial preference. (**C**) Box plots showing the distance of Kdm6b common and stage-specific binding sites to the nearest TSSs. *P* value was determined by the Wilcox rank-sum test, and statistical significance was indicated as follows. ***$p < 0.001$ (D6-Com vs Com: 0; D6-Com vs D4-Com: 3.2e-8; D6-Com vs D4H8-Com: 3.9e-54; D6-Com vs D5-Com: 4.3e-73). The specific parameters for each box plot (minimum, 25th percentile, median, 75th percentile, maximum) are: Com (0, 6.2, 7.3, 8.8, 20.2), D4-Com (3, 7.8, 13.3, 15.8, 20.2), D4H8-Com (0, 7.9, 13.1, 15.6, 20.7), D5-Com (0, 7.6, 13.5, 15.8, 20.9), and D6-Com (0, 8.4, 14.8, 16.8, 20.9). The data were from one Cut&Run assay at each stage ($n = 1$). (**D**) Box plots quantifying the number of Kdm6b binding peaks per proximal gene. *P* value was determined by the Wilcox rank-sum test. ns nonsignificant; ***$P < 0.001$ (D4H8-Com vs D4-Com: 1.9e-7; D5-Com vs D4H8-Com: 8.6e-21; D6-Com vs D5-Com: 4.2e-7). The specific parameters for each box plot (minimum, 25th percentile, median, 75th percentile, maximum) are: Com (0, 1, 1, 1, 5), D4-Com (0, 1, 1, 1, 3), D4H8-Com (0, 1, 1, 1, 15), D5-Com (0, 1, 1, 1, 19), and D6-Com (0, 1, 1, 2, 16). The data were from one Cut&Run assay at each stage ($n = 1$). (**E**) Box plots quantifying the number of >2 Kdm6b binding peaks per proximal gene. *P* value was determined by the Wilcox rank-sum test. *$P < 0.05$; **$P < 0.01$ (D6-Com vs Com: 0.002; D6-Com vs D4H8-Com: 0.003; D6-Com vs D5-Com: 0.04). The specific parameters for each box plot (minimum, 25th percentile, median, 75th percentile, maximum) are: Com (3, 3, 3, 3, 5), D4-Com (3, 3, 3, 3, 3), D4H8-Com (3, 3, 3, 4, 15), D5-Com (3, 3, 3, 4, 19), and D6-Com (3, 3, 4, 5, 16). The data were from one Cut&Run assay at each stage ($n = 1$). (**F**) GO analyses of common target genes showing top-ranked enrichment pathways with bubble plot. Biological Process terms are shown.

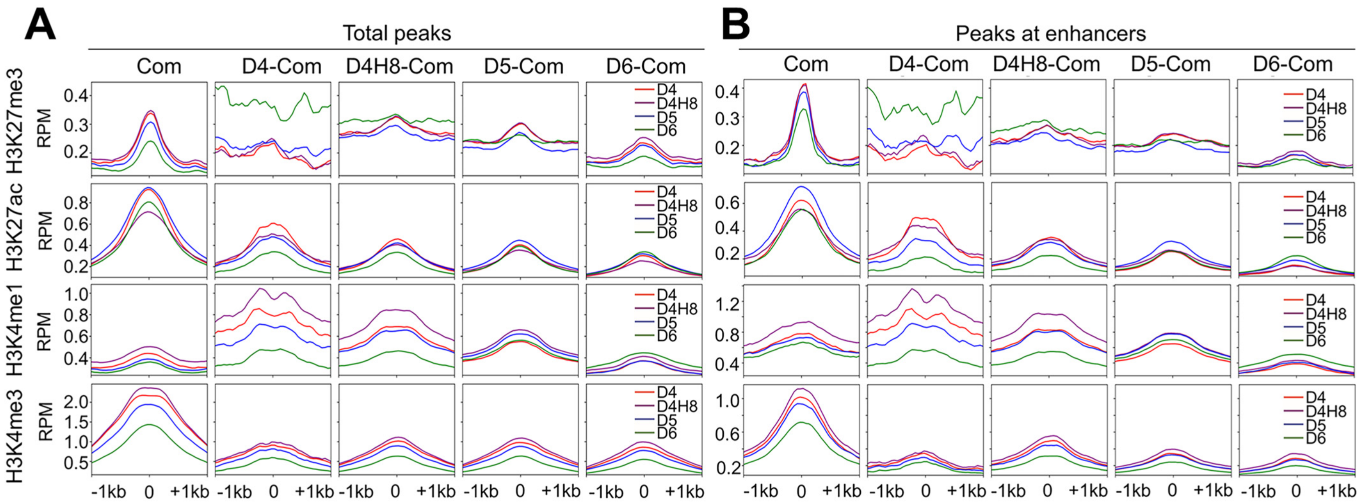

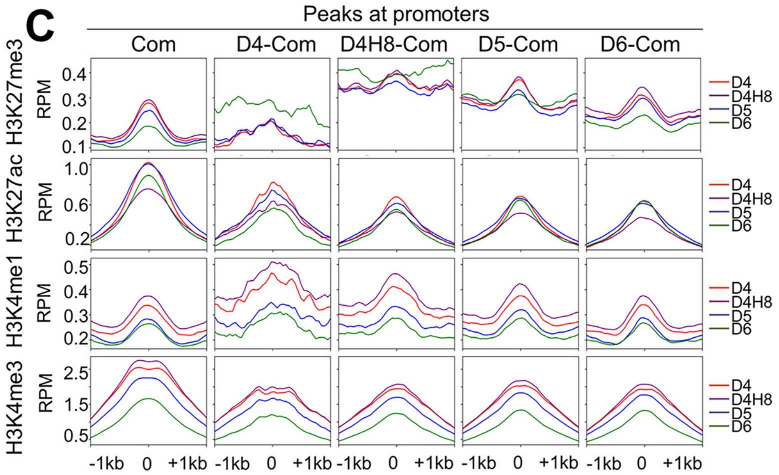

D

Histon modifier	D4H8 peptides	D5 peptides	D6 peptides
EP300	4.5	6.5	2
Setd1a	3.5	4.5	3.5
Setd1b	1.5	2.5	6
Kmt2c	6.5	17.5	14
Kmt2d	34.5	32.5	38.5

Figure EV3. Kdm6b enrichment influences local chromatin modifications, related to Fig. 3.

(**A**) Line plot profiles showing histone modification dynamics within Kdm6b common (Com) and stage-specific binding peaks (D4-Com, D4H8-Com, D5-Com, and D6-Com) across differentiation stages (D4, D4H8, D5, and D6). At stage-specific binding peaks, Kdm6b recruiting was closely associated with low H3K27me3 but high H3K27ac and H3K4me1 levels, while dismantling was largely related to high H3K27me3 but low H3K27ac and H3K4me1 levels. RPM indicates enrichment intensity. (**B, C**) Histon modification dynamics differ between the enhancer areas (**B**) and promoter regions (**C**) of Kdm6b stage-specific binding peaks. H3K4me1 dynamics was restricted to enhancers, while H3K27ac dynamics present at both enhancers and promoters. RPM indicates enrichment intensity. (**D**) Average peptide numbers identified for histone modifiers from Kdm6b IP-MS at the indicated stages from two biologically independent experiments.

 © The Author(s)

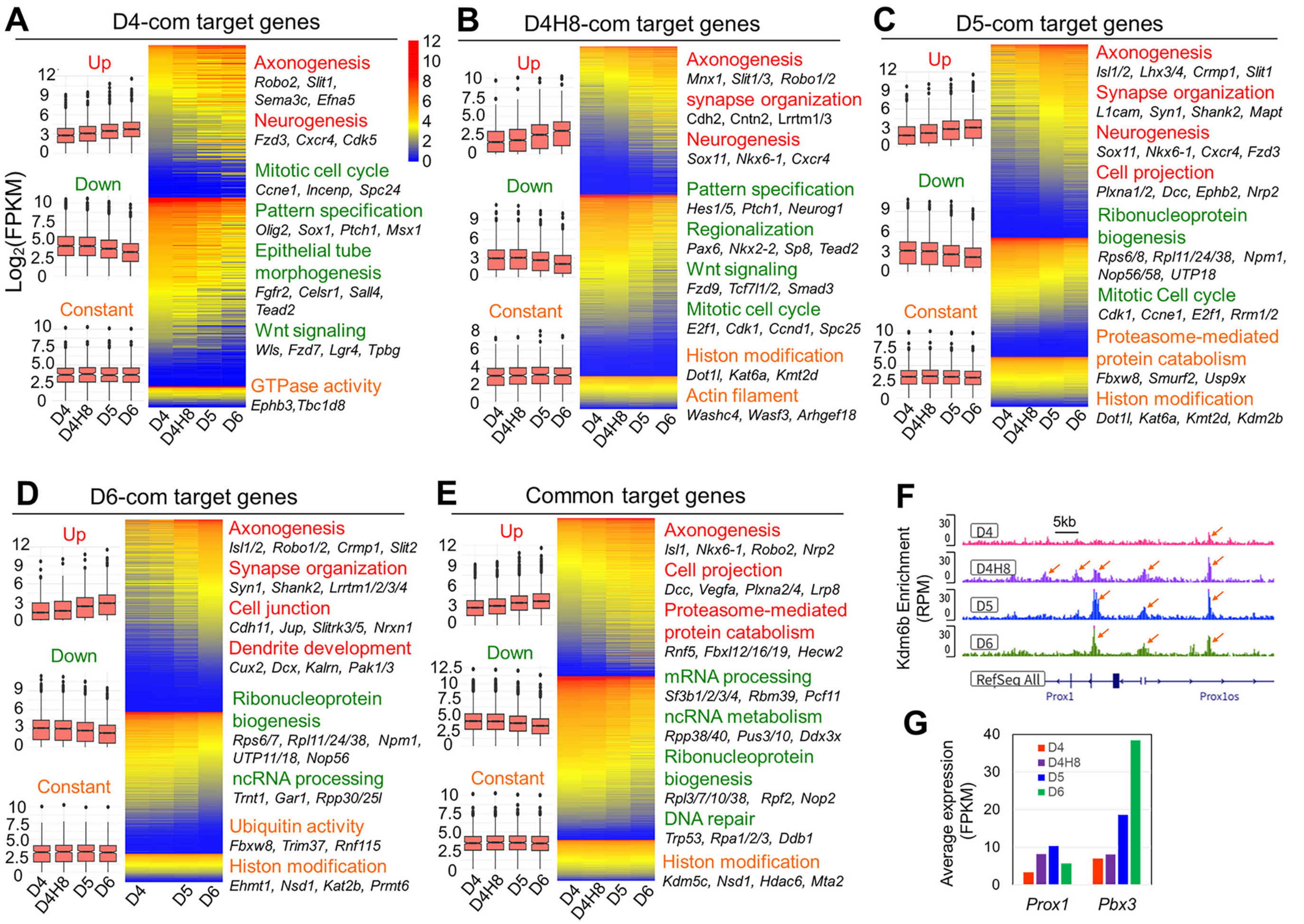

Figure EV4. Kdm6b regulates stage-specific subsets of MN developmental genes, related to Fig. 4.

(**A–E**) Temporal transcription profiles of Kdm6b stage-specific target genes, including D4-Com targets (**A**), D4H8-Com targets (**B**), D5-Com targets (**C**), D6-Com targets (**D**), and common target genes (**E**) during MN differentiation. Box plots and heatmaps depict gene expression profiles categorized as upregulated (Up, fold change ≥1.1), downregulated (Down, ≤0.9) and constant expression (Constant, 0.9 <fold change <1.1), as determined by pairwise comparison in the method section. Each subset underwent GO enrichment analysis, and top nonredundant terms and representative contributing genes are shown. FPKM (fragments per kilobase of transcript per million mapped fragments) was employed to indicate relative gene expression from different samples. (**F**) Genome browser tracks showing Kdm6b utilized both common and stage-specific binding modalities at the Prox1 locus. (**G**) Average mRNA levels of *Prox1* and *Pbx3* from RNA-seq datasets with three biologically independent experiments at each differentiation stage. FPKM was used to indicate relative gene expression from different samples.

© The Author(s)

A

TF	D4H8 peptides	D5 peptides	D6 peptides
ETV6	4	5	1
YY1	10	10	12
Sox2	1	0	0
Olig2	5	3	0
Isl1	3	5	6
Lhx3	2	5	3
Onecut1	4	4	7
Onecut2	0	4	7
Cux1	1	12	18
Cux2	0	0	9

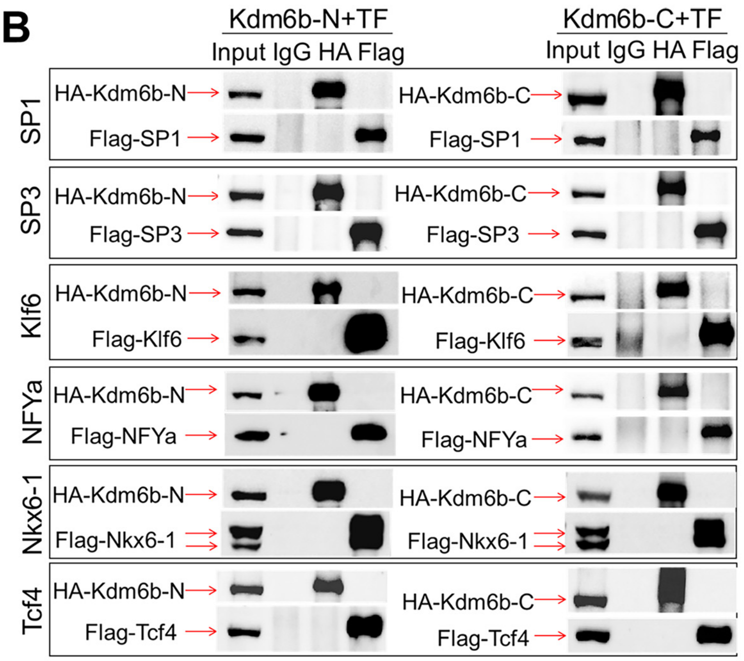

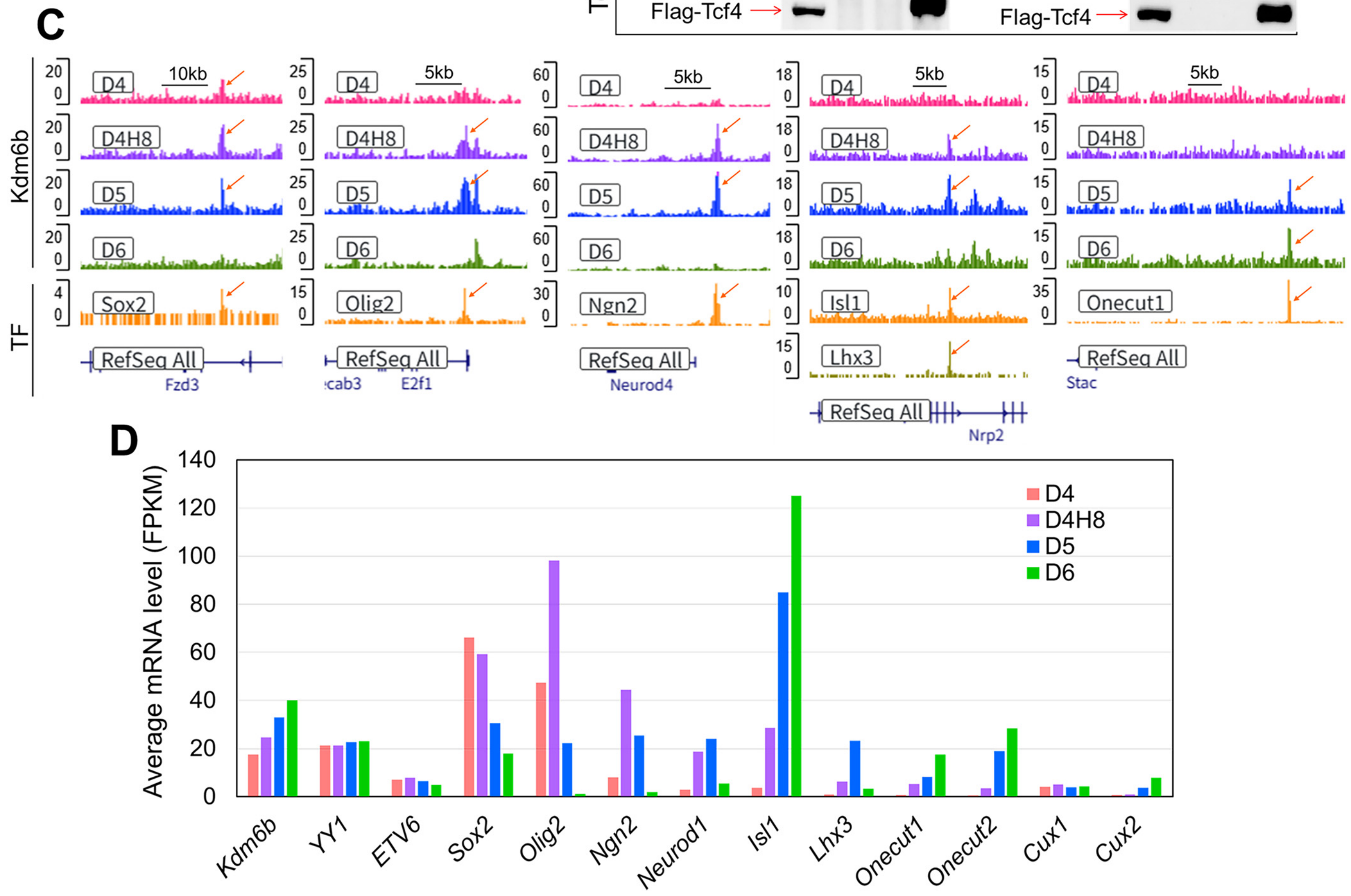

Figure EV5. Kdm6b associates with temporal TFs throughout MN development, related to Fig. 5.

(**A**) Kdm6b-interacting transcription factors identified by IP-MS at the indicated stages. The peptide number, corresponding to each TF, was identified from the second experiment ($n = 1$). (**B**) Co-IP assays with IgG, HA and Flag antibodies within HEK293 cells transfected with HA-Kdm6b-N or C terminus and Flag-TFs, followed by western blotting. The results indicated no interaction between HA-Kdm6b-N or C terminus and tested Flag-TFs. (**C**) Genome browser tracks exhibiting co-enrichment of Kdm6b and developmental TFs at representative genomic loci. (**D**) Average mRNA levels of *Kdm6b* and developmental TFs from RNA-seq datasets at each differentiation stage ($n = 3$ biological replicates). FPKM is used to indicate relative gene expression from different samples.

 © The Author(s)
