# Supplementary material for: Kdm6b-mediated epigenetic coordination of temporal precision during motor neuron differentiation
Source: EMBO Rep. 2026 May 26;27(13):3665–88. doi: 10.1038/s44319-026-00808-2 (PMC13354772; doi:10.1038/s44319-026-00808-2)
Supplement: Supplementary file 2 — Peer Review File [file 44319_2026_808_MOESM2_ESM.pdf]

EMBO reports

Kdm6b-mediated epigenetic coordination of temporal precision during motor neuron differentiation

Haihan Ren, Qicheng Liu, Bingqian Li, Xuetong Yu, Dan Liang, Shuangling Li, Bing Pan, Qian Gao, Wenhong Deng, Jun-Ma Yu, and Wenxian Wang

Corresponding author(s): Wenxian Wang (wxwang@ahmu.edu.cn) , Wenhong Deng (efy101107@fy.ahmu.edu.cn), Jun-Ma Yu (yujunma@ahmu.edu.cn)

Review Timeline:

Submission Date:	23rd Sep 25
Editorial Decision:	18th Nov 25
Revision Received:	30th Mar 26
Editorial Decision:	30th Apr 26
Revision Received:	5th May 26
Accepted:	15th May 26

Editor: Esther Schnapp

Transaction Report:

(Note: With the exception of the correction of typographical or spelling errors that could be a source of ambiguity, letters and reports are not edited. Depending on transfer agreements, referee reports obtained elsewhere may or may not be included in this compilation. Referee reports are anonymous unless the Referee chooses to sign their reports.)

1st Editorial Decision 18th Nov 2025

Dear Prof. Wang,

Thank you for the submission of your manuscript to EMBO reports. We have now received the full set of referee reports that is pasted below.

As you will see, the referees acknowledge that the findings are interesting. However, they also have a number of suggestions for how the study could be improved and strengthened. I think all suggestions are good and should be addressed, but please let me know in case you disagree and we can discuss the exact revision requirements further, also in a video chat, if you like.

I would thus like to invite you to revise your manuscript with the understanding that the referee concerns must be fully addressed and their suggestions taken on board. Please address all referee concerns in a complete point-by-point response. Acceptance of the manuscript will depend on a positive outcome of a second round of review. It is EMBO reports policy to allow a single round of major revision only and acceptance or rejection of the manuscript will therefore depend on the completeness of your responses included in the next, final version of the manuscript.

We realize that it is difficult to revise to a specific deadline. In the interest of protecting the conceptual advance provided by the work, we recommend a revision within 3 months (18th Feb 2026). Please discuss the revision progress ahead of this time with the editor if you require more time to complete the revisions.

IMPORTANT NOTE: we perform an initial quality control of all revised manuscripts before re-review. Your manuscript will FAIL this control and the handling will be DELAYED if the following APPLIES:
1) A data availability section providing access to data deposited in public databases is missing. If you have not deposited any data, please add a sentence to the data availability section that explains that.
2) Your manuscript contains statistics and error bars based on n=2. Please use scatter blots in these cases. No statistics should be calculated if n=2.

When submitting your revised manuscript, please carefully review the instructions that follow below. Failure to include requested items will delay the evaluation of your revision.

1) a .docx formatted version of the manuscript text (including legends for main figures, EV figures and tables). Please make sure that the changes are highlighted to be clearly visible.

2) individual production quality figure files as .eps, .tif, .jpg (one file per figure). See https://wol-prod-cdn.literatumonline.com/pb-assets/embo-site/EMBOPress_Figure_Guidelines_061115-1561436025777.pdf for more info on how to prepare your figures.

3) We replaced Supplementary Information with Expanded View (EV) Figures and Tables that are collapsible/expandable online. A maximum of 5 EV Figures can be typeset. EV Figures should be cited as 'Figure EV1, Figure EV2" etc... in the text and their respective legends should be included in the main text after the legends of regular figures.

- For the figures that you do NOT wish to display as Expanded View figures, they should be bundled together with their legends in a single PDF file called *Appendix*, which should start with a short Table of Content. Appendix figures should be referred to in the main text as: "Appendix Figure S1, Appendix Figure S2" etc. See detailed instructions regarding expanded view here: <https://www.embopress.org/page/journal/14693178/authorguide#expandedview>

- Additional Tables/Datasets should be labeled and referred to as Table EV1, Dataset EV1, etc. Legends have to be provided in a separate tab in case of .xls files. Alternatively, the legend can be supplied as a separate text file (README) and zipped together with the Table/Dataset file.

4) a .docx formatted letter INCLUDING the reviewers' reports and your detailed point-by-point responses to their comments. As part of the EMBO Press transparent editorial process, the point-by-point response is part of the Review Process File (RPF), which will be published alongside your paper.

5) a complete author checklist, which you can download from our author guidelines <https://www.embopress.org/page/journal/14693178/authorguide>. Please insert information in the checklist that is also reflected in the manuscript. The completed author checklist will also be part of the RPF.

6) Please note that all corresponding authors are required to supply an ORCID ID for their name upon submission of a revised manuscript (<https://orcid.org/>). Please find instructions on how to link your ORCID ID to your account in our manuscript tracking system in our Author guidelines <https://www.embopress.org/page/journal/14693178/authorguide#authorshipguidelines>

7) Before submitting your revision, primary datasets produced in this study need to be deposited in an appropriate public

database (see https://www.embopress.org/page/journal/14693178/authorguide#datadeposition). Please remember to provide a reviewer password if the datasets are not yet public. The accession numbers and database should be listed in a formal "Data Availability" section placed after Materials & Method (see also https://www.embopress.org/page/journal/14693178/authorguide#datadeposition). Please note that the Data Availability Section is restricted to new primary data that are part of this study. * Note - All links should resolve to a page where the data can be accessed. *
If your study has not produced novel datasets, please mention this fact in the Data Availability Section.

8) At EMBO Press we ask authors to provide source data for the main manuscript figures. You will receive a separate email with instructions for providing source data with your revised manuscript, including how to upload and organize the files.

9) Our journal also encourages inclusion of *data citations in the reference list* to directly cite datasets that were re-used and obtained from public databases. Data citations in the article text are distinct from normal bibliographical citations and should directly link to the database records from which the data can be accessed. In the main text, data citations are formatted as follows: "Data ref: Smith et al, 2001" or "Data ref: NCBI Sequence Read Archive PRJNA342805, 2017". In the Reference list, data citations must be labeled with "[DATASET]". A data reference must provide the database name, accession number/identifiers and a resolvable link to the landing page from which the data can be accessed at the end of the reference. Further instructions are available at https://www.embopress.org/page/journal/14693178/authorguide#referencesformat

10) Regarding data quantification (see Figure Legends: https://www.embopress.org/page/journal/14693178/authorguide#figureformat)

The following points must be specified in each figure legend:

- the name of the statistical test used to generate error bars and P values,

- the number (n) of independent experiments (please specify technical or biological replicates) underlying each data point,

- the nature of the bars and error bars (s.d., s.e.m.),

- If the data are obtained from n Program fragment delivered error ``Can't locate object method "less" via package "than" (perhaps you forgot to load "than"?) at //ejpvfs23/sites23b/embor_www/letters/embor_decision_revise_and_review.txt line 56.' 2, use scatter blots showing the individual data points.

Discussion of statistical methodology can be reported in the materials and methods section, but figure legends should contain a basic description of n, P and the test applied.

- Please also include scale bars in all microscopy images.

11) The journal requires a statement specifying whether or not authors have competing interests (defined as all potential or actual interests that could be perceived to influence the presentation or interpretation of an article). In case of competing interests, this must be specified in your disclosure statement. Further information: https://www.embopress.org/competing-interests

12) All Materials and Methods need to be described in the main text using our 'Structured Methods' format, which is required for all research articles. According to this format, the Methods section includes a Reagents and Tools Table (listing key reagents, experimental models, software and relevant equipment and including their sources and relevant identifiers) followed by a Methods and Protocols section describing the methods using a step-by-step protocol format. The aim is to facilitate adoption of the methodologies across labs. More information on how to adhere to this format as well as a downloadable template (.docx) for the Reagents and Tools Table can be found in our author guidelines: https://www.embopress.org/page/journal/14693178/authorguide#structuredmethods.

An example of a Method paper with Structured Methods can be found here: https://www.embopress.org/doi/full/10.1038/s44320-024-00037-6#sec-4

We would also welcome the submission of cover suggestions, or motifs to be used by our Graphics Illustrator in designing a cover.

As part of the EMBO publication's Transparent Editorial Process, EMBO reports publishes online a Review Process File (RPF) to accompany accepted manuscripts. This File will be published in conjunction with your paper and will include the referee reports, your point-by-point response and all pertinent correspondence relating to the manuscript.

You are able to opt out of this by letting the editorial office know (emboreports@embo.org). If you do opt out, the Review Process File link will point to the following statement: "No Review Process File is available with this article, as the authors have

chosen not to make the review process public in this case."

I look forward to seeing a revised form of your manuscript when it is ready.

Yours sincerely,

Esther Schnapp, PhD
Senior Editor
EMBO reports

Referee #1:

Summary of the Paper:
Histone modifiers, such as Kdm6b, generally lack sequence specificity. They rely on recruitment to specific genomic regions by other factors. This study investigates how Kdm6b achieves the temporal precision required for motor neuron (MN) differentiation during cellular development. Using an in vitro mouse embryonic stem cell (mESC)-to-MN differentiation model, the authors examine Kdm6b's dynamic interactions with stage-specific transcription factors (TFs) to facilitate stepwise MN development.

The results show that Kdm6b drives precise gene activation through dynamic repositioning across the genome-from promoters to distal enhancers-and sequential associations with TFs like Olig2, Ngn2, Isl1, and Cux1. Kdm6b remodels chromatin by removing the H3K27me3 repressive mark and recruiting activation-related histone modifications, such as H3K27ac and H3K4me1. Disruption of Kdm6b activity, either through inhibition or removal, blocks differentiation at specific stages, emphasizing its crucial role in the gene regulatory network underlying MN development.

The study concludes that Kdm6b acts as a key epigenetic regulator, coordinating stepwise MN differentiation in conjunction with stage-specific TFs. The authors refine their previous model by demonstrating how Kdm6b's recruitment by TFs drives its dynamic repositioning and chromatin modification activity. Robust genome-wide data, supported by techniques such as co-immunoprecipitation and IP-MS, validate the proposed model. This work represents significant progress toward understanding Kdm6b's mechanism of action. However, the clarity of data presentation could be improved, and additional experiments might further strengthen the findings. Overall, the study provides a compelling model of Kdm6b's role in MN differentiation and enriches our understanding of epigenetic regulation during development.

Major general comments:
It is my opinion that there are two important experiments to support the.

1) The Kdm6b inhibitor experiment will benefit from some more characterization, at a minimum H3k27me3 Cut&Tag and a description of how it affects MN differentiation. Does it reduce the number of cells transitioning to the next developmental stage? Do the mis-regulated genes maintain H3k27me3? This data will be critical to identify direct GSK-J4 effects vs indirect and build the regulatory model during MN differentiation not with luciferase assays as the authors have all the rest of the data (see below).
2) Ideally, the authors will challenge the model to the core by analyzing Kdm6b binding in a TF mutant. This is, however, a more demanding experiment but the one that will cement their claims.

Specific comments:
Overall there is a bit of overclaim in their description of the results and description. I encourage the authors to state claims close to their data.
For example this sentence is not sufficiently supported by the data: "This study revealed Kdm6b recruitment initiates a coordinated histone modification cascade: localized H3K27me3 reduction is accompanied by rapid H3K27ac deposition and H3K4me1 enrichment (H3K27me3↓ / H3K27ac↑ / H3K4me1↑)." They could be independent processes not initiated by Kdm6b.
Similarly, the title "Kdm6b dynamically choreographs genomic landscapes of histone modifications" is based solely on correlations not causality.

Specific comments related to findings in each figure:
Fig 1: This figure described an already well described method. It is valuable and contextualized the work, but it could be a supplementary figure with EV1 bringing other more novels aspects of this work to the forefront.
Fig EV5A: It was hard for this reviewer to understand the labels in Fig EV5A and extract any other conclusion than the inhibitor does not produce massive transcriptional changes and D5GSK second is an outliner.
Fig 2C: Just a note of caution for the authors. Cut&Tag has a well-recognized bias towards accessible regions, thus proximal regions and TSS, specially in early differentiation states. I am wondering if this is what skewing some of the early proximal vs late distal and it is a gain of Kdm6b binding in regulatory regions as as cells differentiate. An equally or more exciting pattern.
Fig 3B: the number of peptides, biological replicates and statistics needs more clarification. Is 1 peptide considered enrichment?
Fig 3F: are the numbers on then Venn diagram correct? It seems the total number o sites is represented as exclusive.
Fig 4: I am sure I missed it, but the number of biological replicates and how it was quantified was not very obvius to me.
Fig 4: This is a complex figure that to me supports Fig 2 because as far as I can tell prescind of any TF association data.

Fig 4B: Since the authors might want to highlight the dynamics, would a "subway map" or any other graphic from to highlight transition would be a better representation? I tis hard to understand the meaning and extract information in its current form.
Fig 5: This result section presented significant challenges for this reviewer. It was hard to extract what the authors wanted to highlight. What is te meaning/value of GO terms? What is here that is not expected from what we know of gene expression and Kdm6b activity? I left agreeing with this statement that I broad and perhaps unnecessary: "These findings collectively indicate that the transcriptional network governing MN differentiation is highly complicated, crucially but not solely dictated by Kdm6b binding."
Fig 6: the perturbation of the system is welcomed. Have the authors show that the effect of GSK-J4 on chromatin modifications? The results support the know Kdm6b role in promoting differentiation. Perhaps the interesting and novel aspect of this work is correlating the transcriptional changes with Kdm6b binding and TF occupancy which I could not easily extract from any of the figures.
There is an abrupt jump into this conclusion without the proper comparisons: "Thereby, these findings demonstrate that Kdm6b enzyme activity for removing H3K27me3 is required for the timed activation of co-targets of Kdm6b-TF and strongly support a model where Kdm6b collaborated with stage-specific TFs to precisely regulate gene expression programs to drive stepwise differentiation."
I value the revisionist experiments n Fig 6B, but the authors have the data to do it genome wide during MN differentiation to support their point.
Perhaps reorganizing the figures with Fig 3 and Fig 6B as final figure would allow the authors to craft the importance of Kdm6b dynamics, ifs phenotype and end with the mechanisms behind Kdm6b dynamics recruited by TFs.
Fig 6C: It is hard for me to understand the value of these experiments in the way they are presented.
Fig 7: There is a typo in Neurod1

Minor: I noted the punctuation sometimes is on the wrong side of the citations.

Referee #2:

The Study by Ren et al. follows up previous work from Wang et al 2022 describing the role of Kdm6b in driving MN development, which they had previously shown in vivo. To study the specific targets of Kdm6b and how it finds these targets, the authors leverage the use an mESC to MN protocol that has been previously published to perform geneomics experiments and demonstrate sequential co-binding to different TFs during MN development. The authors additionally validate this through a series of elegant luciferase reporter assays. Collectively, the study provides insights into mouse MN development and the role of Kdm6b. The authors collect valuable and unique datasets, however, the presentation of the data could be clarified for better comprehension.

Major commment:
Figures - in general the figure legends need more descriptive to give the reader more direction on how to interpet the data and include the technical or biological n used, the transtical test being used etc. Many of the genomics graphs are missing scales and information about what type of metric is being plotted and how it is normalized. Some specific points and questions are precised below.
Figure 1:
• I was personally missing the expression of Kdm6b at the different stages of the differentation, and potentially also the TFs that will then be important for the dyanmic changes later on.
Figure 2
• 2A - add a scale bar to the CUT&TAG enrichment plot.
Figure 3:
• In panel E, at which time point is the Neurod1 chip performed?
Figure 4:
• I find panel A hard to read and hard to interpret. The final message is that there are many overlapping sites later on? Numbers are hard and small to read.
Figure 5:
• In the RNA-expression data, I am a little bit lost following the analysis. For a gene to be considered up or down regulated, at how many time points does it need to be going up or down?
• How are these graphs scaled? Do the number represent TPMs, RPKM etc? Again, as with all the other figures, more details need to be included in the legend so that they are easy to interpret.
• What is the scale on the box plots?
• I frankly have a difficult time interpreting the main message of this figure.

Figure 6:
• The authors miss the opportunity to compare the overlap between genes whose expression is altered after the GSK treatment and the different Kdm6b targets. This is given as the supplementary data, but could be nicely represented alongside the RNA-seq data, which alone is less interesting in this context.

In general, I think it's a bit difficult to compare the trends as a total number of binding sites, because these change between the different time points. I think it would be more intuitive to express overlaps between Kdm6b and the TFs as a percentage of all possible (i.e. profiled) Kdm6b sites.

Using the mass-spec experiment described, and the previous in vivo data, do the authors observe Kdm6a expression in the MN cultures? This could be worth discussion as it is equally inhibited by GSK-J4.
The authors note that " Notably, a fraction of key MN genes (e.g., Pbx3 in Fig EV2A and Prox1 in Fig EV5D) were subject to dual regulation through both common and stage-specific binding sites, unveiling the utilization of multiple binding modalities for exquisite expression control." Is there a different in the level of expression in these genes based on the presence of additional stage-specific binding sites at certain time points?
Methods/techniques:
1) What kit was used (if any) was used for library consturction. What were the sequencing parameters? What parameters were used in Deseq2 to designate differeentially expressed genes beyond the FC (i.e. p-adj. value, SE etc)? Did you perform additional correction to look at the different time points in your series?
2) To assess the specificity of the antibodies and the library quality, was a IgG only negative control reaction performed for CUT&TAG experiments and Chip-seq experiment?

This is more of a philosophical point, but the title of the paper is Kdm6b-mediated choreography, however, in fact that what authors show is that Kdm6b is a constant act and is being choreographed by the presence of different TFs that appear and disappear over the course of the differentation timeline. Thus, I think it would be more appropriate to turn the title to something like "Temporable Transcription Factor Expression Guides Kdm6b-mediated epigenome remodeling during motor neuron differentiation"

Minor points:
• Names of genes are incorrectly formatted (italics etc), upper case for proteins.

Referee #3:

In the manuscript by Ren et al., the authors investigate the role of the histone lysine demethylase Kdm6b during the directed differentiation of mouse ESC into motor neurons. The authors propose a model whereby the sequential association of Kdm6b with stage-specific TFs instruct the temporal onset of developmental programs and the overall progression towards terminal differentiation and maturation. This work, expand on previous work from the same laboratory (Wang et al., 2022) that identified a role for Kdm6b in regulating the specification of motor neuron subtypes via functional interaction with the motor neurons -specific TFs Isl1-Lhx3.

The dynamic regulation of H3K27 (de)methylation for setting timing during neural differentiation and maturation and the contribution of kdm6b in this process has been already reported in vitro and in vitro and across distinct neuron types. This manuscript introduces a further layer of molecular complexity providing a model on how the regulation of the epigenetic landscape during neural differentiation is achieved with fidelity and cell-type specificity (i.e., association with Kdm6b with TFs). These finding are of potential interest for the field. However, there are a series of concerns that would need to be addressed. I elaborate on the main points below.

- In Fig. 1, the authors provide a characterization of the expression of key markers during mouse ESC motor neuron differentiation. Another example is provided in Fig. EV1. The images convey well the partially overlapping and subsequent expression of key TF. However, a quantitative assessment is missing. Given the importance of the experimental platform for capturing discreet developmental stages based on the timed expression of these TFs, the authors should provide evidence for the robustness and reproducibility of their differentiation method.

- The authors performed Kdm6b CUT&RUN during motor neurons differentiation and conclude that Kdm6b occupancy at specific genomic loci is stage specific. However, the tornado plots in Fig. 2A, show a gradual increase in Kdm6b occupancy during the differentiation, rather than discreet, stage -specific patterns of binding to gene subsets. Similar findings are reported in Fig. 5, where two major trends of gene expression (gradual up and down -regulation) dominate the dataset and subsequent transcriptional programs are not observed. At the light of these observations, the authors findings indicate that Kdm6b may regulated a 2-stage process: proliferation vs. post-mitotic maturation. This is consistent with axonogenesis and synapse organization as the most enriched GO for putative Kdm6b targets at later stages (Fig. 2G). In absence of discreet signatures downstream of stage specific TFs is difficult to assess whether Kdm6b control the temporal progression/directionality of a developmental process in a stepwise manner. The authors could further analyze the RNAseq data (Fig. 5) to test whether such discreet stages are captured in their experimental system and, if present, use the associated transcriptional signatures for the

subsequent analysis.

- The progressive shift in Kdm6b occupancy at intergenic regions versus promoters is clearer. This might suggest that kdm6b at later stages is associated with activity-dependent TFs/programs, which primarily act at enhancer sequences. This interpretation would be consistent with the expected increase in neuronal activity at later stages. I wonder whether the authors could address this and expand the analysis in Fig. 3A by performing and unbiasedly show additional tests of TF motif enrichment.

- In Fig. 3F, it would be informative to assess the nature/known function of the genes co-enriched and non-coenriched between Kdm6b and Sox2, Ngn2, Olig2, Isl1-Lhx3, Onecut1, Cux.

- The line plots in Fig 4B show a largely inverse correlation between H3K27me3 and H3K27ac and H3K4me1, especially in the four columns on the left. This is consistent with the known association of such markers with gene repression versus activation respectively and with the role of Kdm6b as an H3K27 demethylases. However, the conclusion that Kdm6b is the master regulator of those patterns is not directly supported by the data. Most of the plots show very subtle differences which makes their interpretation challenging. Furthermore, functional experiments (e.g., CUT&RUN upon Kdm6b inhibition) are missing.

- The functional experiments shown in Fig. 6 are interesting, as they suggest that TFs guide Kdm6b to specific chromatin loci to remove the H3K27me3 epigenetic repression and thereby achieve full gene activation. Would the forced overexpression of late TFs in Kdm6b knock out mouse ESC (or upon Kdm6b inhibition) prevent the programming of ESC into motor neurons?

- The authors proposed a mechanism by which a promoter-to-enhancer shift in Kdm6b occupancy orchestrates timed gene expression during motor neuron differentiation. Previous reports indicate that the dynamics of resolution of chromatin bivalency at promoters of developmental genes is also a key regulator of the timing of developmental progression. While those two models are rather complementary that in contrast, I feel it would be important to assess chromatin bivalency and its dynamics in the experimental system used in the manuscript, in addition to discuss more broadly the two models in the discussion.

- At the light of the previous points, the model proposed in Fig.7 is not fully supported by the experimental data

- Several evidence from recent articles should be included in the introduction to provide the reader with a more comprehensive view of the literature. These include a series of article on mouse cerebellum development in vivo and on Ezh2/Kdm6b regulation (Matlik et al., PMID: 37491148, Ramesh et al., PMID: 37092728, Tang et al., PMID: 32679064, Wijayatunge et a., PMID: 29254825. Moreover, two recent articles using human stem cell systems have showed that PRC2/H3K27me3 dynamics and other chromatin regulators regulate the timing of neuronal maturation (Ciceri et al., PMID: 38297124) and the temporal control over cell fate specification and neurogenesis across differentiation stages and lineages (Xu et al., PMID: 40897805). These articles should be included in the introduction as well.

Dear reviewers,

We sincerely thank all three reviewers for your appreciation of the significance of our study and for your insightful and constructive comments. In the following pages, we provide point-by-point responses and clarifications, which are shown in blue. Corresponding revisions in the manuscript are also highlighted in blue. In addition, we have reorganized the figures in response to your suggestions. To avoid any confusion regarding the figure numbering, we have included a conversion table for reference.

Old Figure	New Figure	Content	Changes
Fig 1	Fig 1	In vitro directed MN differentiation recapitulates in vivo developmental progression	Unchanged
Fig 2	Fig 2	Genome-wide enrichment of Kdm6b reveals a dynamic binding pattern during MN development	Fig 2A modified
Fig 4	Fig 3	Kdm6b dynamically coordinates histone modification landscapes	Fig 3A and 3C modified
Fig 6	Fig 4	Inhibiting Kdm6b impairs the temporally ordered expression of developmental MN genes	Old Fig 6A kept as Fig 4B; Fig 4A,4C-E added
Fig 3	Fig 5	Kdm6b stage-specifically partners with developmental TFs throughout MN development	Old Fig 3A-D kept as new Fig 5A-D
Fig3, Fig 6	Fig 6	Kdm6b is essential for transcriptionally regulating its co-target genes with temporally associated TFs	Old Fig 3E-F kept as New Fig 6A-B; Old Fig 6B kept as New Fig 6E; Fig 6C-D, F-G added
Fig 7	Fig 7	Schematic representation of Kdm6b as an epigenetic regulator to coordinate step-wise MN development	Modified
Fig EV1	Fig EV1	In vitro directed MN differentiation recapitulates in vivo developmental progression, related to Figure 1	Unchanged
Fig EV2	Fig EV2	Dynamic binding patterns of Kdm6b correlate with preferential spatial occupancy and distinct functional significance, related to Figure 2	Unchanged
Fig EV4	Fig EV3	Kdm6b enrichment influences local chromatin modifications, related to Figure 3	Old Fig EV4A,C kept as New Fig EV3A,D (modified); Fig EV3B-C added

Fig 5, Fig EV5	Fig EV4	Kdm6b regulates stage-specific subsets of MN developmental genes, related to Figure 4	Old Fig 5A-D kept as New Fig EV4A-D; Old Fig EV5C-D kept as New Fig EV4E-F; Fig EV4G added
Fig EV3	Fig EV5	Kdm6b associates with temporal TFs throughout MN development, related to Figure 5	Old Fig EV3A-C kept as New Fig EV5B-D; Fig EV5A added
Fig EV5	Appendix Fig S1	The pairwise Pearson correlation of RNA-seq datasets	Old Fig EV5A kept as New Fig S1
Fig EV6	Appendix Fig S2	GO analyses of DEGs of Kdm6b targets with GSKJ4 treatment	Unchanged
Fig EV7	Appendix Fig S3	GO analyses of DEGs of co-targets of Kdm6b and TFs with GSKJ4 treatment	Unchanged

Referee #1:

Summary of the Paper:

Histone modifiers, such as Kdm6b, generally lack sequence specificity. They rely on recruitment to specific genomic regions by other factors. This study investigates how Kdm6b achieves the temporal precision required for motor neuron (MN) differentiation during cellular development. Using an in vitro mouse embryonic stem cell (mESC)-to-MN differentiation model, the authors examine Kdm6b's dynamic interactions with stage-specific transcription factors (TFs) to facilitate stepwise MN development.

The results show that Kdm6b drives precise gene activation through dynamic repositioning across the genome-from promoters to distal enhancers-and sequential associations with TFs like Olig2, Ngn2, Isl1, and Cux1. Kdm6b remodels chromatin by removing the H3K27me3 repressive mark and recruiting activation-related histone modifications, such as H3K27ac and H3K4me1. Disruption of Kdm6b activity, either through inhibition or removal, blocks differentiation at specific stages, emphasizing its crucial role in the gene regulatory network underlying MN development.

The study concludes that Kdm6b acts as a key epigenetic regulator, coordinating stepwise MN differentiation in conjunction with stage-specific TFs. The authors refine their previous model by demonstrating how Kdm6b's recruitment by TFs drives its dynamic repositioning and chromatin modification activity. Robust genome-wide data, supported by techniques such as co-immunoprecipitation and IP-MS, validate the proposed model. This work represents significant progress toward understanding Kdm6b's mechanism of action. However, the clarity of data presentation could be improved, and additional experiments might further strengthen the findings. Overall, the study provides a compelling model of Kdm6b's role in MN differentiation and

enriches our understanding of epigenetic regulation during development.

Response: We deeply appreciate the reviewer's comprehensive evaluation. Your recognition for the innovative aspects of our study has been incredibly encouraging and motivating. We are committed to refining the manuscript and addressing the specific concerns raised below to strengthen the study's impact.

Major general comments:

It is my opinion that there are two important experiments to support the.

1) The Kdm6b inhibitor experiment will benefit from some more characterization, at a minimum H3k27me3 Cut&Tag and a description of how it affects MN differentiation. Does it reduce the number of cells transitioning to the next developmental stage? Do the mis-regulated genes maintain H3k27me3? This data will be critical to identify direct GSK-J4 effects vs indirect and build the regulatory model during MN differentiation not with luciferase assays as the authors have all the rest of the data (see below).

Response: We thank the reviewer for the insightful suggestions. We fully agree that a more detailed characterization of the GSKJ4 inhibitor experiment would substantially strengthen our understanding of the role of Kdm6b and its mechanism during MN differentiation. To address these points directly, we performed additional assays in D5 nbMNs. As shown in new Fig 4C-D, immunostaining for Isl1, Lhx3, and Mnx1 revealed a marked reduction in nbMN cell numbers upon GSK-J4 treatment, indicating that inhibition of Kdm6b enzymatic activity impairs the transition of cells to the subsequent developmental stage. Next, we sought to determine how GSKJ4-mediated inhibition remodels histone modifications at downregulated genes as Kdm6b functions primarily as a transcriptional coactivator. This analysis revealed that GSK-J4 treatment leads to a specific increase in H3K27me3 levels at 465 Kdm6b target genes, but not at 353 non-Kdm6b targets that are also downregulated (Fig 4E). These results provide critical mechanistic insight: GSK-J4 directly affects Kdm6b targets through its established role as a demethylase inhibitor, while the downregulation of non-target genes likely represents indirect, downstream consequences.

2) Ideally, the authors will challenge the model to the core by analyzing Kdm6b binding in a TF mutant. This is, however, a more demanding experiment but the one that will cement their claims.

Response: We thank the reviewer for this constructive suggestion. We completely

agree that testing Kdm6b binding in the context of a TF mutant would provide the most direct and compelling evidence for our proposed model. To address this, we employed CRISPR-Cas9 to generate Olig2 knockdown in motor neuron progenitors and examined Kdm6b occupancy at their co-target loci. As shown in new Fig 6C-D, Olig2 knockdown markedly attenuated Kdm6b recruitment to these shared target sites, demonstrating that Kdm6b binding at these sites is dependent on the presence of its associated TF. This finding provides direct evidence that Kdm6b binding specificity is dictated by its interaction with stage-specific TFs, which in turn facilitates the precise temporal regulation of MN differentiation. As the reviewer anticipated, this result significantly cements the mechanistic foundation of our model.

Specific comments:

Overall there is a bit of overclaim in their description of the results and description. I encourage the authors to state claims close to their data.

For example this sentence is not sufficiently supported by the data: "This study revealed Kdm6b recruitment initiates a coordinated histone modification cascade: localized H3K27me3 reduction is accompanied by rapid H3K27ac deposition and H3K4me1 enrichment (H3K27me3↓ / H3K27ac↑ / H3K4me1↑)." They could be independent processes not initiated by Kdm6b. Similarly, the title "Kdm6b dynamically choreographs genomic landscapes of histone modifications" is based solely on correlations not causality.

Response: We sincerely thank the reviewer for this important suggestion. We agree that our initial wording may have implied a stronger causal relationship than is fully supported by the correlative nature of the data. We appreciate your emphasis on precise and cautious language. In the revised manuscript, we have made quite a few of changes to ensure our claims align closely with the evidences. Here, we changed the sentence "This study revealed Kdm6b recruitment initiates a coordinated histone modification cascade…)." into "This study revealed Kdm6b recruitment **coordinated** a histone modification cascade: localized H3K27me3 reduction is accompanied by rapid H3K27ac deposition and H3K4me1 enrichment (H3K27me3↓ / H3K27ac↑ / H3K4me1↑)." Additionally, we changed the title "Kdm6b dynamically choreographs genomic landscapes of histone modifications" into "Kdm6b dynamically **coordinates** genomic landscapes of histone modifications". We believe these changes will strengthen the manuscript by presenting the findings in a more precise and defensible manner. The word “coordinate” presents an integrative role of Kdm6b, not directly initiating or choreographing the processes.

Specific comments related to findings in each figure:

Fig 1: This figure described an already well described method. It is valuable and contextualized the work, but it could be a supplementary figure with EV1 bringing other more novels aspects of this work to the forefront.

Response: We thank the reviewer for this thoughtful suggestion. We acknowledge that the differentiation protocol has been well established in previous work from the Wichterle lab. In the present study, we have extended the characterization of this system by incorporating Ngn2 and Neurod1 immunostaining, enabling a rigorous definition of four distinct stages of motor neuron differentiation. This four-stage framework is essential for understanding the temporal logic that underlies the entire study. As the reviewer noted, Fig 1 provides valuable context for all the following findings from dynamic Kdm6b binding patterns and epigenetic chromatin modifications, to temporal gene regulation and stage-specific TF associations. We believe that presenting this temporal roadmap as a main figure will help readers readily integrate all subsequent findings within this framework. After careful consideration, we prefer to retain Fig 1 as a main figure.

Fig EV5A: It was hard for this reviewer to understand the labels in Fig EV5A and extract any other conclusion than the inhibitor does not produce massive transcriptional changes and D5GSK second is an outliner.

Response: We apologize for the confusion regarding Fig EV5A (new Appendix Fig S1). In this experiment, RNA-seq was performed at four differentiation stages (D4, D4H8, D5, and D6), with three biological replicates per stage. For inhibitor treatment, GSK-J4 was added one day before collection at the indicated stages, also with three replicates. This design enables both the profiling of gene expression during normal differentiation and a direct comparison of transcriptional changes with or without Kdm6b inhibition at matched time points.

The correlation matrix shows pairwise Pearson correlations between all samples. It serves two purposes. First, it demonstrates data quality and reproducibility. The biological replicates within each condition cluster tightly, indicating consistent differentiation and RNA-seq performance. Although the D5_GSKJ4_second replicate shows slightly greater deviation, it still clusters with its group. Second, the matrix reveals distinct transcriptional profiles across differentiation stages.

Regarding the reviewer's observation that GSK-J4 treatment did not induce massive transcriptional changes, we agree that the overall correlation structure does not reflect a global transcriptome remodeling. The 24-hour treatment was intentionally designed to capture direct transcriptional consequences of Kdm6b inhibition, thereby

enriching for sensitive primary targets rather than widespread secondary effects, as shown in Fig 4B. This figure has now been moved to the Appendix as a reference for data quality and experimental design.

Fig 2C: Just a note of caution for the authors. Cut&Tag has a well-recognized bias towards accessible regions, thus proximal regions and TSS, specially in early differentiation states. I am wondering if this is what skewing some of the early proximal vs late distal and it is a gain of Kdm6b binding in regulatory regions as as cells differentiate. An equally or more exciting pattern.

Response: We thank the reviewer for this important technical insight regarding CUT&Tag's potential bias toward accessible chromatin. We agree that the inherent bias of Cut&Tag towards open chromatin is an important consideration. However, to detect Kdm6b binding on the genome, we employed Cut&Run assay which employs Mnase and has much lower bias towards accessible regions compared to Cut&Tag. This methodological choice gives us confidence that the dynamic binding patterns we observe reflect genuine biological changes rather than technical artifacts.

More importantly, we find the reviewer's alternative interpretation "a gain of Kdm6b binding in regulatory regions as cells differentiate" to be both insightful and biologically compelling. Re-examining our data revealed an important pattern we had previously overlooked. As shown in Fig 2B, Kdm6b binding sites at proximal promoter regions remain stable in absolute number across differentiation (D4H8: 5067 sites, D5: 5077 sites, D6: 5072 sites) despite a proportional decrease. In contrast, binding sites at intronic and intergenic regions increases progressively: from 707 sites (33.12% of total) at D4 to 3879 (45.38%) at D4H8, 6524 (51.42%) at D5, and 8152 (57.62%) at D6. **These numbers reveal that the dynamic redistribution of Kdm6b during differentiation is not driven by loss from promoters, but rather by a progressive gain of new binding events at distal regulatory elements.** Based on this insight, we have revised our manuscript accordingly. Rather than describing a "shift" from proximal to distal regions, we now frame our central conclusion as a progressive gain of Kdm6b binding at distal regulatory elements during differentiation. We are grateful to the reviewer for guiding us toward this more compelling interpretation.

Fig 3B: the number of peptides, biological replicates and statistics needs more clarification. Is 1 peptide considered enrichment?

Response: We thank the reviewer for raising these concerns about our IP-MS data

shown in Fig 3B (new Fig 5B). Fig 5B represents peptide numbers identified from a single IP-MS experiment. To confirm the reproducibility of these findings, we performed a second biologically independent replicate, which is shown in Fig EV5A. Both replicates showed highly consistent patterns, with comparable peptide numbers for each TF across differentiation stages. Because we have only two replicates, we did not perform statistical testing.

Regarding the specific question about whether a single peptide constitutes evidence of enrichment, we fully acknowledge that a single peptide should be interpreted with caution. However, a single peptide for Sox2 was identified in both replicates at D4H8. Moreover, we were able to validate their interaction by both co-IP (Fig 5C) and their genomic co-occupancy (Fig 6B). Thus, these multiple lines of evidence collectively support Sox2 as a bona fide Kdm6b-interacting partner in motor neuron progenitors. We hope this clarification addresses the reviewer's concerns.

Fig 3F: are the numbers on then Venn diagram correct? It seems the total number o sites is represented as exclusive.

Response: We are sorry for this mistake. We have already corrected the numbers as exclusively represented in new Fig 6B.

Fig 4: I am sure I missed it, but the number of biological replicates and how it was quantified was not very obvius to me.

Response: We thank the reviewer for pointing out these issues. For the epigenomic datasets presented in this figure (new Fig 3), including Kdm6b CUT&RUN and H3K27me3/H3K27ac/H3K4me1 CUT&Tag, each represents a single biological experiment. We acknowledge that biological replicates would further strengthen the conclusions, and this limitation is noted in the revised Methods section. To enable quantitative comparison across samples and stages, we used the same number of cells for each assay and normalized read counts from binding regions to sequencing depth, expressed as Reads Per Million mapped reads (RPM: region reads / total mapped reads × 10^6). This normalization minimizes batch effects and allows relative binding intensity to be compared across samples. We have now clarified this information in the Methods section.

Fig 4: This is a complex figure that to me supports Fig 2 because as far as I can tell

prescind of any TF association data.

Response: We thank the reviewer for this observation. We would like to clarify the distinct purposes of these figures. Fig 2 focuses on Kdm6b binding dynamics during differentiation. In contrast, Fig 4 (new Fig 3) examines the functional consequences of this binding, specifically reflecting how Kdm6b occupancy shapes dynamic histone modifications. As the reviewer correctly notes, this figure does not yet incorporate TF mechanisms. Following this logic, we have reorganized the manuscript to first present Fig 3, followed by the underlying TF association mechanism. We thank the reviewer for prompting this clarification.

Fig 4B: Since the authors might want to highlight the dynamics, would a "subway map" or any other graphic from to highlight transition would be a better representation? I tis hard to understand the meaning and extract information in its current form.

Response: We thank the reviewer for this helpful suggestion regarding data visualization in Fig 4B (new Fig 3B). We agree that it is important to clearly convey dynamic changes. We have tried alternative representations, including heatmap plot, but found that the current line plot most effectively conveys the temporal dynamics we wish to highlight. Before reclarification, let's explain Fig 3A, in which Kdm6b binding peaks across four differentiation stages D4, D4H8, D5 and D6 are grouped into 15 categories: unique to single stage (D4, D4H8, D5 and D6), unique to two stages (D4D4H8, D4D5, D4D6, D4H8D5, D4H8D6, D5D6), shared by three stages (D4D4H8D5, D4D4H8D6, D4D5D6, D4H8D5D6) and shared by four stages (D4D4H8D5D6). These 15 categories represent different types of Kdm6b binding peaks. To examine the relationship between Kdm6b binding and chromatin stages, we focused on seven biologically relevant types of specific Kdm6b binding peaks: unique to single stage (D4, D4H8, D5 and D6) or unique to two consecutive stages (D4D4H8, D4H8D5 and D5D6). Fig 3B then tracks the enrichment of histone modifications (H3K27me3, H3K27ac and H3K4me1) at these seven peak sets across all four time points with different color lines. For example, at D4_unique peaks, H3K27me3 levels remain low at differentiation stages D4 and D4H8, then progressively increases at D5 and D6. Conversely, H3K27ac and H3K4me1 show the opposite pattern: high at stages D4 and D4H8 and then gradually declining at D5 and D6. Therefore, Kdm6b binding is associated with low H3K27me3 and high H3K27ac/H3K4me1 levels. Upon Kdm6b releasing from D4_unique peaks at D4H8, D5 and D6 stages, H3K27me3 gradually increases while H3K27ac and H3K4me1 decrease. The same principal applies for other peak categories. **This dynamic coordination between Kdm6b occupancy and histone modification landscapes**

is the central message of Fig 3B, and we believe the current format effectively illustrates this finding. We hope this explanation clarifies the rationale behind our presentation.

Fig 5: This result section presented significant challenges for this reviewer. It was hard to extract what the authors wanted to highlight. What is te meaning/value of GO terms? What is here that is not expected from what we know of gene expression and Kdm6b activity? I left agreeing with this statement that I broad and perhaps unnecessary: "These findings collectively indicate that the transcriptional network governing MN differentiation is highly complicated, crucially but not solely dictated by Kdm6b binding."

Response: We thank the reviewer for raising these important points. We acknowledge that the original Fig 5 (new Fig EV4) was overly complicated and its main message was not clearly conveyed. **The purpose of this figure was to provide an overview of how Kdm6b target genes are transcriptionally changed during differentiation.** The key observation is that most early-bound targets are downregulated over time, whereas a majority of later-bound targets are progressively upregulated. The selected GO terms are included simply to illustrate that these gene cohorts are enriched for biological processes relevant to the respective developmental stages, consistent with expectations given Kdm6b's role in temporally regulating differentiation. While we did observe some complexity in the transcriptional patterns of certain Kdm6b target genes, this was not the main point we intended to convey. To improve clarity, we have removed the more complex expression analyses and retained only the core trends in the revised manuscript. We also removed the sentence “These findings collectively indicate that the transcriptional network…”. We believe this version more effectively communicates the intended message. Thank the reviewer for helping us improve the clarity and focus of the manuscript.

Fig 6: the perturbation of the system is welcomed. Have the authors show that the effect of GSK-J4 on chromatin modifications? The results support the know Kdm6b role in promoting differentiation. Perhaps the interesting and novel aspect of this work is correlating the transcriptional changes with Kdm6b binding and TF occupancy which I could not easily extract from any of the figures.

There is an abrupt jump into this conclusion without the proper comparisons: "Thereby, these findings demonstrate that Kdm6b enzyme activity for removing H3K27me3 is required for the timed activation of co-targets of Kdm6b-TF and strongly support a

model where Kdm6b collaborated with stage-specific TFs to precisely regulate gene expression programs to drive stepwise differentiation."
I value the revisionist experiments n Fig 6B, but the authors have the data to do it genome wide during MN differentiation to support their point.
Perhaps reorganizing the figures with Fig 3 and Fig 6B as final figure would allow the authors to craft the importance of Kdm6b dynamics, ifs phenotype and end with the mechanisms behind Kdm6b dynamics recruited by TFs.

Response: We thank the reviewer for these insightful suggestions and comments. We fully agree with the reviewer that the novelty and persuasiveness of this study should center on correlating Kdm6b binding dynamics with transcriptional changes and its association with TFs. We also accept that the original conclusion ("Thereby, these findings demonstrate that Kdm6b enzyme activity...") was abrupt and indirectly supported by the data presented. Based on this valuable feedback, we have substantially reorganized the manuscript and supplemented our data. Our specific responses are as follows.

We have adopted the reviewer's proposal to restructure the logical flow of the manuscript and reorganized the figures. The new narrative now begins with Kdm6b's own binding dynamics (Fig 2) and how it establishes an adaptable chromatin landscape (Fig 3), then proceeds to its role in regulating temporal gene expression (Fig 4), and finally culminates in the mechanism of its collaboration with stage-specific TFs (Fig 5 and 6). We believe this progression, from “function” to “mechanism”, more clearly illustrates Kdm6b’s central role in achieving temporal precision during MN differentiation.

Regarding new Fig 4, we have focused our demonstration on the necessity of Kdm6b’s role for the timely activation of genes using the GSK-J4 inhibitor, since it is hard for us to establish Doxcycline-inducible Kdm6b knockdown at different differentiation stages in the revision window. We found that many downregulated genes upon inhibition of Kdm6b activity are enriched for those critical for stepwise MN differentiation. This provides strong correlative evidence for Kdm6b's role in temporal control. Addressing the reviewer's specific query about “the effect of GSK-J4 on chromatin modifications”, we have added a crucial experiment in Fig 4E. We demonstrate that GSK-J4 treatment specifically increases H3K27me3 levels at Kdm6b target genes (465 downregulated genes by GSKJ4 treatment at D5), but not at non-Kdm6b target genes (353 downregulated genes by GSKJ4 treatment at D5). This result supports that GSK-J4's direct effects are mediated through its action on Kdm6b's targets, strengthening the specificity of the observed phenotype.

In the new manuscript, we have removed the previously criticized conclusion and

replaced it with a more measured statement that is directly supported by our data: "Collectively, these findings demonstrate that Kdm6b directs the sequential activation of temporally regulated genes, thus enabling stepwise MN differentiation."

In summary, by reorganizing the figures and refining our conclusions, we have significantly strengthened the logical chain of our demonstration. The revised manuscript now better integrates Kdm6b binding, its impact on chromatin and transcription, and its mechanistic cooperation with TFs into a coherent story that highlights how Kdm6b achieves temporal precision in MN development.

Fig 6C: It is hard for me to understand the value of these experiments in the way they are presented.

Response: We agree with the reviewer's assessment. Upon re-evaluation, we have removed Fig 6C in the revised manuscript accordingly, since this experiment did not substantially contribute to our core conclusions.

Fig 7: There is a typo in Neurod1

Response: We are sorry for this error and we have corrected it. Thank you.

Minor: I noted the punctuation sometimes is on the wrong side of the citations.

Response: We are sorry for this error. We have checked all the citations and corrected them. Thank you.

Referee #2:

The Study by Ren et al. follows up previous work from Wang et al 2022 describing the role of Kdm6b in driving MN development, which they had previously shown in vivo. To study the specific targets of Kdm6b and how it finds these targets, the authors leverage the use an mESC to MN protocol that has been previously published to perform geneomics experiments and demonstrate sequential co-binding to different TFs during MN development. The authors additionally validate this through a series of elegant luciferase reporter assays. Collectively, the study provides insights into mouse MN development and the role of Kdm6b. The authors collect valuable and

unique datasets, however, the presentation of the data could be clarified for better comprehension.

Response: We sincerely thank the reviewer for this perceptive and encouraging summary of our work. We appreciate the recognition of our unique datasets and the elegant validation experiments. We also acknowledge the constructive critique regarding data presentation. In the revised manuscript, we have carefully addressed the specific concerns to improve clarity and comprehension, as detailed in our point-by-point response below.

Major commment:
Figures - in general the figure legends need more descriptive to give the reader more direction on how to interpet the data and include the technical or biological n used, the transtical test being used etc. Many of the genomics graphs are missing scales and information about what type of metric is being plotted and how it is normalized. Some specific points and questions are precised below.

Response: We thank the reviewer for raising this important point. We have thoroughly revised all figure legends to be more descriptive and better understandable. Each legend now includes: (1) the number of biological or technical replicates if n > 1, (2) the statistical tests used and their corresponding p-value thresholds, (3) clear definitions of the metrics being plotted, (4) normalization methods for genomics data, and (5) Scales have been added or clarified. We believe these revisions will significantly improve the reader's ability to interpret the data.

Figure 1:
• I was personally missing the expression of Kdm6b at the different stages of the differentation, and potentially also the TFs that will then be important for the dyanmic changes later on.

Response: We thank the reviewer for this helpful observation. We agree that showing Kdm6b and key TF expression across stages provides important context. In response, we have included these data in Fig EV5D, which displays the average expression levels of Kdm6b and relevant TFs from three biological replicates of RNA-seq at each differentiation stage. Their expression patterns are consistent with the immunostaining results in Fig 1.

Figure 2
• 2A - add a scale bar to the CUT&TAG enrichment plot.

Response: We thank the reviewer for this suggestion. In Fig 2A, the Y-axis denotes individual Kdm6b binding sites identified at each stage, and the X-axis represents the

differentiation time course over which Kdm6b occupancy was measured. **The color bar at the bottom indicates Kdm6b enrichment intensity, serving as the scale for signal strength.** We have clarified this in the revised figure legend.

Figure 3:

• In panel E, at which time point is the Neurod1 chip performed?

Response: We thank the reviewer for this question. The Neurod1 CUT&RUN assay was performed at the D5 stage. In the revised manuscript, this figure has been reorganized as Fig 6A, and this information is now indicated in the updated figure legend.

Figure 4:

• I find panel A hard to read and hard to interpret. The final message is that there are many overlapping sites later on? Numbers are hard and small to read.

Response: We thank the reviewer for pointing out the readability issues with this panel. In original Fig 4A (new Fig 3A), Kdm6b binding peaks across four differentiation stages D4, D4H8, D5 and D6 are grouped into 15 categories: unique to single stage (D4, D4H8, D5 and D6), unique to two stages (D4D4H8, D4D5, D4D6, D4H8D5, D4H8D6, D5D6), shared by three stages (D4D4H8D5, D4D4H8D6, D4D5D6, D4H8D5D6) and shared by four stages (D4D4H8D5D6). These 15 categories represent different types of Kdm6b binding peaks. While many sites are shared across stages, a substantial number of stage-specific peaks also exist. For instance, 6,144 peaks are unique to D6, representing nearly half of all binding sites at that stage. The purpose of this panel is to identify these unique and dynamic binding events, which then serve as the foundation for analyzing how Kdm6b remodels chromatin landscapes during differentiation (Fig 3B). To improve clarity, we have increased the font size for the numbers in the revised figure. We thank the reviewer for this helpful suggestion.

Figure 5:

• In the RNA-expression data, I am a little bit lost following the analysis. For a gene to be considered up or down regulated, at how many time points does it need to be going up or down?

Response: We thank the reviewer for this question and acknowledge that the analysis in the original Fig 5 (new Fig EV4) was not clearly explained. For each set of Kdm6b target genes, we assessed their expression dynamics by performing pairwise comparisons between specific stages, specifically indicated as below.

For common target genes (Com): D6 vs. D4

For D4-Com genes: D6 vs. D4

For D4H8-Com genes: D6 vs. D4H8

For D5-Com genes: D5 vs. D4

For D6-Com genes: D6 vs. D4

Genes with fold change ≥ 1.1 were considered upregulated, ≤ 0.9 downregulated, and between 0.9 and 1.1 as constant expression. This approach allowed us to capture whether genes bound at a given stage are activated or repressed as differentiation proceeds. The goal was simply to provide an overview that Kdm6b targets are temporally regulated in accordance with their developmental functions. We have now clarified these criteria in the Methods section.

• How are these graphs scaled? Do the number represent TPMs, RPKM etc? Again, as with all the other figures, more details need to be included in the legend so that they are easy to interpret.

Response: We thank the reviewer for this concern. Gene expression levels are reported as FPKM (Fragments Per Kilobase of transcript per Million mapped fragments), which normalizes for both sequencing depth and transcript length, enabling comparison across samples. We have now explicitly stated this in both the figure legend and the RNA-seq analysis section of the Methods. We agree that detailed legends are essential for data interpretation; accordingly, we have revised all figure legends to include relevant normalization information, scaling, and statistical details.

• What is the scale on the box plots?

Response: Thank the reviewer for this question. We used FPKM values to qualify gene expression levels, as described above. The scale on the box plots is Log_2(FPKM) value.

• I frankly have a difficult time interpreting the main message of this figure.

Response: We thank the reviewer for raising this concern. We acknowledge that the original Fig 5 (new Appendix Fig S1) was overly complicated and its main message was not clearly conveyed. **The purpose of this figure was simply to provide an overview of how Kdm6b target genes are transcriptionally regulated during differentiation.** The key observation is that most early-bound targets are downregulated over time, whereas a majority of later-bound targets are progressively upregulated. The selected GO terms are included only to illustrate that these gene cohorts are enriched for biological processes relevant to the corresponding

developmental stages, consistent with Kdm6b's established role in temporally regulating differentiation. While we did observe some complexity in the transcriptional patterns of certain Kdm6b target genes, this was not the main point we intended to convey. To improve clarity, we have removed the more complex expression analyses and retained only the core trends in the revised manuscript. We believe this version now conveys the intended message more effectively.

Figure 6:

• The authors miss the opportunity to compare the overlap between genes whose expression is altered after the GSK treatment and the different Kdm6b targets. This is given as the supplementary data, but could be nicely represented alongside the RNA-seq data, which alone is less interesting in this context.

Response: We thank the reviewer for this excellent suggestion. We agree that directly comparing GSKJ4-affected genes with Kdm6b target genes provides a more compelling representation. Because Kdm6b functions primarily as a transcriptional coactivator, we focused our analysis on genes downregulated upon GSK-J4 treatment. The overlap between these downregulated genes and stage-specific Kdm6b targets is now shown as Venn diagrams in Fig 4A. We believe this presentation effectively complements the RNA-seq data and highlights the direct targets of Kdm6b activity.

In general, I think it's a bit difficult to compare the trends as a total number of binding sites, because these change between the different time points. I think it would be more intuitive to express overlaps between Kdm6b and the TFs as a percentage of all possible (i.e. profiled) Kdm6b sites.

Response: We thank the review for these great suggestions. We agree that comparing absolute binding site numbers across time points can be challenging. In consistence, we have already employed two complementary strategies: (1) stage-specific binding sites for analyzing gene expression and associated functions (Fig 2F, G and Fig EV4), and (2) unique binding sites for examining histone modification dynamics (Fig 3). Additionally, following the reviewer's recommendation, we now also present the overlap between Kdm6b and TF binding sites as a percentage of total Kdm6b sites at each stage (Fig 6B). We believe this representation will significantly improve the clarity and interpretability of our findings.

Using the mass-spec experiment described, and the previous in vivo data, do the authors observe Kdm6a expression in the MN cultures? This could be worth discussion as it is equally inhibited by GSK-J4.

Response: We thank the reviewer for this insightful consideration. Based on our

RNA-seq data from three biological replicates across differentiation stages, Kdm6b expression is progressively upregulated during MN differentiation, whereas Kdm6a expression remains low and slightly decreases in post-mitotic neurons in the following Response Fig 1. These expression patterns are consistent with our previous in vivo study in mouse spinal cords (Wang *et al*, 2022). Furthermore, we previously showed that conditional knockout of Kdm6a in vivo does not impair MN differentiation (Wang *et al.*, 2022). Importantly, the GSK-J4 concentrations used in our experiments (1-5 μM) are well below the range reported to inhibit Kdm6a (>10 μM). Taken together, we conclude that Kdm6b is the primary target of GSK-J4 mediating the effects on MN differentiation observed in this study. We hope this clarification addresses the reviewer's concern.

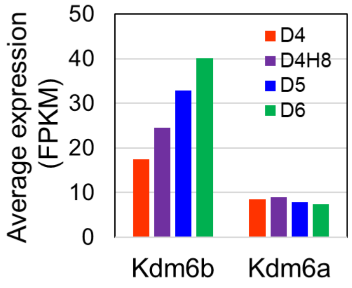

Response Fig 1. Kdm6b and Kdm6a expression during MN differentiation. Average expression levels were determined by RNA-seq at stages D4, D4H8, D5, and D6. n=3 biological replicates per stage.

The authors note that " Notably, a fraction of key MN genes (e.g., Pbx3 in Fig EV2A and Prox1 in Fig EV5D) were subject to dual regulation through both common and stage-specific binding sites, unveiling the utilization of multiple binding modalities for exquisite expression control." Is there a different in the level of expression in these genes based on the presence of additional stage-specific binding sites at certain time points?

Response: We thank the reviewer for this thoughtful question. To address this, we examined the expression levels of Prox1 and Pbx3, two genes highlighted as having multiple binding modalities. Prox1, which exhibits the highest number of Kdm6b binding peaks at D4H8 (Fig EV4F), shows high expression at D4H8 and D5, followed by a decrease at D6 (Fig EV4G). In contrast, Pbx3, which gains increasing Kdm6b binding peaks as differentiation progresses (Fig EV2A), shows correspondingly progressive upregulation. These data demonstrate that the presence of additional stage-specific binding sites largely correlates with enhanced expression of target

genes. We thank the reviewer for prompting this analysis, which strengthens our conclusion that multiple binding modalities enable precise temporal control of gene expression.

Methods/techniques:

1) What kit was used (if any) was used for library consturction. What were the sequencing parameters? What parameters were used in Deseq2 to designate differeentially expressed genes beyond the FC (i.e. p-adj. value, SE etc)? Did you perform additional correction to look at the different time points in your series?

Response: We thank the reviewer for these technical questions. For CUT&RUN-seq, library construction was performed using the Hyperactive pG-MNase Cut&Run Assay Kit for Illumina (Vazyme HD102, China). For CUT&Tag-seq, we used the Hyperactive Universal Cut&Tag Assay Kit for Illumina Pro (Vazyme TD904, China). For RNA-seq, mRNA was purified using poly-T oligo-attached magnetic beads, and library construction was conducted by Novogene (China). All sequencing was performed on an Illumina platform with paired-end 150 bp reads and a depth of 6G raw data per sample. For differential expression analysis using DESeq2, genes with p-value < 0.05 were considered significantly changed. To enable comparison between samples, we normalized CUT&RUN and CUT&Tag data using RPM (Reads Per Million mapped reads) and RNA-seq data using FPKM (Fragments Per Kilobase of transcript per Million mapped fragments). The same cell numbers were used for each experiment type to minimize batch effects. All relevant details have now been included in the updated Methods section. We thank the reviewer for helping us improve the completeness of our technical reporting.

2) To assess the specificity of the antibodies and the library quality, was a IgG only negative control reaction performed for CUT&TAG experiments and Chip-seq experiment?

Response: We thank the reviewer for this technical question regarding antibody specificity and negative controls. For conventional ChIP-seq, IgG negative controls are standard practice. However, for CUT&RUN and CUT&Tag assays, the signal-to-noise ratio is exceptionally high with minimal background, making IgG controls less critical and not routinely performed in the field. Nevertheless, we agree that an IgG control could provide additional confidence. In our study, we focused on stage-specific binding differences of Kdm6b and histone modifications across time points. By comparing enrichment patterns across stages, intrinsic background signals, if present, would be largely subtracted out, as they would be consistent across samples. We thank the reviewer for the opportunity to clarify this point.

This is more of a philosophical point, but the title of the paper is Kdm6b-mediated choreography, however, in fact that what authors show is that Kdm6b is a constant act and is being choreographed by the presence of different TFs that appear and disappear over the course of the differentation timeline. Thus, I think it would be more appropriate to turn the title to something like "Temporable Transcription Factor Expression Guides Kdm6b-mediated epigenome remodeling during motor neuron differentiation"

Response: We thank the reviewer for this thoughtful and philosophical point. We find the reviewer's perspective insightful: rather than Kdm6b itself choreographing the process, it is being dynamically guided by temporally expressed transcription factors that appear and disappear over the differentiation timeline. This framing accurately captures the hierarchical relationship revealed by our findings. We carefully considered the reviewer's suggested title: "Temporal Transcription Factor Expression Guides Kdm6b-mediated Epigenome Remodeling during Motor Neuron Differentiation." However, given that we lack extensive histone modification analyses in multiple TF-mutant MNs across multiple developmental stages, this title may be challenged. Moreover, Kdm6b remains the central focus of our study which provides a compelling model of Kdm6b's role and its epigenetic regulation in a stage-specific manner. We sought a title that maintains Kdm6b as the subject while acknowledging its guidance by TFs. After deliberation, we have revised the title to: "**Kdm6b-mediated epigenetic coordination of temporal precision during motor neuron differentiation**." The term "coordination" captures Kdm6b's role as an integrative node that translates TF inputs into epigenetic outputs, thereby achieving temporal precision. This formulation preserves Kdm6b as the central player while implicitly acknowledging that its actions are guided by stage-specific TFs. We believe this strikes an appropriate balance and accurately reflects the model proposed in our study. We thank the reviewer for helping us refine this important aspect of the manuscript.

Minor points:

• Names of genes are incorrectly formatted (italics etc), upper case for proteins.

Response: We thank the reviewer for identifying these formatting errors. In the revised manuscript, we have italicized the gene names and capitalized the first letter of all protein names according to standard nomenclature for mouse genes and proteins.

Referee #3:

In the manuscript by Ren et al., the authors investigate the role of the histone lysine demethylase Kdm6b during the directed differentiation of mouse ESC into motor neurons. The authors propose a model whereby the sequential association of Kdm6b with stage-specific TFs instruct the temporal onset of developmental programs and the overall progression towards terminal differentiation and maturation. This work, expand on previous work from the same laboratory (Wang et al., 2022) that identified a role for Kdm6b in regulating the specification of motor neuron subtypes via functional interaction with the motor neurons -specific TFs Isl1-Lhx3.
The dynamic regulation of H3K27 (de)methylation for setting timing during neural differentiation and maturation and the contribution of kdm6b in this process has been already reported in vitro and in vitro and across distinct neuron types. This manuscript introduces a further layer of molecular complexity providing a model on how the regulation of the epigenetic landscape during neural differentiation is achieved with fidelity and cell-type specificity (i.e., association with Kdm6b with TFs). These finding are of potential interest for the field. However, there are a series of concerns that would need to be addressed. I elaborate on the main points below.

Response: We thank the reviewer for the thoughtful and constructive evaluation of our manuscript. We appreciate the recognition that our findings introduce an additional layer of molecular complexity to understanding how epigenetic regulation achieves temporal precision during neural differentiation. We agree that addressing the raised concerns will strengthen the study. Below, we provide a point-by-point response to the reviewer's comments.

- In Fig. 1, the authors provide a characterization of the expression of key markers during mouse ESC motor neuron differentiation. Another example is provided in Fig. EV1. The images convey well the partially overlapping and subsequent expression of key TF. However, a quantitative assessment is missing. Given the importance of the experimental platform for capturing discreet developmental stages based on the timed expression of these TFs, the authors should provide evidence for the robustness and reproducibility of their differentiation method.

Response: We thank the reviewer for this important suggestion. We agree that demonstrating the robustness and reproducibility of the differentiation platform is essential, given its central role in defining discrete developmental stages. While we did not perform quantitative assessment of the immunostaining shown in Fig 1B, we provide two complementary lines of evidence supporting the reliability of our staging strategy. First, this differentiation protocol is well established and has been

extensively validated in previous studies, which we have cited in the manuscript accordingly. Second, and more directly, we performed three biologically independent differentiation experiments and collected samples for RNA-seq at each stage. As shown in Appendix Fig S1, pairwise Pearson correlation analysis of transcriptional profiles revealed excellent reproducibility among replicates at all four stages (D4, D4H8, D5, D6), confirming the robustness of the differentiation method. Additionally, mRNA expression levels of key transcription factors (Fig EV5D) closely mirror the immunostaining patterns observed in Fig 1B, providing further validation. We hope these data collectively address the reviewer's concern.

- The authors performed Kdm6b CUT&RUN during motor neurons differentiation and conclude that Kdm6b occupancy at specific genomic loci is stage specific. However, the tornado plots in Fig. 2A, show a gradual increase in Kdm6b occupancy during the differentiation, rather than discreet, stage -specific patterns of binding to gene subsets. Similar findings are reported in Fig. 5, where two major trends of gene expression (gradual up and down -regulation) dominate the dataset and subsequent transcriptional programs are not observed. At the light of these observations, the authors findings indicate that Kdm6b may regulated a 2-stage process: proliferation vs. post-mitotic maturation. This is consistent with axonogenesis and synapse organization as the most enriched GO for putative Kdm6b targets at later stages (Fig. 2G). In absence of discreet signatures downstream of stage specific TFs is difficult to assess whether Kdm6b control the temporal progression/directionality of a developmental process in a stepwise manner. The authors could further analyze the RNAseq data (Fig. 5) to test whether such discreet stages are captured in their experimental system and, if present, use the associated transcriptional signatures for the subsequent analysis.

Response: We thank the reviewer for these insightful comments. We agree that the gradual increase in Kdm6b occupancy shown in Fig 2A, which includes both common and stage-specific binding sites, does not by itself reveal noticeably discrete stage-specific patterns. However, when we separate these categories as shown in Fig 2F and Fig 3A, a large number of stage-specific Kdm6b binding sites become evident, indicating its dynamic and stage-specific occupancy. Regarding gene expression, we acknowledge that the overview in old Fig 5 (new Fig EV5) shows two major trends: early downregulation and late upregulation. However, the early downregulated genes at D4 and D4H8 are largely distinct, as are the late upregulated genes at D5 and D6. Further analysis of Kdm6b target genes across all four stages (D4, D4H8, D5, D6) reveals stage-specific gene sets, with many unique targets identified at each stage in the following Response Fig 2. Moreover, we find that stage-specific TFs associate

with Kdm6b to drive its dynamic binding and confer target gene specificity (Fig 5 and Fig 6). The stage-specific target genes are selectively mis-regulated upon GSK-J4 treatment at corresponding stages (Fig 4B, Dataset EV3-5). Together, these observations support a model in which Kdm6b, in association with temporal TFs, dynamically binds to specific regulatory elements and temporally regulates specific sets of target genes to drive stepwise MN differentiation. We thank the reviewer for the opportunity to clarify this important concern.

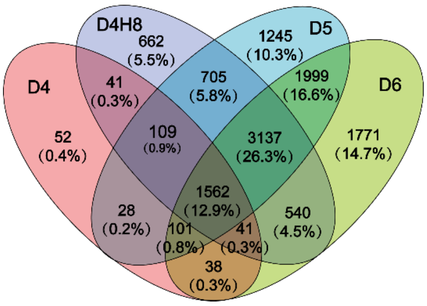

Response Fig 2. Comparison of Kdm6b target genes across four differentiation stages. Numbers indicate gene counts and percentages represent the proportion of each gene set relative to the total number of Kdm6b targets.

- The progressive shift in Kdm6b occupancy at intergenic regions versus promoters is clearer. This might suggest that kdm6b at later stages is associated with activity-dependent TFs/programs, which primarily act at enhancer sequences. This interpretation would be consistent with the expected increase in neuronal activity at later stages. I wonder whether the authors could address this and expand the analysis in Fig. 3A by performing and unbiasedly show additional tests of TF motif enrichment.

Response: We thank the reviewer for this insightful suggestion. We agree that the progressive gain of Kdm6b occupancy toward distal regions raises the possibility that Kdm6b may cooperate with activity-dependent TFs at later stages. To address this, we performed unbiased motif enrichment analysis on both common biding sites (Com) and stage-specific binding sites (D4-Com, D4H8-Com, D5-Com and D6-Com). The full results are provided in Dataset EV1. At D6, which represents an early maturation stage, we did not observe enrichment of activity-dependent TF motifs (e.g., Mef2, AP-1) among the top 100 motifs. This is consistent with a recent study showing that such motifs become prominent only at later maturation stages (Patel *et al*, 2022). In Fig 5A, we prioritized TFs that appeared among the top 20 enriched motifs and were also validated as Kdm6b interactors by IP-MS or co-IP. For motifs corresponding to TFs not detected in our interaction assays, the full enrichment data remain available in Dataset EV1.

- In Fig. 3F, it would be informative to assess the nature/known function of the genes co-enriched and non-coenriched between Kdm6b and Sox2, Ngn2, Olig2, Isl1-Lhx3, Onecut1, Cux.

Response: We thank the reviewer for this suggestion. Following the reviewer's suggestion, we did GO analyses of the genes co-enriched and non-coenriched between Kdm6b and Sox2, Olig2, Ngn2, Neurod1, Isl1-Lhx3, Onecut1 and Cux in old Fig 3F (new Fig 6B). The top 10 enriched biological process (BP) terms for each category are now provided in Dataset EV2, which would be informative to assess their respective known functions, as the reviewer noted. The genes associated with co-enriched and non-coenriched sites between Kdm6b and TFs are involved in distinct biological processes, suggesting specific roles of Kdm6b-TF partnerships in regulating MN development. This is updated in the revised manuscript.

- The line plots in Fig 4B show a largely inverse correlation between H3K27me3 and H3K27ac and H3K4me1, especially in the four columns on the left. This is consistent with the known association of such markers with gene repression versus activation respectively and with the role of Kdm6b as an H3K27 demethylases. However, the conclusion that Kdm6b is the master regulator of those patterns is not directly supported by the data. Most of the plots show very subtle differences which makes their interpretation challenging. Furthermore, functional experiments (e.g., CUT&RUN upon Kdm6b inhibition) are missing.

Response: We thank the reviewer for this thoughtful critique. As noted, we observed an inverse correlation between H3K27me3 and H3K27ac/H3K4me1 in old Fig 4B (new Fig 3B), where Kdm6b binding and release are accompanied by dynamic chromatin remodeling. Importantly, this conclusion is based on the aggregate analysis across all stage-specific binding sites (Fig 3B) and is not dependent on the selected individual loci shown in Fig 3C–G, where subtle differences are expected given locus-specific variation. The central question is whether this inverse correlation is directly or indirectly driven by Kdm6b. Our IP-MS data (Fig EV3D) reveal that Kdm6b associates with EP300 (H3K27 acetyltransferase) and Kmt2c/2d (H3K4 methyltransferases), suggesting that Kdm6b functions not only as an H3K27 demethylase but also as a cofactor coordinating broader histone modification networks. We agree that correlative data alone do not establish Kdm6b as the master regulator of these patterns, and we acknowledge the need for functional evidence. However, establishing inducible Kdm6b knockdown cells for time-resolved analysis

across differentiation stages is really hard for us within the revision timeline. We instead performed GSK-J4 inhibition experiments in D5 nbMNs. As shown in Fig 4D, acute Kdm6b inhibition led to increased H3K27me3 and decreased H3K4me1 at Kdm6b target genes downregulated by GSKJ4 treatment, directly supporting its role in modulating these marks. Notably, H3K27ac levels were minimally affected by GSK-J4 treatment, contrasting with the decreased H3K27ac observed upon natural Kdm6b dissociation (Fig 3B). This suggests that the H3K27ac dynamics associated with Kdm6b occupancy may be mediated through its interaction with EP300, independent of its demethylase activity. Based on these data, we conclude that Kdm6b serves as **a critical coordinator of chromatin landscapes** during MN differentiation. Accordingly, we have revised the subtitle to "Kdm6b dynamically coordinates genomic landscapes of histone modifications" to more accurately reflect the evidence. We thank the reviewer for helping us refine this interpretation.

- The functional experiments shown in Fig. 6 are interesting, as they suggest that TFs guide Kdm6b to specific chromatin loci to remove the H3K27me3 epigenetic repression and thereby achieve full gene activation. Would the forced overexpression of late TFs in Kdm6b knock out mouse ESC (or upon Kdm6b inhibition) prevent the programming of ESC into motor neurons?

Response: We thank the reviewer for this insightful suggestion and are encouraged by the positive recognition of our findings (new Fig 5 and Fig 6). We fully agree that forced expression of late-stage TFs in a Kdm6b-deficient background would provide a powerful test of whether Kdm6b is required for TF-mediated activation of the MN program. We acknowledge that establishing doxycycline-inducible TF overexpression or inducible Kdm6b knockdown ESC lines for such experiment was not feasible within the revision timeline. As an alternative approach, we performed in vivo chick electroporation experiments. Co-electroporation of Isl1 and Lhx3 with or without Kdm6b shRNA into the developing chick spinal cord revealed that Kdm6b knockdown significantly attenuated Isl1-Lhx3-mediated induction of Mnx1 (Fig 6F, G). This result demonstrates that Kdm6b is required for TF-driven activation of MN genes, consistent with the model that TFs recruit Kdm6b to target loci for full gene activation. We hope this functional evidence helps address the reviewer's question.

- The authors proposed a mechanism by which a promoter-to-enhancer shift in Kdm6b occupancy orchestrates timed gene expression during motor neuron differentiation. Previous reports indicate that the dynamics of resolution of chromatin bivalency at promoters of developmental genes is also a key regulator of the timing of

developmental progression. While those two models are rather complementary that in contrast, I feel it would be important to assess chromatin bivalency and its dynamics in the experimental system used in the manuscript, in addition to discuss more broadly the two models in the discussion.

Response: We thank the reviewer for this insightful suggestion. We fully agree that the resolution of chromatin bivalency at promoters of developmental genes represents another key mechanism for regulating the timing of developmental progression, and that the two models are complementary. In response, we analyzed histone modifications at promoter and enhancer regions occupied by Kdm6b at each stage (Fig EV2B). Notably, while H3K27me3 and H3K27ac at these promoters showed dynamic changes largely correlating with stage-specific Kdm6b binding, we observed no significant variation in H3K4me3 or H3K4me1 (Fig EV3C). In contrast, distal regulatory elements (intergenic and intronic regions) displayed pronounced dynamics of H3K27me3, H3K27ac, and H3K4me1 (Fig EV3B), highly aligning with that observed at the total binding peaks of Kdm6b (Fig EV3A). Based on these data, we cannot exclude a potential role for promoters in regulating MN developmental progression. However, our findings indicate that **enhancers serve as the primary regulatory regions utilized by Kdm6b to remodel local chromatin landscape dynamics throughout MN development**. This observation aligns with our refined model, which emphasizes a progressive gain of Kdm6b binding at distal regulatory regions rather than a simple promoter-to-enhancer shift. We thank the reviewer for helping us strengthen this aspect of the manuscript.

- At the light of the previous points, the model proposed in Fig.7 is not fully supported by the experimental data

Response: We thank the reviewer for the constructive and insightful comments, which have substantially improved the clarity and logic of our manuscript. Through additional data and detailed clarifications, we have significantly strengthened the overall presentation of this study. Specifically, Fig 2 establishes the dynamic binding pattern of Kdm6b during MN differentiation. Fig 3 demonstrates that Kdm6b occupancy and release correlate with dynamic changes in histone modifications at regulatory regions. Fig 4 shows that key developmental genes are temporally regulated to drive stepwise differentiation. Fig 5 and 6 provide evidence that this temporal control is mediated by stage-specific TFs that associate with Kdm6b at distinct differentiation stages. Collectively, these findings support the proposed model. After careful consideration, we have retained the model schematic (Fig 7) as it effectively integrates the critical roles and mechanisms by which Kdm6b coordinates temporal progression during

stepwise MN differentiation. We believe the revised manuscript now provides adequate support for this model.

- Several evidence from recent articles should be included in the introduction to provide the reader with a more comprehensive view of the literature. These include a series of article on mouse cerebellum development in vivo and on Ezh2/Kdm6b regulation (Matlik et al., PMID: 37491148, Ramesh et al., PMID: 37092728, Tang et al., PMID: 32679064, Wijayatunge et a., PMID: 29254825. Moreover, two recent articles using human stem cell systems have showed that PRC2/H3K27me3 dynamics and other chromatin regulators regulate the timing of neuronal maturation (Ciceri et al., PMID: 38297124) and the temporal control over cell fate specification and neurogenesis across differentiation stages and lineages (Xu et al., PMID: 40897805). These articles should be included in the introduction as well.

Response: We thank the reviewer for providing these valuable references. We have now incorporated all suggested articles into the Introduction to provide a more comprehensive literature background, as recommended.

Patel T, Hammelman J, Aziz S, Jang S, Closser M, Michaels TL, Blum JA, Gifford DK, Wichterle H (2022) Transcriptional dynamics of murine motor neuron maturation in vivo and in vitro. *Nat Commun* 13: 5427

Wang W, Cho H, Lee JW, Lee SK (2022) The histone demethylase Kdm6b regulates subtype diversification of mouse spinal motor neurons during development. *Nat Commun* 13: 958

Dear Prof. Wang,

Thank you for the submission of your revised manuscript. We have now received the enclosed reports from the referees and I am happy to say that all support its publication now. Please address the last referee comments and, importantly, please mention in all relevant figure legends when the data shown are based on a single experiment.

Also a few editorial requests will need to be addressed before we can proceed with the official acceptance of your manuscript:

- Please place the Disclosure and Competing Interest Statement to after the Acknowledgments

- The co-corr. author, Jun-Ma Yu, needs an institutional email address, please add.

- The author credits need to be removed from the ms file. All credits need to be entered during online ms submission.

- Please upload all EV figures as individual, production quality Figure files, without their legends. The EV legends need to be added to the main ms file, after the main figure legends.

- Please remove the Reagents & Tools table from the ms file. It needs to be uploaded as a separate file.

- The Figure legends need to be placed after the References, at the end of the ms file.

* Figure Legends - Comments *

- Please note that the exact p values are not provided in the legends of figures 2C, D, E; 4D, 6E, G; EV2 C-E, please provide exact values as reasonable.
- Please indicate the statistical test used for data analysis in the legends of figures 2G, 4B, S2 A-C; S3A-G
- Please note that the box plots need to be defined in terms of minima, maxima, centre, bounds of box and whiskers, and percentile in the legends of figures 2C-E; EV2 C-E
- Please note that information related to n is missing in the legends of figures 2C-E; EV2 C-E

I would like to suggest some minor changes to the second half of the abstract that needs to be written in present tense. Do you agree with this:

Stage-specific inhibition of Kdm6b compromises the ordered expression of developmental genes. Mechanistically, Kdm6b interacts with temporal TFs over time to ensure precise transcriptional control and MN differentiation. Our work elucidates how a single epigenetic regulator achieves temporal fidelity of stepwise MN development, providing insight into epigenetic regulation of developmental timing.

EMBO press papers are accompanied online by A) a short (1-2 sentences) summary of the findings and their significance, B) 2-3 bullet points highlighting key results and C) a synopsis image that is exactly 550 pixels wide and 300 pixels high. The synopsis image should provide a sketch of the major findings, like a graphical abstract. Please note that text needs to be readable at the final size. Please send us this information along with the final manuscript.

I look forward to seeing a final version of your manuscript as soon as possible.

Best regards,
Esther

Esther Schnapp, PhD
Senior Editor
EMBO reports

Referee #1:

The authors have significantly improved the manuscript and made an attempt to address all points.

Referee #2:

The authors have successfully answered my concerns. Olig2 KD experiments show a nice proof-of-concept to the mechanism proposed in the paper and strengthen the manuscript, and legends have greatly increased in clarity. One thing that remains concerning and is now better noted by the authors in the methods section is that all the CUT&RUN and CUT&TAG are done on n=1 biological experiments on a single mouse embryonic cell line -- how the heterogeneity in this case is not really the biological question being ascertained.

Minor comments:
pg 28: " The Cut&Run and Cut&Tag datas" --- missing end of sentence?

Referee #3:

The authors have addressed my previous concerns.

The authors have addressed all minor editorial requests.

Prof. Wenxian Wang
Anhui Medical University
School of Life Sciences
China

Dear Prof. Wang,

I am very pleased to accept your manuscript for publication in the next available issue of EMBO reports. Thank you for your contribution to our journal.

Your manuscript will be processed for publication by EMBO Press. It will be copy edited and you will receive page proofs prior to publication. Please note that you will be contacted by Springer Nature Author Services to complete licensing and payment information.

You may qualify for financial assistance for your publication charges - either via a Springer Nature fully open access agreement or an EMBO initiative. Check your eligibility: https://link.springer.com/journal/44319/how-to-publish-with-us

Should you be planning a Press Release on your article, please get in contact with embo_production@springernature.com as early as possible in order to coordinate publication and release dates.

If you have any questions, please do not hesitate to contact the Editorial Office. Thank you for your contribution to EMBO Reports.

Best regards,
Esther

Esther Schnapp, PhD
Senior Editor
EMBO reports

>>> Please note that it is EMBO Reports policy for the transcript of the editorial process (containing referee reports and your response letter) to be published as an online supplement to each paper. If you do NOT want this, you will need to inform the Editorial Office via email immediately. More information is available here: https://link.springer.com/partners/embo-press/editorial-policies#Peer%20review

EMBO Press Author Checklist

Corresponding Author Name: Wenxian Wang	
Journal Submitted to: EMBO Reports	
Manuscript Number: EMBOR-2025-62813-T	

USEFUL LINKS FOR COMPLETING THIS FORM
The EMBO Journal - Author Guidelines
EMBO Reports - Author Guidelines
Molecular Systems Biology - Author Guidelines
EMBO Molecular Medicine - Author Guidelines

Reporting Checklist for Life Science Articles (updated January
This checklist is adapted from Materials Design Analysis Reporting (MDAR) Checklist for Authors. MDAR establishes a minimum set of requirements in transparent reporting in the life sciences (see Statement of Task: 10.31222/osf.io/9sm4x). Please follow the journal's guidelines in preparing your
Please note that a copy of this checklist will be published alongside your article.

Abridged guidelines for figures
1. Data
The data shown in figures should satisfy the following conditions:
- the data were obtained and processed according to the field's best practice and are presented to reflect the results of the experiments in an accurate and unbiased manner.
- ideally, figure panels should include only measurements that are directly comparable to each other and obtained with the same assay.
- plots include clearly labeled error bars for independent experiments and sample sizes. Unless justified, error bars should not be shown for technical
- if n<5, the individual data points from each experiment should be plotted. Any statistical test employed should be justified.
- Source Data should be included to report the data underlying figures according to the guidelines set out in the authorship guidelines on Data

2. Captions
Each figure caption should contain the following information, for each panel where they are relevant:
- a specification of the experimental system investigated (eg cell line, species name).
- the assay(s) and method(s) used to carry out the reported observations and measurements.
- an explicit mention of the biological and chemical entity(ies) that are being measured.
- an explicit mention of the biological and chemical entity(ies) that are altered/varied/perturbed in a controlled manner.
- the exact sample size (n) for each experimental group/condition, given as a number, not a range;
- a description of the sample collection allowing the reader to understand whether the samples represent technical or biological replicates (including how many animals, litters, cultures, etc.).
- a statement of how many times the experiment shown was independently replicated in the laboratory.
- definitions of statistical methods and measures:
  - common tests, such as t-test (please specify whether paired vs. unpaired), simple χ^2 tests, Wilcoxon and Mann-Whitney tests, can be unambiguously identified by name only, but more complex techniques should be described in the methods section;
  - are tests one-sided or two-sided?
  - are there adjustments for multiple comparisons?
  - exact statistical test results, e.g., P values = x but not P values < x;
  - definition of 'center values' as median or average;
  - definition of error bars as s.d. or s.e.m.

Please complete ALL of the questions below.
Select "Not Applicable" only when the requested information is not relevant for your study.

Materials

Newly Created Materials	Information included in the manuscript?	In which section is the information available? (Reagents and Tools Table, Materials and Methods, Figures, Data Availability Section)
New materials and reagents need to be available; do any restrictions apply?	Not Applicable	

Antibodies	Information included in the manuscript?	In which section is the information available? (Reagents and Tools Table, Materials and Methods, Figures, Data Availability Section)
For **antibodies** provide the following information:<br>- Commercial antibodies: RRID (if possible) or supplier name, catalogue number and or/clone number<br>- Non-commercial: RRID or citation	Yes	Reagents and Tools Table

DNA and RNA sequences	Information included in the manuscript?	In which section is the information available? (Reagents and Tools Table, Materials and Methods, Figures, Data Availability Section)
**Short novel DNA or RNA including primers, probes**: provide the sequences.	Yes	Reagents and Tools Table

Cell materials	Information included in the manuscript?	In which section is the information available? (Reagents and Tools Table, Materials and Methods, Figures, Data Availability Section)
**Cell lines:** Provide species information, strain. Provide accession number in repository **OR** supplier name, catalog number, clone number, and/**OR** RRID.	Yes	Reagents and Tools Table
**Primary cultures:** Provide species, strain, sex of origin, genetic modification status.	Yes	Reagents and Tools Table, Methods
Report if the cell lines were recently **authenticated** (e.g., by STR profiling) and tested for mycoplasma contamination.	Not Applicable	

Experimental animals	Information included in the manuscript?	In which section is the information available? (Reagents and Tools Table, Materials and Methods, Figures, Data Availability Section)
**Laboratory animals or Model organisms:** Provide species, strain, sex, age, genetic modification status. Provide accession number in repository **OR** supplier name, catalog number, clone number, **OR** RRID.	Not Applicable	
**Animal observed in or captured from the field:** Provide species, sex, and age where possible.	Not Applicable	
Please detail **housing and husbandry conditions**.	Not Applicable	

Plants and microbes	Information included in the manuscript?	In which section is the information available? (Reagents and Tools Table, Materials and Methods, Figures, Data Availability Section)
**Plants:** provide species and strain, ecotype and cultivar where relevant, unique accession number if available, and source (including location for collected wild specimens).	Not Applicable	
**Microbes:** provide species and strain, unique accession number if available, and source.	Not Applicable	

Human research participants	Information included in the manuscript?	In which section is the information available? (Reagents and Tools Table, Materials and Methods, Figures, Data Availability Section)
If collected and within the bounds of privacy constraints report on age, sex and gender or ethnicity for all study participants.	Not Applicable	

Core facilities	Information included in the manuscript?	In which section is the information available? (Reagents and Tools Table, Materials and Methods, Figures, Data Availability Section)
If your work benefited from core facilities, was their service mentioned in the acknowledgments section?	Not Applicable	

Design

Study protocol	Information included in the manuscript?	In which section is the information available? (Reagents and Tools Table, Materials and Methods, Figures, Data Availability Section)
If study protocol has been **pre-registered, provide DOI in the manuscript**. For clinical trials, provide the trial registration number **OR** cite DOI.	Not Applicable	
Report the **clinical trial registration number** (at ClinicalTrials.gov or equivalent), where applicable.	Not Applicable	

Laboratory protocol	Information included in the manuscript?	In which section is the information available? (Reagents and Tools Table, Materials and Methods, Figures, Data Availability Section)
Provide DOI OR other citation details if **external detailed step-by-step protocols** are available.	Yes	Methods, References

Experimental study design and statistics	Information included in the manuscript?	In which section is the information available? (Reagents and Tools Table, Materials and Methods, Figures, Data Availability Section)
Include a statement about **sample size** estimate even if no statistical methods were used.	Yes	Figures
Were any steps taken to minimize the effects of subjective bias when allocating animals/samples to treatment (e.g. **randomization procedure**)? If yes, have they been described?	Not Applicable	
Include a statement about **blinding** even if no blinding was done.	Not Applicable	
Describe **inclusion/exclusion criteria** if samples or animals were excluded from the analysis. Were the criteria pre-established?<br><br>If sample or data points were omitted from analysis, report if this was due to attrition or intentional exclusion and provide justification.	Not Applicable	
For every figure, are **statistical tests** justified as appropriate? Do the data meet the assumptions of the tests (e.g., normal distribution)? Describe any methods used to assess it. Is there an estimate of variation within each group of data? Is the variance similar between the groups that are being statistically compared?	Yes	Figures

Sample definition and in-laboratory replication	Information included in the manuscript?	In which section is the information available? (Reagents and Tools Table, Materials and Methods, Figures, Data Availability Section)
In the figure legends: state number of times the experiment was **replicated** in laboratory.	Yes	Figures
In the figure legends: define whether data describe **technical or biological replicates**.	Yes	Figures

Ethics

Ethics	Information included in the manuscript?	In which section is the information available? (Reagents and Tools Table, Materials and Methods, Figures, Data Availability Section)
Studies involving **human participants**: State details of **authority granting ethics approval** (IRB or equivalent committee(s), provide reference number for approval.	Not Applicable	
Studies involving **human participants**: Include a statement confirming that **informed consent** was obtained from all subjects and that the experiments conformed to the principles set out in the WMA Declaration of Helsinki and the Department of Health and Human Services Belmont Report.	Not Applicable	
Studies involving **human participants:** For publication of **patient photos**, include a statement confirming that consent to publish was obtained.	Not Applicable	
Studies involving experimental **animals**: State details of **authority granting ethics approval** (IRB or equivalent committee(s), provide reference number for approval. Include a statement of compliance with ethical regulations.	Not Applicable	
Studies involving **specimen and field samples:** State if relevant **permits** obtained, provide details of authority approving study; if none were required, explain why.	Not Applicable	

Dual Use Research of Concern (DURC)	Information included in the manuscript?	In which section is the information available? (Reagents and Tools Table, Materials and Methods, Figures, Data Availability Section)
Could your study fall under dual use research restrictions? Please check biosecurity documents and list of **select agents and toxins** (CDC): https://www.selectagents.gov/sat/list.htm	Not Applicable	
If you used a select agent, is the security level of the lab appropriate and reported in the manuscript?	Not Applicable	
If a study is subject to dual use research of concern regulations, is the name of the **authority granting approval and reference number** for the regulatory approval provided in the manuscript?	Not Applicable	

Reporting

The MDAR framework recommends adoption of discipline-specific guidelines, established and endorsed through community initiatives. Journals have their own policy about requiring specific guidelines and recommendations to complement MDAR.

Adherence to community standards	Information included in the manuscript?	In which section is the information available? (Reagents and Tools Table, Materials and Methods, Figures, Data Availability Section)
State if relevant guidelines or checklists (e.g., **ICMJE, MIBBI, ARRIVE, PRISMA**) have been followed or provided.	Not Applicable	
For **tumor marker prognostic studies,** we recommend that you follow the **REMARK** reporting guidelines (see link list at top right). See author guidelines, under 'Reporting Guidelines'. Please confirm you have followed these guidelines.	Not Applicable	
For **phase II and III randomized controlled trials**, please refer to the **CONSORT** flow diagram (see link list at top right) and submit the CONSORT checklist (see link list at top right) with your submission. See author guidelines, under 'Reporting Guidelines'. Please confirm you have submitted this list.	Not Applicable	

Data Availability

Data availability	Information included in the manuscript?	In which section is the information available? (Reagents and Tools Table, Materials and Methods, Figures, Data Availability Section)
Have **primary datasets** been deposited according to the journal's guidelines (see 'Data Deposition' section) and the respective accession numbers provided in the Data Availability Section?	Yes	Data Availability Setction
Were **human clinical and genomic datasets** deposited in a public access-controlled repository in accordance to ethical obligations to the patients and to the applicable consent agreement?	Not Applicable	
Are **computational models** that are central and integral to a study available without restrictions in a machine-readable form? Were the relevant accession numbers or links provided?	Not Applicable	
If publicly available data were reused, provide the respective **data citations in the reference list**.	Yes	References
